# A Lingual Agnostic Information Retrieval System

**DOI:** 10.1155/2024/6949281

**Published:** 2024-07-23

**Authors:** Umar Bn Abdullahi, Godspower Osaretin Ekuobase

**Affiliations:** University of Benin, Benin City, Nigeria

## Abstract

The exclusion of monolingual natives from cyberspace is a global socioeconomic and cultural problem. Efforts at addressing this problem have been socioeconomic, culminating in training, empowerment, and digital access with the indelible hurt of language inequities. This paper is aimed at the cyber-inclusion of monolingual natives. Since cyber participation is basically through human interaction with cyber-applications in a human language, encapsulating these applications for interaction in any human language will help evade the hurt of language inequities. Information retrieval system (IRS) remains a fundamental cyber-application. Consequently, adopting the design science research methodology, we introduced a lingual agnostic IRS architecture designed on the principle of transparency on user language detection, information translations, and caching. The detailed design of the architecture was done using the unified modeling language. The designed IRS architecture has been implemented using the agile and component-based software engineering approaches. The resultant lingual agnostic IRS (LAIRS) was evaluated using heuristics and system evaluation methods for parity of language of interaction against the default language and was excellently stable across queries and languages, guaranteeing 86% parity with the default language in the use of other languages for information access and retrieval. Furthermore, it has been shown that LAIRS is the most appropriate IRS to address the problem of language barriers to cyber-inclusion compared with existing IRSs.

## 1. Introduction

Humans are increasingly dependent on the cyberspace (the Internet and web) for information exchange, business transactions, and social interactions. This has made the cyberspace an invaluable tool for the survival, competitiveness, and socioeconomic development of individuals, organizations, and nations. Despite the inherent heterogeneous language nature of humans, the contents of the cyberspace remain predominantly presented and preserved in few official United Nations (UN) languages [1–4]. This invariably excludes monolingual natives—those that can only read and write in a native language—from participating, contributing, or benefiting from the cyberspace which has become an invaluable part of our world, resulting in socioeconomic deprivations and possible extinction of many native languages.

It is important to note that Internet-underpinned information and communications technologies (ICTs) are the primary drivers of cyberspace [5]. Obviously, if the ICT applications that interface with humans can enable equity in presentations and interactions in the individual human languages irrespective of the original language of preservation of information in the application, endangered native languages will be revived, and the socioeconomic oppression and suppression of the native language speakers will be abated. This study terms such applications lingual agnostic and quickly contrasts them with existing multilingual or cross-lingual applications [3] which compulsorily present information to users in the original language of information preservation and not necessarily in the language of user interaction; and thus, can only be used by multilingual natives for optimum benefit.

In this 21^st^ century, the human resources of any country can only be fully harnessed when a bulk of its population are active cyber-participants. However, it is stifling in the present dispensation to have considerable active cyber-participants in the affairs of a nation when a bulk of its population is excluded from the cyberspace due to language inequities. This is the sympathetic situation of many multilingual nations. Nigeria is a typical economy bedeviled with this problem of language inequities and cyber-exclusion. Nigeria has over 500 endangered languages [4] with less than half of its population literate in the dominant official UN language—the English language [4, 6]. In particular, in the northern part of Nigeria, two of every three are excluded from participating in the cyberspace due to linguistic impediments resulting from their inability to read and write in the English language [7, 8].

Language inequity is a serious problem that has attracted the attention of international bodies such as the United Nations Educational, Scientific, and Cultural Organization (UNESCO). According to [9, 10], more than half of over 7,000 world languages are endangered. This number is highly significant as it translates to 90% of the languages likely to be thrown into extinction by the dominant languages towards the end of the 21^st^ century. It is interesting to note that these native language speakers cannot be tagged as illiterates [11, 12] because a huge number of them can read and write in at least one language though endangered. If this large number of literate populations can be made to participate, contribute, and benefit from the cyberspace in their endangered native languages as their counterparts literate in the official UN languages like English language, it will not only help preserve and promote these languages but also help maximize human potential for socioeconomic development of self and nation. Excluding such a huge population of a nation from participating, contributing, or benefiting from the cyberspace will devalue the economy of that nation and by extension, the world.

Prevalent approaches to addressing this problem have been socioeconomic in nature [13, 14]. However, these socioeconomic approaches which culminate in economic empowerment, training, and digital access/penetration further the neglect of already endangered languages. We assert that lingual agnostic ICT applications that support monolingual natives irrespective of their language are a panacea to these language inequities and cyber-exclusion problem. This assertion is incident on the fact that human participation in the cyberspace is via interactions with the applications that run in the cyberspace and the terminal ICT devices. To the best of our knowledge, such an application or the blueprint for its realization is nonexistent. This problem is therefore desirous of the concerted effort of the design science research community—from the design through development to the deployment of applications that enable monolingual native interactions with ICTs—towards the revival of endangered languages and an all-inclusive socioeconomic world. This study therefore focuses its attention on a mechanism for realizing lingual agnostic ICT applications that support monolingual natives' inclusion in the cyberspace.

An indispensable ICT application that enables human participation in the cyberspace is the Information Retrieval System (IRS). IRS remains the gateway of the cyberspace enabling the retrieval of documents, information, or web services, relevant to a user need, from document repositories or servers [15]. Basically, the existing IRS could be one of the following [16–19]:

- Monolingual Information Retrieval System (MIRS): the query, storage, and presentation languages are the same.
- Cross-lingual Information Retrieval System (CLIRS): the query language is different from the storage and presentation language(s).
- Multilingual Information Retrieval System (MLIRS): the query language, storage language, and presentation language are in multiples.

It was observed that with the existing IRSs, the language of information retrieval (presentation language) is the same as the language of information storage. While the MIRS can completely exclude other language natives from participating in the cyberspace, the CLIRS and MLIRS may allow their inclusion but these users have to be multilingual, i.e., in addition to their native language, they must also be literate in the language of storage (document/information repository), thereby promoting language inequities. However, many MLIRSs leverage parallel corpora to accommodate monolingual natives but this seems foolhardy considering the myriad of natural languages—imagine a cyberspace with 7000 parallel corpora of information it is holding. Furthermore, sophisticated translation methods and pretrained language models are being introduced into CLIRS/MLIRS to support language agnosticism. However, these solutions also suffer from the problem of corpora multiplicity—resource and computational wastages—and tightly coupled information translation and retrieval processes of the system. In practice, many IRSs have already attempted to support monolingual natives of endangered languages across nations by providing translation facilities to search and retrieve information in their native language. However, this translation support is usually not transparent to users and hence hardly useful to natives who cannot comprehend in the default presentation language. Thus, the problem of the exclusion of monolingual natives from the cyberspace is yet to be addressed by existing IRSs. Attenuating the threat of exclusion of monolingual natives from cyber participation towards indigenous language preservation is the motivation of this research.

Consequently, this study specifically dedicates itself to realizing a blueprint for building lingual agnostic IRS (LAIRS) that allows users to query in their native languages and have the required information efficiently presented to them in the same language of the query irrespective of the original language of information storage. Furthermore, the feasibility of the designed lingual agnostic IRS will be established using queries in the dominant Nigerian native languages (Hausa, Igbo, and Yoruba) and UN languages (English language and Arabic) with the language of storage being the English language only.

The remaining part of the paper is organized as follows. The next section examines closely related works and establishes the uniqueness of this work. While Section 3 describes the formulation, detailed design, and implementation of the lingual agnostic IRS architecture, Section 4 describes the evaluation of the resultant IRS system for language parity and its results and interpretation.  Section 5 holds the conclusion and future work.

### 1.1. Contribution

This research paper has contributed to critical domains in the literature: (i) the information retrieval domain—a novel type of IRS named LAIRS, and (ii) the design research domain—a blueprint for building lingual agnostic applications. It is hoped that through adaptation, refinement, replication, and commercialization of the LAIRS architecture, an effective inclusion of monolingual natives in the cyberspace will be unlocked.

## 2. Related Work

Several authors have acknowledged the menace of digital exclusion to the socioeconomic and cultural well-being of people [20, 21]. Predominant solutions have been in the areas of digital access, empowerment, and training [22, 23]. These solutions nonetheless worsen language inequities particularly in the cyberspace. However, the authors of [13, 24] hinted at the design and development of (native language) applications as panacea to the problem of language inequities and digital exclusion of endangered language natives. This study extended this ideology by adopting the design science research methodology (DSRM) [25–27] to realize an architectural blueprint for developing lingual agnostic IRS.

The DSRM is particularly suited for this work because of the nature and novelty of the intended panacea to the problem of cyber-exclusion and language inequities which encapsulates a cyber-application, IRS in particular, such that it will be equally usable in any (preferred) user language. The design and development of IRS predates the Internet and web [28] but the need for an IRS that accommodates natives became popular with the advent of the Internet and web which exposed IRS to information stored in diverse languages across the globe.

One of the earliest attempts at realizing IRS that accommodates natives was the work of Hull and Grefenstette [29]. They proposed an MLIRS that is capable of retrieving documents across language boundaries because of the growing need by many countries to access web resources in their native language(s). Their study established the feasibility of multilingual retrieval by introducing translated queries alongside a bilingual transfer dictionary. However, their work could only establish the feasibility of their idea of MLIRS for just a single language pair—French to English language relying on an English language repository. Their work could not accommodate monolingual natives because search results were not returned in the language of the query. This gap characterized early IRS(s) that attempted to accommodate monolingual natives as evident in [19].

The work of Aljlayl et al. [30] proposed Arabic to English language CLIRS where an Arabic query is used to search information collection in the English language. Their research focused on query translation based on machine translation specifically with ALKAFI Arabic-English machine translation system. Though the accuracy of the retrieved result of their work is high, the retrieved result is in the language of information collection which is different from the language of the native query—Arabic, and thus cannot accommodate monolingual Arabic speakers. Also, Szpektor et al. [18] made an attempt at accommodating Hebrew natives using the English language information repository. Though they realized an IRS, the work also had the drawback of returning search results in the language of information collection only.

The work of Jagarlamudi and Kumaran [31] made an attempt at accommodating the diverse Indian natives in using an English language information repository. Their goal was to retrieve relevant information through queries expressed in different languages in India from an English document collection. Their result showed a significant level of success but like their predecessors, the IRS retrieved result was not in the same language as the native user's query; they were still unable to accommodate monolingual (Indian) natives.

A good attempt at accommodating monolingual natives was also made in [32] by developing a multilingual information repository. This work however failed to appreciate the reality that information is actively dynamic in nature—quantity and freshness—which invariably requires that document translation and index update be done in real time and not preprocessed, as was in their work. Though translating document in real time has been shown to be expensive and slow [8, 30], it is however more effective and realistic particularly with processor efficiency considering the high volume, velocity, and variety of information in modern information repositories.

Inundated by the cost and inefficiency associated with accommodating monolingual natives in the cyberspace, Ragnato et al. [33] introduced the concept of semantic indexing of multilingual corpora to evade, in their words, the heavily impractical task of obtaining and integrating reliable translation models for language pairs. Though their approach may realize a more efficient CLIRS and MILRS, the retrieved result is still in the original language of storage and not in the user query language. This approach, therefore, still cannot accommodate monolingual natives in the cyberspace. Besides, Ragnato et al. [33] assumed a multiple language information repository as in [32] which is not only impracticable for the cyberspace but also not in tandem with the reality of cyber information—high volume, velocity, and variety.

A mixed-language Arabic-English CLIRS model was proposed in [34] to retrieve relevant documents that include mixed-language documents. The classical and current CLIRSs do not address mixed-language document repository where in a corpus, a document can feature on a single page a text in different languages all together (i.e., Arabic and English) especially where technical terms in the scientific domain cannot be expressed in endangered languages. Thus, retrieving such types of documents in a native search query language alone proves difficult. A mixed-language query and cross-language document reweighting approach was used in their work to retrieve most relevant documents. The major limitation of this study is that the proposed system requires the searcher to be either bilingual or multilingual; thus, their IRS is not useful to the monolingual natives.

CLIRS, which allows Indian natives to search English document collections in Tamil native language, was developed. Their work was motivated by the inability of the existing CLIRS translation tool that uses word by word (dictionary-based) translation to properly take care of IR challenges in terms of query ambiguity, out-of-vocabulary words, word inflection, and improper sentence structure. Tamil native language is predominantly spoken by over 75 million people of Indian natives particularly the South Indians. Unfortunately, these natives have poor linguistic competence to formulate search queries in the English language but possess good reading and writing skills in their Tamil native language. Their work, however, does not accommodate monolingual (Tamil) natives who can only search in their native language and accept results in the same language irrespective of the language of the stored document. A similar conclusion also holds for [35], despite the reverse design of their CLIRS, that accepts queries in the English language with the information stored in the native (Hindi) language.

In more recent times, as predicted by Hull and Grefenstette [29], efforts have been directed at realizing information retrieval tasks across language boundaries. Such efforts as evident in [36–41] take advantage of sophisticated machine translation processes, parallel multilingual corpora, and pretrained language models. These efforts may support monolingual natives and help evade the extinction of endangered languages but their translation and retrieval tasks are tightly coupled [39]. Besides, these works are not application development efforts but internal methods or techniques that could be used to fortify the translation and information retrieval processes of an IRS such as the LAIRS proposed in this paper. Our work is an application development effort targeted at realizing a loosely coupled resource efficient translation and retrieval components of a language agnostic IRS. Obviously, loosely coupled systems are easy to build, deploy, reuse, and maintain. Similarly, translation transparency will ease application usage by monolingual natives without multilinguistic abilities while resource efficiency minimizes wastage and usually realizes light weight solutions.

We are aware of the works of Chaware and Rao [42] which proposed a resource efficient LAIRS to support monolingual Indian natives. Although their work is an application development effort, it is vague in design, implementation, and evaluation. Thus, it is not replicable and cannot be said to be successful. We are also aware of the works of Mhaw et al. [43] which employed evolutionary algorithms and realized an IRS with, in their words, “the best accuracy” so far. However, their proposed IRS is MIRS which cannot handle the problem of the exclusion of natives.

Overall, previous application development efforts at accommodating natives in the cyberspace centered on IRS do not allow monolingual natives to retrieve information solely in their native languages through queries submitted in their own native language particularly where the cyber information is not stored in these native languages, thus excluding monolingual natives from the cyberspace.

## 3. Methodology

### 3.1. Architecture Design and Instantiation

This section documents the materials and methods employed in the formulation, design, and implementation of the lingual agnostic IRS. Thus, this section is reported under three subsections: (i) Architecture Formulation, (ii) Architecture Design, and (iii) Architecture Implementation. Also, the resulting artifacts were exposed and discussed in logical flows in their subsections of realization. These design artifacts are technical guides to instantiating the solution blueprints and thus necessary.

#### 3.1.1. Architecture Formulation

This subsection documents a blueprint for developing a lingual agnostic IRS. The introduced software application blueprint or architecture is based on the principle of transparency and caching. The architecture focuses on enabling seamless human-application interaction in the user's own language, for possibly all human languages, irrespective of the default language of interaction and information storage of the application. Thus, of a necessity, such blueprint should have a mechanism for transparently detecting user language and performing translations to give any user the impression that the application is built to be interacted within their own language. Transparency is the concealment of certain physical or logical processes or entities in a software system or application usage from the user or the application programmer [44]. It is a computational principle that improves user convenience and simplicity of interaction with application software.

**Figure 1:**
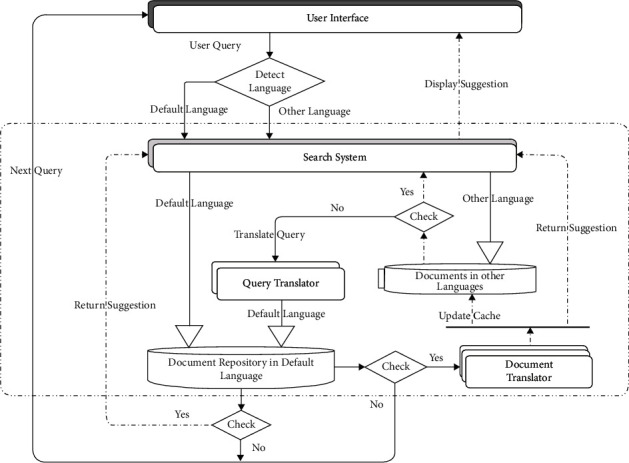
Lingual agnostic IRS architecture (source: authors).

The IRS architecture is designed basically to conceal from the user the fact that the IRS was built to interact in a default language of information preservation and presentation by transparently detecting their language of query and performing to-and-fro translations, as necessary, between the default language and the detected non-default query language behind the scene, i.e., without the knowledge of the user. This offers the advantage of convenience, efficiency, confidence, and evasion of the manual repetitive task of the user having to make use of external translators. Besides, only at least bilingual natives can seamlessly make use of translators external to IRS in the cyberspace. To mitigate the inefficiency resulting from the to-and-fro translations, caching was introduced for possible non-default languages of user query results or suggestions. This IRS architecture that makes any user see the resultant IRS application as if it were designed specifically for their own language and uses it as such is depicted in Figure 1 and termed lingual agnostic IRS architecture.

The architecture in Figure 1 consists of six basic parts: (i) user interface, (ii) language detector, (iii) search system, (iv) translator, (v) document repository, and (vi) document cache, and is discussed as follows:

- *User Interface*. The user interface enables user interaction with the system—query submission to the system and retrieval of suggestions from the system. It also subsumes the language detector component that helps decipher the user query language.
- *Language Detector*. The task of the language detector is to intercept the user query before it gets to the search system, detect the language of the query, and inform the search system of necessary search operations, including translations and presentation of responses in the same user language.
- *Search System*. The architecture's search system is an extended search system whose invocation is dependent on the query language for each supplied query. If the query language is in the default language, it does direct search on the document repository and returns the search suggestions to the user; otherwise, it searches the appropriate language document cache of the query language if similar query formulation suggestion is still cached; else, it then translates the query formulation to the default language, searches the document repository in the default language, translates the search suggestions back to the language of query, and simultaneously updates its language cache and returns the search suggestions to the user in the user's language of query.
- *Translator*. The translator consists of multiple bidirectional language translators between the default language and possibly all other human languages and works under the control of the search system. In Figure 1, the translator component is depicted twice to show the two bidirectional types of translations (default to non-default and vice versa), and the logical position of a language translator instance is invoked, whereas it is a single translator capable of bidirectional translation between the default language and other languages and vice versa.
- *Document Corpus*. The document corpus is simply the full information (document) collection of the search system in the default language. The default language is the prevalent language of information availability. It is then easy to translate the few non-default information or documents to the default language for preservation. This drastically attenuates the unbearable cost and staleness in preserving all information or documents in all possible languages, as is the case with MLIRS.
- *Document Cache*. The document cache consists of language replicas of caches of information or documents initially retrieved for similar queries and non-default language. Cache was particularly introduced to help mitigate the inefficiency resulting from the to-and-fro translations associated with the search requests and suggestions in non-default languages. The caches are light weight and can be updated using any appropriate technique to guarantee freshness.

Although this architecture is specifically tailored to lingual agnostic IRSs, it can be adapted to realize any lingual agnostic application based on the principles of transparent user interaction language detection and translation as exemplified in this subsection.

### 3.2. Architecture Design

The unified modeling language (UML) was employed to translate the lingual agnostic IRS architecture into computational artifacts towards its implementation. UML has become the de facto language for modeling software systems [45]. Generally, the structure and behavior of a software system can be effectively modeled in UML in the four design views of software systems, namely, (i) functional view, (ii) static structural view, (iii) behavioral or dynamic structural view, and (iv) architectural view [46]. In line with a similar work that also modeled a unique IRS architecture [46, 47], this study presents the design of the system implementation of the lingual agnostic IRS architecture in the following notations: use case diagram, class diagram, sequence diagram, activity diagram, component diagram, and deployment diagram. The design was done using Lucidchart (https://lucidchart.com) and Draw.io (https://drawio-app.com). Their choice was based on familiarity, accessibility, and ease of use.

#### 3.2.1. Use Case Diagram

**Figure 2:**
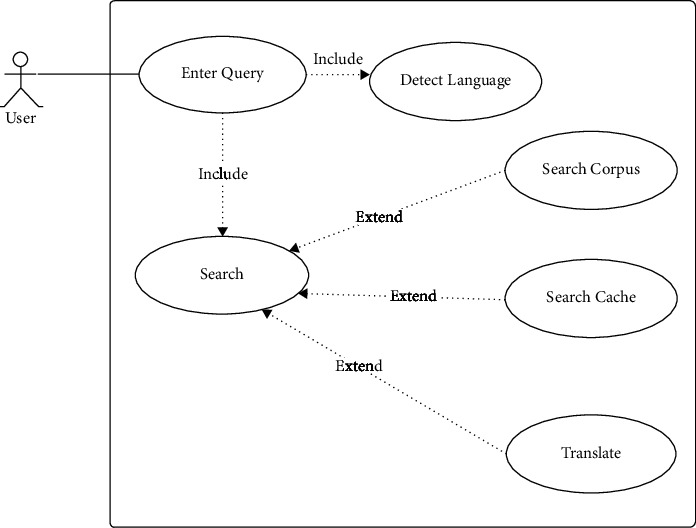
Lingual agnostic IRS use case (source: authors).

A use case diagram is used to describe system functionality from a user's perspective. The use case of the lingual agnostic IRS is modeled as presented in Figure 2. From Figure 2, the user enters a search query which initiates the search process after the query language has been detected. The search process then triggers the corpus search or the cache search depending on the language of query and may have to carry out necessary translations to ensure its search suggestions are in the user's language of query.

#### 3.2.2. Class Diagram

**Figure 3:**
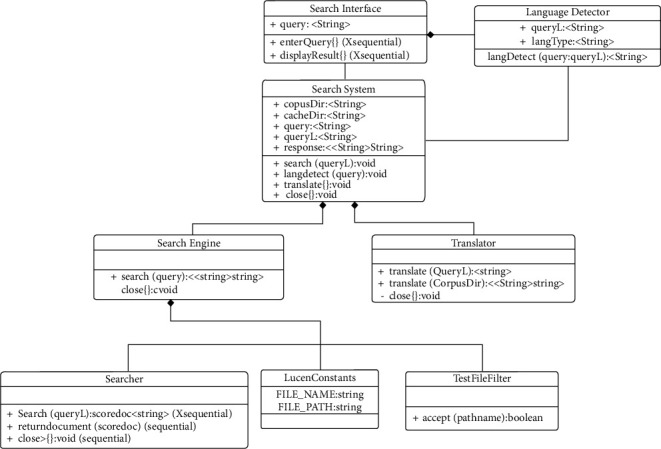
Lingual agnostic IRS class diagram (source: authors).

The class diagram is used to show the static structural view of a system. The class diagram of the lingual agnostic IRS is depicted in Figure 3. Figure 3 consists of eight classes: Search Interface, Language Detector, Search System, Search Engine, Translator, Searcher, LuceneConstants, and TextFileFilter classes and their associations. The Search Interface class accepts user queries and invokes the Language Detector class which detects the language of the query and uses it to decorate the query for onward search processing by the Search System class.

The Search Interface class also displays the search suggestions to the user. The Search System class is composed of the Search Engine and Translator classes. The Translator class can translate the query from its language to English language or translate documents in English language to the language of query. The Search Engine class is composed of the Searcher, LuceneConstants,, and TestFileFilter. The Searcher class performs the indexing, matching, ranking, and retrieval of search suggestions. The LuceneConstant class defines the retrieved document file and path names while the TestFileFilter class ensures that the retrieved documents are of type text (.txt).

#### 3.2.3. Sequence Diagram

**Figure 4:**
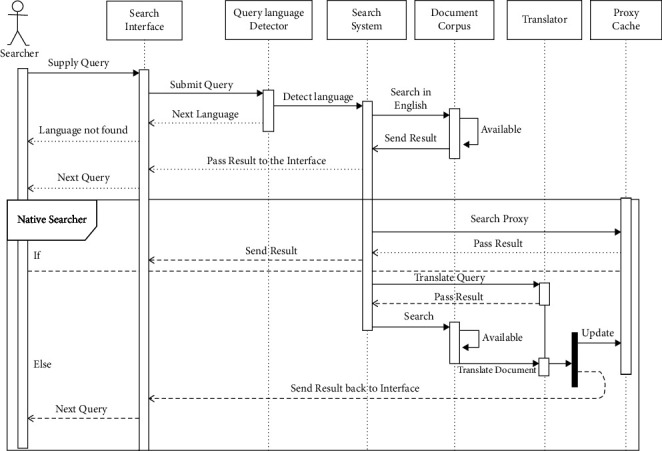
Lingual agnostic IRS sequence diagram (source: authors).

The sequence diagram is used to describe the system's behavior in terms of execution sequence and timeline of liveliness of the individual objects of a system during the execution. The sequence diagram of the lingual agnostic IRS is depicted in Figure 4. The sequence diagram in Figure 4 consists of seven objects including the searcher. As depicted in Figure 4, the Searcher submits query to the Search Interface object which sends it to the Query Language Detector object to decipher and decorate the query with the language of query and transmits to the Search system object to perform the search activities on the Document Corpus object if the language of query is English language and the retrieved search suggestions are transmitted back to the Search Interface object for display to the user.

However, if the language of the query is not English, the decorated query is used by the search system object to search the Proxy Cache object of the language of the query. If matched results are in the cache, the retrieved search suggestions are transmitted back to the Search Interface object for display to the user; otherwise, the decorated query is sent by the Search System object to the Translator object to be translated into English. The Translator object then passes the translated query back to the Search System object to search the Document Corpus object. The retrieved documents are then transmitted to the Translator object for translation into the language of query and simultaneously sent to the Search Interface object and the Proxy Cache object of the language of query for display and caching, respectively.

#### 3.2.4. Activity Diagram

**Figure 5:**
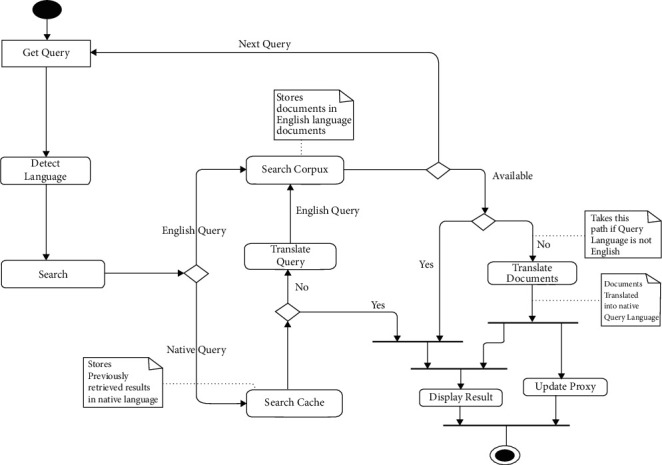
Lingual agnostic IRS activity diagram (source: authors).

While the sequence diagram describes the object execution sequence and liveliness, the activity diagram describes data execution flow and transformation from one process to another in a system from the start to the termination of the system's execution circle. The activity diagram for the lingual agnostic IRS system is depicted in Figure 5. From Figure 5, the activity diagram commences with the Get Query process which captures user query and then the Detect Language process which not only detects the language of the query but also uses the detected language to decorate the user query before onward transition to the Search process.

The Search process transfers control to the Search Corpus process or the Search Cache process depending on the language of the query. If the language of query is English, control is transferred to the Search Corpus process; otherwise, control is transferred to the Search Cache process. The Search Cache process searches and retrieves cached documents for the query from the document cache for the language of query, if they exist, which it transfers to the Display Result process before exiting the system. If they do not exist in the cache, the Search Cache process sends the decorated query to the Translate Query process which translates the decorated query into English and transmits it to the Search Corpus process. The Search Corpus process performs the search activities on the document corpus using the query it receives and transfers retrieved documents to the Display Result process before exiting the system, if the query was originally in English language; otherwise, it sends the retrieved documents to the Translate Documents process. The Translate Documents process translates the retrieved documents to the original language of the query and simultaneously sends the translated version to the Update Proxy and Display Result processes for appropriate cache update and search suggestion display, respectively.

#### 3.2.5. Component Diagram

**Figure 6:**
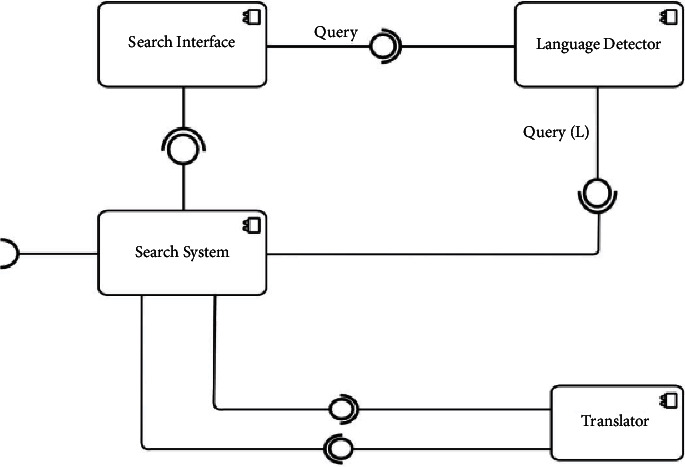
Lingual agnostic IRS component diagram (source: authors).

The component diagram is a high-level description of the system structure in terms of component composition, their interconnectedness, and messaging directions as a black box. The component diagram of the lingual agnostic IRS is depicted in Figure 6. As depicted in Figure 6, the component diagram consists of four components: Search Interface, Search System, Language Detector, and Translator components. The Search Interface component sends Query messages to the Language Detector component and receives search suggestions from the Search System component. The Language Detector component receives Query messages from the Search Interface component and sends language (L) decorated Query, Query (L), to the Search System component. The Search System component sends and receives messages from the Translator component and vice versa. The Search System component also receives retrieved documents from documents repositories which it sends to the Search Interface as search suggestions.

#### 3.2.6. Deployment Diagram

**Figure 7:**
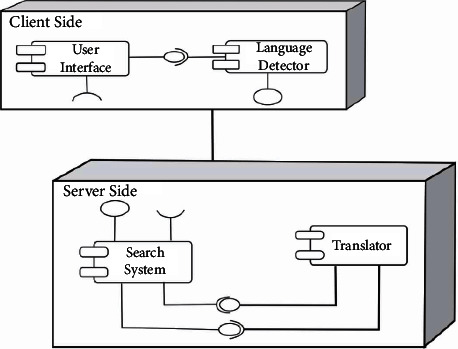
Lingual agnostic IRS deployment diagram (source: authors).

The deployment diagram is used to show how the system components will be configured for operational use. The deployment diagram of the lingual agnostic IRS is depicted in Figure 7. Figure 7 shows the lingual agnostic IRS as a client-server system with the client side consisting of the User Interface and Language Detector components and the server side consisting of the Search System and Translator components, all communicating as shown.

### 3.3. Architecture Implementation

**Figure 8:**
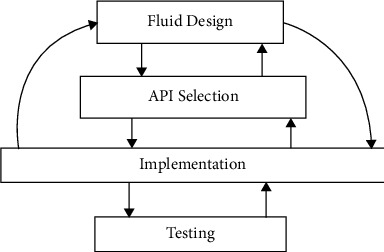
Agile implementation model [49].

The designed lingual agnostic IRS was implemented using the component-based software engineering (CBSE) approach. CBSE is characterized by the selection, reuse, and integration of independently built software components [48]. The reused components can be built from scratch particularly when they are nonexistent, open source, or proprietary. In this study, the search system and language detector components are open source and the translator component is proprietary while the user interface component was built from scratch. Specifically, the CBSE tailored agile approach [49–51] depicted in Figure 8 was adopted for the implementation. Adopting this iterative agile approach was incident on the complexity associated with component selection and integration [48, 52]. The system's design reported in Section 3.2 therefore is the final design of the lingual agnostic IRS.

After a series of iterations and integration testing of selected components or application programming interface (API) on the NetBeans integrated development environment (IDE), the following reused components were selected and integrated alongside the User Interface to realize the resultant lingual agnostic IRS: Google Translate v2 (Translator), Lucene Core version 3.6.2 (Search System), and DetectLanguage API version 1.1.0 (Language Detector). The reusable components used were selected based on documentation, ease of use, portability, reliability, generality, and mutual compatibility. The NetBeans 8.1 IDE was employed as the IDE of choice due to familiarity, ease of use, and robustness. The User Interface component of the IRS and its knitting with other selected components was done using the Java programming language, the default language of the NetBeans IDE.

The resultant lingual agnostic IRS was developed, tested, and run on 70 carefully curated user queries in the five selected human languages and a corpus of 86 English language documents stored as .txt flat files, all hosted on a 64 bit Intel® Core ™ i5–2540M @2.60 GHz 2.60 GHz Windows 10 Pro DELL (DESKTOP-BBLD6FK) Personal Computer with 4.00 GB RAM. For reproducibility and possible improvements, the source code of the implementation is in tswj-6949281-sup-01-LAIRSourcecode.doc (supplementary material (available here)). The queries and their search suggestions in the five selected human languages are presented, analyzed, and discussed in the next section.

## 4. Results and Discussion

### 4.1. Experimental Evaluation

This section documents the system evaluation for parity of search precision, recall, and F-measure of the lingual agnostic IRS on a document corpus for a set of queries in the English language and their translated equivalents in Arabic, Igbo, Hausa, and Yoruba. Heuristic and system evaluation techniques [53–56] were employed and the results were further summarized using line graphs. The document corpus was a set of 86 journal articles in .txt, serially numbered, from the BADALA Journal. The BADALA journal was chosen because of the familiarity of our language experts with the journal both as authors and readers. To evaluate the IRS system, a set of 70 information needs and their equivalent queries (or topics) in the English language as shown in Table 1 were carefully curated. A minimum of 50 test topics is enough for obtaining reliable IRS evaluation results [57, 58].

The queries were manually translated into Hausa, Igbo, Yoruba, and Arabic by the language experts and authenticated by different language experts. These language experts and their peer authenticators are of the School of Nigerian Languages, Federal College of Education, Kano, each with a minimum of MA and five years of teaching, writing, and speaking experience in the respective languages. The translated queries in the various languages are presented in the same order as their English language counterpart as shown in Table 2.

Thereafter, a system search was performed on the designed IRS using the 70 queries one after another for the English language and each of the translated equivalents for the four selected indigenous languages. The IRS search suggestions (document identifiers) for the queries in each of the languages are captured in Table 2 as retrieved. The search suggestion documents in each of the translated languages were then presented to the language experts for each of the language as translated vis-à-vis the original English language document for authentication. They all agreed that the translations were in the correct language and semantically correct but not perfect. This is not strange as no machine translation can be as perfect as human expert translation [59–61].

It is worth a quick note that a given indigenous search operation took between 12 and 15 minutes to complete on first instance depending on the number of documents retrieved and network strength as the translator component was remotely consumed but subsequently (i.e., after caching) took less than 30 seconds as their English language counterparts. This underscores the importance of caching in the architecture.

From the results in Table 2, the lingual agnostic IRS search precision@10, recall@10, and F-measure@10 were computed using equations (1)–(3) [46, 57], respectively, for each query in the five languages including the English language as captured in Table 3, and the summary (averages) is presented in Table 4. The use of the first ten retrievals per topic search is common and effective in practice for evaluating IRS [58].

The relevant documents for each query in the repository as identified in the English language context and shown in Table 3 were used in this study as the gold standard since the study is about parity of interaction between the indigenous language users and the English language user. Thus, relevant document retrieved (RDR) in this study is a function of what is relevant in the default language of the document repository which is the English language.

(1) $$\begin{array}{c} \text{Precision}@10=\frac{\text{Number of relevant documents retrieved in the first ten retrievals }\left(\text{RDR}@10\right)}{\text{Total number of documents retrieved }\left(i.e.10\right)}, \end{array}$$

(2) $$\begin{array}{c} \text{Recall}@10=\frac{\text{Number of relevant documents retrieved in the first ten retrievals }\left(\text{RDR}@10\right)}{\text{Total number of Relevant documents }\left(\text{RD}\right)\text{ in repository }}, \end{array}$$

(3) $$\begin{array}{c} F-\text{measure}@10=2*\frac{\text{Precision}@10\;*\text{ Recall}@10}{\text{Precision}@10+\text{Recall}@10}. \end{array}$$

**Figure 9:**
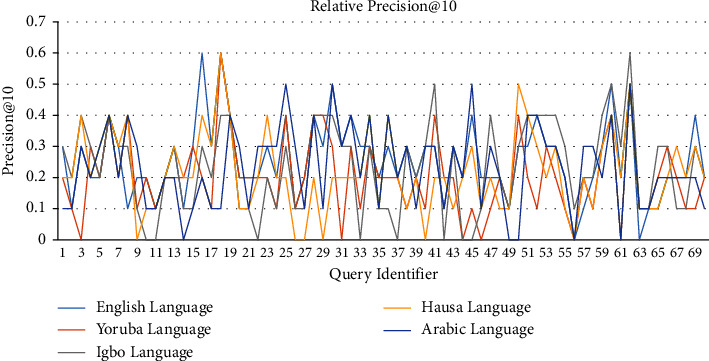
Lingual agnostic IRS precision@10 line graph for selected language topics (source: authors).

**Figure 10:**
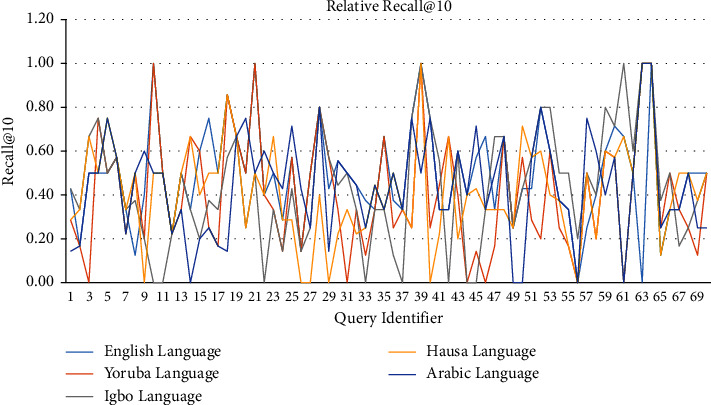
Lingual agnostic IRS recall@10 line graph for selected language topics (source: authors).

**Figure 11:**
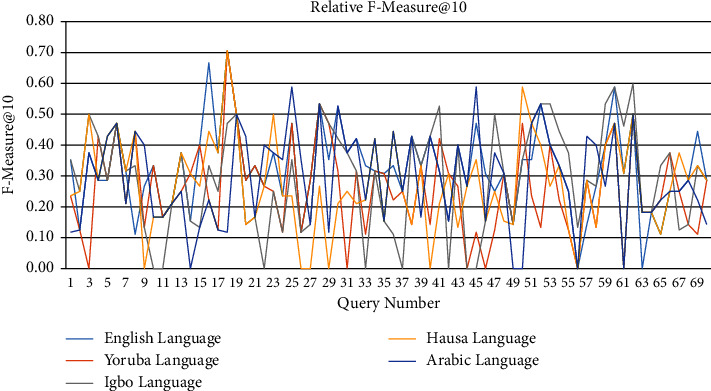
Lingual agnostic IRS F-measure@10 line graph for selected language topics (source: authors).

Table 4 holds the average precision@10, recall@10, and F-measure@10 of the individual languages. From Table 4, a line graph of precision@10 of each query for each of the five languages is captured as shown in Figure 9. Similarly, Figures 10 and 11 hold the line graphs of recall@10 and F-measure@10, respectively. From Tables 3 and 4 and Figures 9 and 10, it is evident that LAIRS precision@10 ranges from 0.0 to 0.6 with an average of 0.23, recall@10 ranges from 0.0 to 1.0 with an average of 0.44, and F-measure@10 ranges from 0.0 to 0.7 with an average of 0.30. These signal higher recall than precision in the performance of LAIRS across languages and queries in practice. Thus, users of LAIRS are guaranteed that close to half of the relevant documents retrieved in the first ten retrieved documents in a search operation, if they exist in the repository, are relevant.

To determine the parity of selected indigenous language with English language using the lingual agnostic IRS, the study assumed the English language results as the gold standard. To obtain language parity, the ratios of the average search precision@10, recall@10, and F-measure@10 for each indigenous language to those of the English language expressed as percentages were computed and are presented in Table 4. The term “overall” as used in Table 4 depicts the average percentage parity of precision@10, recall@10, or F-measure@10 of the indigenous languages with the English language and, thus, represents the search retrieval performance of the IRS for non-English language speakers. In particular, the overall parity F-measure is taken as the designed system language parity measure. The F-measure value is often the preferred retrieval reliability measure for IRS because it mitigates the effect of outliers in both precision and recall values. In the same vein, the overall parity F-measure is a reasonable measure of the lingual agnostic IRS.

**Figure 12:**
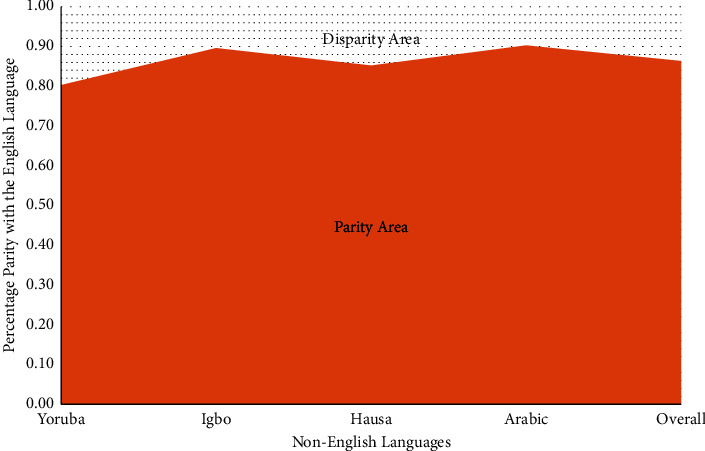
Area graph of the lingual agnostic IRS language interaction parity (source: authors).

Finally, the average parities of F-measure computed for each of the non-English languages and the overall F-measure as well were depicted as an area graph to show the parity (disparity) of interaction of English language vis-à-vis other languages using the LAIRS, as presented in Figure 12. While the line graph helps to show the retrieval trend over query, the area graph shows the space in the whole that non-English language users are in parity or disparity with the English language users while interacting with LAIRS. The graphs and computations were drawn/computed using the Microsoft Excel Spreadsheet package. The Excel spreadsheet satisfactorily captures our intended message; it is available and easy to use, hence its choice.

### 4.2. Interpretation of Results

It is important to appreciate that the essence of the evaluation is to determine the parity of interaction with the IRS using non-English language vis-à-vis using English language. The number of queries was limited to 70, a decision made to ensure the minimum 50 required to reliably evaluate an IRS [57, 58] is exceeded. This limit also helps to avoid overburdening the language experts, who have to manually examine each piece of information and query results from about 86 journal articles for relevance in retrieval and presentation. The number of indigenous languages considered by this research was incident on the indigenous languages in the use environment of study with standard translator components.

The system evaluation results in Table 4 show that the lingual agnostic IRS is an excellent IRS with equitable information retrieval across indigenous languages. This was also the case for execution efficiency as noted in Section 4.1. Ordinarily, on real-life development/deployment, the IRS translator(s) will not be remotely consumed and if at all, with minimal latency; thus, the initial efficiency hiccup experienced before cashing will not be pronounced as in this experimental case particularly as with ultra-fast servers.

The implication of this is that, overall, the IRS is excellently stable across queries and languages. The language disparity in the designed lingual agnostic IRS as evident in Tables 3 and 4 and Figure 12 can be traced to the translator imperfection as exposed in [62, 63]. The implication is that as the indigenous language translators improve in accuracy, the lingual agnostic IRS will definitely improve in parity of interaction with the non-default languages as with the English language. Table 4 makes it evident that the lingual agnostic IRS guarantees a parity of 86% with the English language irrespective of the language of interaction. This parity of language interaction can be as high as 90% for some languages and even higher. The implication is that the IRS is capable of mitigating the language inequities bedeviling cyber-participation by about 90%, i.e., with such IRS deployed, monolingual natives can participate, contribute, or benefit from the cyberspace with less than 10% additional strain as their English language counterparts. This fact is however not foolproof due to the limited number of languages used.

### 4.3. Comparative Analysis

To address the problem of language barriers to cyber-inclusion, a basic IRS—the lingual agnostic IRS (LAIRS)—has been introduced. The LAIRS is a basic IRS designed to accept queries in any detectable language and return search results in the language of query irrespective of the language of storage. However, for pragmatic concerns, it is necessary to comparatively analyze LAIRS against existing IRSs—MIRS, CLIRS, and MLIRS—to showcase LAIRS's effectiveness and efficiency to address the problem of language barriers to cyber-inclusion against the current IRS solutions. To this end, six criteria of pressing interest to present users of IRS in the cyberspace were identified to further discussion: (i) inclusivity, (ii) effectiveness, (iii) relevance, (iv) efficiency, (v) effort, and (vi) sustainability. These criteria are examined as follows:

- *Inclusivity*. The inclusion of all humans in the social, economic, and political affairs of humanity increasingly driven by the cyberspace is a global mandate [64]. LAIRS and MLIRS have excellent inclusivity as they can accommodate any user including monolingual natives. However, while MLIRS incurs exponential storage costs with increasing number of corpus documents (N) and increasing number of detectable languages (L) (i.e., N × L storage cost) to enable inclusivity due to parallelism of corpus, the LAIRS storage cost only increases linearly with N. Therefore, the inclusivity cost of LAIRS can be said to be linear while that of MLIRS is exponential. The inclusivity strengths of both LAIRS and MLIRS are however limited by the availability of resources and tools for language detection and translation. The CLIRS inclusivity is seriously limited and can best be rated as fair because it allows user queries in any detectable language but returns results in the original language of information storage which may not be the language of user queries. The MIRS obviously excludes all but one set of monolingual natives since the language of user query must be the same as that of information storage. Overall, LAIRS best supports inclusivity in addressing the problem of language barriers to cyber-inclusion.
- *Effectiveness*. The effectiveness of an IRS is commonly defined by its precision (the amount of retrieved documents that were intended) and recall (the amount of intended document that were retrieved). Here, MIRS stands out as it requires no language translation. Information loses accuracy when translated from one language to another; however, excellent the translator. CLIRS may only require user query translation while LAIRS and MLIRS may require both user query translation and document translation. Hence, their effectiveness is limited and dependent on the quality of translation of user queries and retrieved documents/information. Consequently, the effectiveness of LAIRS and MLIRS in addressing the problem of language barriers to cyber-inclusion is dependent on the accuracy and reliability of the individual language translators which in turn depends on the efficacy of the linguistic resources available to the individual languages. This accounts for the varying performance across the different languages (language bias) in this study despite the queries having the same meaning and intent. Continuous investments and improvements in individual language linguistic resources and natural language translators particularly for the low resource languages will bring about improved retrieval equity and effectiveness of LAIRS for improved cyber-inclusion. Overall, LAIRS is limited by retrieval effectiveness in addressing the problem of language barriers to cyber-inclusion.
- *Relevance*. A retrieved document even when it is the expected document may be presented in another language or format incomprehensible to the user. Such documents will not be useful to the user due to language barriers and hence not relevant. This will rarely occur with MIRS and LAIRS since retrieval results are compulsorily returned in the language of the user query. However, this is always the case with MLIRS, though not necessarily so, despite the implementation strategy–MLIRS supports multilinguistic documents or users. Hence, we can state that MIRS and LAIRS are of excellent relevance while MLIRS can be rated fair in terms of relevance. However, CLIRS has low relevance because irrespective of the language of user query, the retrieved document is returned in the language of information/document storage. Overall, LAIRS best supports relevance in addressing the problem of language barriers to cyber-inclusion.
- *Efficiency*. Efficiency in computing is the reasonable or (near) optimum use of computer resources—time, space, and computational overhead—in execution to perform a given task which in this case is an information search and retrieval task. When an application is wasteful in the utilization of these computer resources, it does manifest in time and/or space consumption. Hence, the complexity of computer algorithms till date is determined by time and space complexities. Obviously, MIRS will be most efficient in terms of response time of the four basic IRSs because it requires no translation or additional task outside common retrieval and specific implementation tasks. This will be closely followed by CLIRS that does user query selection only and can be rated as good in efficiency. MLIRS, which may involve both user query and document translation, does its document translation offline, i.e., preprocessed, unlike LAIRS, whose corpora translation is done in real-time of execution of user search instruction except the hit was found in the cache. Thus, we can conclude that LAIRS has the worst time efficiency than all other basic IRSs in addressing the problem of language barriers to cyber-inclusion. In terms of space consumption, the MIRS and CLIRS corpora have no multiple language replicas though they may be replicated or distributed to meet non-functional requirements and are thus excellently efficient in terms of space. This is not the case with MLIRS usually with parallel corpora which can also be replicated or distributed. Thus, we conclude that MLIRS is poorly efficient in terms of space consumption. The LAIRS only has a parallel cache corpus, which is usually lightweight compared to permanent repositories. LAIRS therefore can be said to have good space efficiency, a better space efficiency than MLIRS though not as good as those of MIRS and CLIRS in terms of space consumption. The MIRS is also the best in computational overhead, followed closely by CLIRS and then MLIRS. The CLIRS and MLIRS additional retrieval task in a retrieval operation has to do with query translation though MLIRS may involve additional language tuning tasks. The LAIRS is the worst in terms of computational overhead because it does bidirectional translation online, i.e., in real time on demand of a retrieval task. Overall, we conclude that LAIR is poorly efficient in addressing the problem of language barriers to cyber-inclusion. However, with the increasing computer speed and faster broadband, this defect will be attenuated.
- *Effort*. This is the user knowledge or capabilities required to use the IRS solutions from the respective IRSs. The CLIRS requires the user to be at least bilingual making it almost impossible for any user defective in the language of storage to use CLIRS solutions. This language restriction is worst with MIRS where the user is restricted to a single language. Most MIRS and CLIRS solutions particularly in the cyberspace now provide external language detection and/or translation provision for users to externally select their user search language and/or translate retrieved documents to their language of convenience. It is trivial to state that these external provisions are usually in a mono language which may not even be comprehensible to a user not knowledgeable in that language. This makes the user effort particularly as it affects linguistic capabilities in cyber-inclusion to be high for MIRS. MLIRS and LAIRS require the least user effort as it affects linguistic capabilities in cyber-inclusion. However, the maintenance effort of MLIRS is high as each document must be translated offline to N–1 corpus for N parallel corpus of a MLIRS unlike with LAIRS whose translation is done real time transparently on-demand, if at all required, i.e., if not already in the required language cache. Overall, LAIRS best supports user effort in addressing the problem of language barriers to cyber-inclusion.
- *Sustainability*. Sustainability is an issue of critical global concern. In the context of this discussion, sustainability is the capacity of IRS solutions to consistently and equitably support the increasing size of corpus and incorporate the growing number of languages with linguistic resources and translation solutions in the cyberspace. MIRS and CLIRS are obviously not in for consideration under sustainability as their IRS solutions cannot scale in the dimension of increasing language support despite their capacity to scale linearly with increasing size of corpus. Although LAIRS and MLIRS solutions have the capacity to scale in both dimensions of corpus and languages, the corpus parallelism feature of MLIRS casts aspersions on its capacity to scale with increasing corpus size (N) and number of languages (L). For as N ⟶ ∞ and *L* ⟶ ∞, the storage demand (S) of MLIRS will be impractical to maintain since parallelism implies S::MLIRS ⟶ N × L. However, the storage demand of LAIRS will behave as those of MIRS and CLIRS which grows linearly with N particularly with a very large N as with the cyberspace. The additional space demand of LAIRS against those of MIRS and CLIRS overtime as the number of languages increases is the number of cache which is usually lightweight and of fixed size (*α*). Thus, S::LAIRS ⟶ N + *α*L < 2N. Overall, LAIRS best supports sustainability in addressing the problem of language barriers to cyber-inclusion.

Conclusively, LAIRS has shown superior prospects of resolving the problem of language barriers to cyber-inclusion than any of the existing IRSs: MIRS, CLIRS, and MLIRS.

## 5. Conclusion and Future Work

The problem of language inequities and cyber-exclusion has received considerable socioeconomic (behavioral science research) attention producing effective solutions. However, these solutions which can be grouped under training, empowerment, and digital access/penetration strengthen language inequities, thereby facilitating the extinction of many native languages. To complement this behavioral science research effort, the work examined the dual problem from the built perspective and addressed the problem via an alternate information system research paradigm—the design science research paradigm.

This research posture was incident on the obvious fact that human interaction with the cyberspace is via interaction with the applications that run on the cyberspace and the terminal ICT devices. The research then nursed a novel idea of lingual agnostic cyber applications as a panacea to the problem of cyber-exclusion and language inequities. These applications vary but the most fundamental of them is the IRS. Consequently, the built idea was situated in the information retrieval domain for purposes of instantiation and experimentation. The built idea is simply based on the principle of transparency of user language detection and message translations as well as caching to mitigate the inefficiency associated with information translation.

Obviously, the design science research methodology is most appropriate for built ideas and was thus adopted for this research. The research has introduced a blueprint or architecture, hitherto not existing, for developing lingual agnostic IRS—a unique type of IRS. This blueprint has been designed using UML in the four facets of system design. Adopting the agile and CBSE approaches, the study has developed a functional prototype of the IRS. This IRS has been evaluated for parity of interaction with monolingual natives vis-à-vis the default application language users using the heuristic and system evaluation methods. The result is that the IRS is excellently stable across languages and queries with language interaction parity as high as 90% in the use of indigenous languages for information access and retrieval. With such IRS deployed, monolingual natives can participate, contribute, or benefit from the cyberspace with less than 10% additional strain as their default language counterparts. Furthermore, it has been shown that LAIRS is the most appropriate IRS for addressing the problem of language barriers to cyber-inclusion compared to existing IRSs: MIRS, CLIRS, and MLIRS.

For improved language interaction parity and to accommodate more monolingual natives in the cyberspace, existing translators must improve their accuracy, while for those languages without standard language translators, there should be urgent investments on efforts towards the language formalism and bidirectional translation with other languages. The capability of the proposed lingual agnostic IRS architecture needs to be tested further on more diverse languages. Also, an ablation study should be carried out on the proposed architecture with a view to realizing the existing component combination set for optimal language parity. These are urgent calls to rescue endangered languages and boost cyber-participation.

## Figures and Tables

**Table 1:** Curated information needs and queries (topics).

S/N	User information requirement	Search query (topics)
1	Assessing the Roles of Language and Communication in Transforming and Sustaining National Development in Nigeria	Assess and Role and Language and Communication and Transform and Sustain and National and Development and Nigeria
2	The Importance of Maintenance Culture in the Management and Growth of Organizations in Nigeria	Importance and Maintenance and Culture and Management and Growth and Organization and Nigeria
3	The Role of Islamic Studies Teachers in the Quality Control of Education in Nigeria	Role and Islamic and Studies and Teacher and “Quality Control” and Education and Nigeria
4	Leadership in Sports Management Plays an Integral Role in the Development of Sports Activities in Nigeria	Leadership and Sport and Management and Play and Role and Development and Nigeria
5	Science Education and Good Governance in Nigeria	Science and Education and Good and Governance and Nigeria
6	Islamic Education as a Panacea to Corruption in Nigeria	Islamic and Education and Panacea and Corruption and Nigeria
7	Social Studies Education and the National Security Transformation Agenda	“Social Studies” and Education and “National Security” and Transformation and Agenda
8	Islamic Education as a Panacea to Corruption in Nigerian Economy	Islamic and Education and Panacea and Corruption and Nigeria and Economy
9	Transformation Agenda: Key to Economic Development in Nigeria	Transformation and Agenda and “Economic Development” and Nigeria
10	Estimation of Percentage of Calcium in Some Tap Water Samples	Estimation and Percentage and Calcium and “Tap Water”
11	Functions of National Commission for Colleges of Education (NCCE) towards Quality Teacher Education in Nigeria	Functions and “National Commission” and College and Education and NCCE and Teacher and Nigeria
12	Socio-Cultural Literacy Education as Precursor to National Security Management and National Transformation Agenda in Nigeria	Socio-Cultural and Literacy and Education and “National Security” and Management and Transformation and Agenda and Nigeria
13	National Security, Human Rights Protection, and Social Transformation	“National Security” and “Human Right” and Protection and Social and Transformation
14	The Importance of Games, Role Play, and Debate as Teaching Techniques in Social Studies	Importance and Games and “Role Play” and Debate and Teaching and Technique and “Social Studies”
15	Potentials of Theatre Arts in Empowering Youths for National Stability	Potential and “Theatre Arts” and Youth and National and Stability
16	Social Studies Education as a Tool for Economy Transformation and Self-Reliance	“Social Studies” and Education and Tool and Economy and Transformation and “Self-Reliance”
17	The Dynamics of Inter-Group Relations in Multicultural Society like Nigeria	Inter-Group and Multicultural and Society and Nigeria
18	Women in Sports: Gender Stereotypes in the Past and Present	Women and Sport and Gender and Stereotype
19	Youth Empowerment: A Remedial Action for Youth Involvement in Kano Conflict for National Stability	Youth and Empowerment and Kano and Conflict and National and Stability and Involvement and Remedy
20	The Challenges of Constitutional Reform, Economic Depression, and Nigeria's Quest for National Birth	Challenge and Constitution and Reform and “Economic Depression” and National and Nigeria
21	The Changing Political Map of Nigeria from 1960 to Date and National Rebirth	“Political Map” and Nigeria and 1960 and “National Rebirth”
22	Geography Education and Natural Resources Management for National Rebirth in Nigeria	Geography and Education and “Natural Resources” and Management and “National Rebirth” and Nigeria
23	Towards an Effective History Education for National Rebirth	“History Education” and “National Rebirth” and Effective
24	The Relevance of Nigeria's Historical Experience for National Rebirth	Nigeria and “Historical Experience” and “National Rebirth” and Relevance
25	The Global Economic Depression of the 1930s Lessons for Nigeria	Global and “Economic Depression” and Lesson and 1930 and Nigeria
26	Attitudinal and Value Reorientation: A Sine qua non for National Rebirth and Development	Attitude and “Value Reorientation” and Sinequanon and “National Rebirth” and Development
27	Refocusing Political Education for National Security and Nigeria Rebirth	“Political Education” and “National Security” and Nigeria and Rebirth
28	The Level of Youth Political Awareness and Participation: Implication for National Stability	Youth and “Political Awareness” and Participation and Implication and “National Stability”
29	Social Studies Education, as an Instrument for Civic Responsibility, Economic Efficiency, and National Rebirth	“Social Studies” and Civic and Responsibility and Education and Instrument and “Economic Efficiency” and “National Rebirth”
30	Achieving Constitutional Reform for Economic Stability and National Rebirth in Nigeria Using Social Studies Education as a Means	Constitution and Reform and “Economic Stability” and “National Rebirth” and Nigeria and “Social Studies” and Education
31	Underdevelopment and Economic Depression in Nigeria: A Transformation of Nigeria's Human and Natural Resources	Underdevelopment and “Economic Depression” and Nigeria and Transformation and Human and Natural and Resources
32	Population, Family Life Education, Constitutional Reform, Economic Depression, and National Rebirth	Population and “Family Life” and Education and Constitution and Reform and “Economic Depression” and “National Rebirth”
33	The Menace of Human Trafficking in Nigeria: Implication for Social Studies Education	Menace and “Human Trafficking” and Nigeria and Implication and “Social Study” and Education
34	Islamic Point of View on Constitutional Reform, Economic Depression, and National Rebirth	Islamic and Constitutional and Reform and “Economic Depression” and “National Rebirth”
35	Problems of Teaching and Learning Islamic Studies in Colleges of Education: A New Direction for National Development	Problem and New and Direction and Teaching and Learning and “Islamic Studies” and College and Education and National and Development
36	The Church and Citizenship and Leadership Education for National Rebirth	Church and Citizenship and leadership and Education and “National Rebirth”
37	Determinants of Parental Influence on Child in Their Youth Age and Sports Participation	Parent and Child and Influence and Youth and Sport and Participation
38	Role of Physical Activity in Preventing and Treating Overweight and Obesity	Role and “Physical Activity” and Treat and Prevent and Overweight and Obesity
39	Information Communication Technology in the Management and Administration of Sports in Tertiary Institutions	Information and Communication and Technology and Management and Administration and Sport and Tertiary and Institution
40	Global Economic Melt Down Vision 2020 and Geography Education	Global and Economy and “Vision 2020” and Geography and “Melt Down” and Education
41	The Roles of Islamic Teachings in Economic Development of Nigeria	Role and “Islamic Teaching” and Economy and Development and Nigeria
42	Hitches in the Organization and Administration of Guidance Services in Nigerian Secondary Schools	Hitch and Organization and Administration and Guidance and Nigerian and “Secondary School” and Service
43	Activity-Based Method for Effective Teaching and Learning Science in Secondary Schools	Method and Activity and Effective and Teaching and Learning and Science and “Secondary School”
44	The Importance of Mother Tongue in the Context of Nigeria's Aspiration for Economic Development	Importance and “Mother Tongue” and Nigeria and Aspiration and Economy and Development
45	An X-Ray of Secondary School Management, Constitutional Reforms, Economic Depression, and National Rebirth in Nigeria	“Secondary School” and Management and Constitution and Reform and “Economic Depression” and “National and Rebirth” and Nigeria
46	The Emerging Roles of the Society in the Implementation of the Universal Basic Education (UBE) in Nigeria in the 21st Century	Emerging and Role and UBE and Society and Implementation and “Universal Basic” and Education and Nigeria and “21st Century”
47	The Quest for a Credible Electoral System and Sustainable Democracy in Nigeria: Imperatives of Constitutional Reform	Credible and “Electoral System” and Sustainable and Democracy and Nigeria and Constitution and Reform
48	The Biography and Contribution of Islamic Scholars to Knowledge and Intellectual Achievement of Muslim Society	Biography and Contribution and “Islamic Scholar” and Knowledge and “Muslim Society”
49	Practicability of Activity-Based Costing System in the Nigeria Transport Industries	Practicability and System and Nigeria and Transport and Industry
50	Islamic Solution to Economic Depression and Poverty Alleviation	Islamic and Solution and “Economic Depression” and Poverty
51	A Collaborative Approach to the Management of Women Education for Sustainable National Development	Collaboration and Approach and Management and Women and Education and Sustainable and National and Development
52	A Profile Appraisal of Part-Time Adult Education Programme in Geography	Appraisal and Part-Time and “Adult Education” and Programme and Geography
53	Agriculture: A Key Element for Facilitating Growth and Stability of Nigerian Economy	Agriculture and Element and Facilitate and Growth and Stability and Nigeria and Economy
54	Adult Education and Poverty Alleviation for Sustainable Economic Development	“Adult Education” and Poverty and Sustainable and Economy and Development and Nigeria
55	An Examination and Analysis of Nigerian Economic Structure and Organization in Light of Historical Developments	Examination and Analysis and Nigeria and “Economic Structure” and Organization and “Historical Development”
56	A Review of the Vital Role Played by Arabic Education towards National Stability Right from Nigerian Pre-Independence Era	Role and Arabic and Education and National and Stability and Nigeria and Pre-Independence
57	HIV and AIDS Awareness and Attitude of Female Students towards Sexual Behavior	HIV and AIDS and Awareness and Female and Student and Attitude and “Sexual Behavior”
58	Water Security Challenges and National Transformation Agenda in Nigeria	“Water Security” and Challenge and National and Transformation and Agenda and Nigeria
59	The Imperatives of Drug Education on Leadership and Good Governance in Nigeria	Drug and Education and Leadership and Governance and Nigeria
60	The Role of Christian Women in Enforcing Morals in the Society	Role and Christian and Moral and Enforce and Women and Society
61	An Examination of the Major Challenges Confronting Good Governance and Nigerian Economy	Examination and Challenge and Governance and Nigeria and Economy
62	Citizenship Education and Youth Empowerment for National Stability	“Citizenship Education” and Youth and Empowerment and National and Stability
63	Contemporary Issues in Education: Enhancing the Millennium Development Goal through Islamic Curriculum of Education	Contemporary and Education and Millennium and Enhance and Development and Goal and Islamic and Curriculum and Education
64	Continuous Assessment and Examination Malpractice in Nigerian Federal Colleges of Education	Continuous and Assessment and Examination and Malpractice and Nigeria and Federal and College and Education
65	National Security and the Transformation Agenda in Nigeria: Issues and Challenges	“National Security” and Transformation and Agenda and Issues and Challenge and Nigeria
66	Enhancing National Security in Nigeria Through Good Governance	“National Security” and Nigeria and Governance
67	Colonial Imperialism, Poverty, and Underdevelopment in Nigerian Pre-Independence Era	Colonial and Imperialism and Poverty and Underdevelopment and Nigeria and Pre-Independence
68	Street Mapping and House Numbering as a Tool for Effective National Security Transformation in Urban Kano	“Street Map” and “House Number” and Tool and “National Security” and Transformation and Urban and Effective and Kano
69	The Role of Religious Education in Promoting Peace Education for National Security and Transformation in Nigeria	Role and “Religious Education” and “National Security” and Transformation and Peace and Nigeria
70	Geographic Information System (GIS) as a Tool for Effective Crime Management in Nigeria	Geographic and Information and System and Tool and “Crime Management” and Effective and GIS and Nigeria

**Table 2:** Retrieved document identifiers for curated topics.

S/N	Topics	Retrieved document identifier
*English language*		
1	Assess and Role and Language and Communication and Transform and Sustain and National and Development and Nigeria	66, 44, 83, 23, 7, 20, 4, 26, 84, 37
2	Importance and Maintenance and Culture and Management and Growth and Organization and Nigeria	46, 89, 41, 7, 4, 28, 23, 62, 87, 97
3	Role and Islamic and Studies and Teacher and “Quality Control” and Education and Nigeria	18, 30, 19, 47, 83, 81, 34, 40, 39, 71
4	Leadership and Sport and Management and Play and Role and Development and Nigeria	67, 11, 68, 1, 30, 28, 42, 79, 77, 12
5	Science and Education and Good and Governance and Nigeria	65, 72, 11, 22, 23,31, 42,28, 59, 4
6	Islamic and Education and Panacea and Corruption and Nigeria	23, 27, 70, 72, 39, 67, 40, 71,81, 29
7	“Social Studies” and Education and “National Security” and Transformation and Agenda	65, 25, 67, 64, 4, 1, 31, 44, 20, 23, 30
8	Islamic and Education and Panacea and Corruption and Nigeria and Economy	28, 49, 23, 27, 70, 42, 71, 67, 81, 29
9	Transformation and Agenda and “Economic Development” and Nigeria	64, 65, 44, 80, 26, 41, 30, 23, 20, 31
10	Estimation and Percentage and Calcium and “Tap Water”	56, 19, 35, 89, 49, 37, 52, 41, 5, 33
11	Functions and “National Commission” and College and Education and NCCE and Teacher and Nigeria	3, 83, 40, 19, 85, 81, 57, 2, 71, 34
12	Socio-Cultural and Literacy and Education and “National Security” and Management and Transformation and Agenda and Nigeria	4, 27, 26, 25, 22, 31, 20, 30, 23, 44
13	“National Security” and “Human Right” and Protection and Social and Transformation	31, 25, 41, 21, 23, 44, 22, 30, 45, 20
14	Importance and Games and “Role Play” and Debate and Teaching and Technique and “Social Studies”	77, 79, 36, 65, 35, 25, 30, 69, 71, 47
15	Potential and “Theatre Arts” and Youth and National and Stability	4, 70, 66, 21, 3, 63, 52, 13, 26, 48
16	“Social Studies” and Education and Tool and Economy and Transformation and “Self-Reliance”	30, 81, 36, 31, 25, 80, 65, 64, 67, 49
17	Inter-Group and Multicultural and Society and Nigeria	55, 44, 58, 37, 22, 20, 49, 23, 26, 50
18	Women and Sport and Gender and Stereotype	68, 20, 40, 12, 38, 78, 1, 27, 15, 51
19	Youth and Empowerment and Kano and Conflict and National and Stability and Involvement and Remedy	36, 27, 65, 3, 37, 48, 23, 63, 13, 52
20	Challenge and Constitution and Reform and “Economic Depression” and National and Nigeria	84, 59, 85, 81, 67, 70, 53, 65, 64, 68
21	“Political Map” and Nigeria and 1960 and “National Rebirth”	65, 62, 67, 59, 68, 64, 57, 53, 21, 54
22	Geography and Education and “Natural Resources” and Management and “National Rebirth” and Nigeria	68, 65, 85, 74, 67, 62, 81, 57, 80, 56
23	“History Education” and “National Rebirth” and Effective	53, 72, 56, 64, 65, 59, 58, 62, 67, 57
24	Nigeria and “Historical Experience” and “National Rebirth” and Relevance	80, 67, 62, 56, 25, 59, 53, 64, 57, 58
25	Global and “Economic Depression” and Lesson and 1930 and Nigeria	65, 80, 35, 81, 66, 70, 68, 53, 64, 60
26	Attitude and “Value Reorientation” and Sine qua non and “National Rebirth” and Development	54, 65, 30, 56, 15, 67, 28, 62, 64, 61
27	“Political Education” and “National Security” and Nigeria and Rebirth	54, 44, 23, 58,, 57, 74, 59, 67, 62, 64
28	Youth and “Political Awareness” and Participation and Implication and “National Stability”	37, 3, 4, 15, 52, 48, 26, 13, 65, 63
29	“Social Studies” and Civic and Responsibility and Education and Instrument and “Economic Efficiency” and “National Rebirth”	72, 63, 49, 65, 86, 57, 90, 67, 62, 64
30	Constitution and Reform and “Economic Stability” and “National Rebirth” and Nigeria and “Social Studies” and Education	81, 85, 80, 68, 53, 59, 70, 67, 64, 65
31	Underdevelopment and “Economic Depression” and Nigeria and Transformation and Human and Natural and Resources	55, 65, 68, 64, 60, 56, 23, 14, 31, 66
32	Population and “Family Life” and Education and Constitution and Reform and “Economic Depression” and “National Rebirth”	81, 90, 67, 85, 53, 59, 70, 65, 64, 68
33	Menace and “Human Trafficking” and Nigeria and Implication and “Social Study” and Education	76, 80, 27, 65, 67, 22, 25, 30, 42, 69
34	Islamic and Constitutional and Reform and “Economic Depression” and “National Rebirth”	81, 85, 59, 67, 53, 68, 65, 64, 70, 90
35	Problem and New and Direction and Teaching and Learning and “Islamic Studies” and College and Education and National and Development	86, 29, 23, 39, 90, 57, 35, 74, 81, 71,
36	Church and Citizenship and leadership and Education and “National Rebirth”	12, 59, 62, 64, 68, 72, 67, 13, 57, 74
37	Parent and Child and Influence and Youth and Sport and Participation	15, 43, 42, 69, 26, 12, 51, 63, 36, 77
38	Role and “Physical Activity” and Treat and Prevent and Overweight and Obesity	30, 56, 90, 36, 28, 89, 69, 77, 83, 78
39	Information and Communication and Technology and Management and Administration and Sport and Tertiary and Institution	57, 37, 85, 30, 80, 77, 3, 28, 12, 79
40	Global and Economy and “Vision 2020” and Geography and “Melt Down” and Education	60, 4, 49, 56, 23, 70, 31, 53, 81, 80
41	Role and “Islamic Teaching” and Economy and Development and Nigeria	57, 65, 70, 29, 23, 72, 40, 81, 39, 71
42	Hitch and Organization and Administration and Guidance and Nigerian and “Secondary School” and Service	80, 57, 19, 31, 37, 36, 70, 85, 89, 82
43	Method and Activity and Effective and Teaching and Learning and Science and “Secondary School”	71, 57, 35, 84, 85, 25, 47, 49, 30, 83
44	Importance and “Mother Tongue” and Nigeria and Aspiration and Economy and Development	57, 49, 58, 65, 7, 15, 26, 36, 37, 84
45	“Secondary School” and Management and Constitution and Reform and “Economic Depression” and “National and Rebirth” and Nigeria	57, 56, 59, 67, 70, 68, 53, 65, 64, 85
46	Emerging and Role and UBE and Society and Implementation and “Universal Basic” and Education and Nigeria and “21st Century”	1, 34, 2, 81, 26, 85, 80, 4, 27, 86
47	Credible and “Electoral System” and Sustainable and Democracy and Nigeria and Constitution and Reform	68, 84, 64, 67, 15, 4, 59, 53, 87
48	Biography and Contribution and “Islamic Scholar” and Knowledge and “Muslim Society”	90, 29, 70, 39, 81, 72, 71, 40, 88, 18
49	Practicability and System and Nigeria and Transport and Industry	7, 58, 14, 28, 26, 84, 32, 48, 66, 89
50	Islamic and Solution and “Economic Depression” and Poverty	66, 71, 68, 18, 64, 60, 81, 29, 70, 90
51	Collaboration and Approach and Management and Women and Education and Sustainable and National and Development	62, 86, 12, 15, 27, 56, 28, 4, 85, 1
52	Appraisal and Part-Time and “Adult Education” and Programme and Geography	34, 55, 86, 81, 44, 80, 27, 4, 56, 2
53	Agriculture and Element and Facilitate and Growth and Stability and Nigeria and Economy	27, 53, 60, 65, 28, 32, 14, 13, 7, 3
54	“Adult Education” and Poverty and Sustainable and Economy and Development and Nigeria	86, 3, 37, 81, 14, 27, 60, 28, 1, 4
55	Examination and Analysis and Nigeria and “Economic Structure” and Organization and “Historical Development”	37, 23, 27, 19, 85, 86, 84, 57, 66, 7
56	Role and Arabic and Education and National and Stability and Nigeria and Pre-Independence	34, 68, 28, 13, 65, 23, 71, 48, 66, 63
57	HIV and AIDS and Awareness and Female and Student and Attitude and “Sexual Behavior”	85, 18, 49, 15, 13, 71, 42, 69, 27, 76
58	“Water Security” and Challenge and National and Transformation and Agenda and Nigeria	81, 22, 21, 26, 44, 23, 31, 30, 20, 41
59	Drug and Education and Leadership and Governance and Nigeria	62, 59, 65, 57, 27, 11, 68, 22, 28, 42
60	Role and Christian and Moral and Enforce and Women and Society	38, 69, 81, 1, 51, 74, 40, 27, 15, 43
61	Examination and Challenge and Governance and Nigeria and Economy	53, 31, 74, 23, 11, 42, 28, 65, 14, 19
62	“Citizenship Education” and Youth and Empowerment and National and Stability	74, 4, 26, 44, 63, 23, 3, 48, 52, 13
63	Contemporary and Education and Millennium and Enhance and Development and Goal and Islamic and Curriculum and Education	74, 4, 26, 44, 63, 23, 3, 48, 52, 13
64	Continuous and Assessment and Examination and Malpractice and Nigeria and Federal and College and Education	76, 57, 29, 83, 27, 23, 81, 74, 34, 19
65	“National Security” and Transformation and Agenda and Issues and Challenge and Nigeria	64, 21, 22, 44, 41, 26, 31, 30, 23, 20
66	“National Security” and Nigeria and Governance	31, 4, 59, 45, 20, 44, 23, 28, 22, 54
67	Colonial and Imperialism and Poverty and Underdevelopment and Nigeria and Pre-Independence	7, 4, 23, 87, 63, 58, 53, 54, 66, 14
68	“Street Map” and “House Number” and Tool and “National Security” and Transformation and Urban and Effective and Kano	67, 41, 20, 15, 30, 23, 25, 24, 54, 21
69	Role and “Religious Education” and “National Security” and Transformation and Peace and Nigeria	65, 26, 28, 20, 22, 25, 45, 44, 30, 23
70	Geographic and Information and System and Tool and “Crime Management” and Effective and GIS and Nigeria	81, 89, 67, 42, 80, 79, 57, 21, 56, 24

*Yoruba language*		
1	Ṣe àyẹ̀wò àti ipa àti èdè àti ìbánisọ̀rọ̀ àti ṣe àtúnṣe àti ìmúdúró àti ìdàgbàsókè àti Nàìjíríà	37, 41, 12, 53, 54, 65, 67, 70, 81, 84
2	Pàtàkì àti àmójútó àti àṣà àti ìṣàkóso àti ìdàgbàsókè àti àjọ àti Nàìjíríà	22, 25, 42, 11, 28, 46, 62, 65, 87, 4
3	Ipa àti ẹ̀sìn Íslam àti iwaduri àti olùkọ́ àti “iselekun ati isoro” àti ètò ẹ̀kọ́ àti Nàìjíríà	
4	Ṣíṣe olórí àti eré ìdárayá àti ìṣàkóso àti ìkópa àti ojúṣe àti ìdàgbàsókè àti Nàìjíríà	22, 42, 11, 12, 28, 51, 62, 63, 77, 79
5	Ìmọ̀ sáyẹ́nsì àti ètò ẹ̀kọ́ àti dáradára àti ìṣèjọba àti Nàìjíríà	22, 23, 42, 72, 4, 59, 31, 28, 11, 65
6	Ẹ̀sìn mùsùlùmí àti ẹ̀kọ́ àti ọ̀nà àbáyọ àti ìwà àjẹbánu àti Nàìjíríà	18, 23, 24, 39, 40, 29, 90, 72, 81, 70
7	“Ẹ̀kọ́ nípa àwùjọ” àti “ààbò orílẹ̀ èdè” àti ìmúpadàbọ̀sípò àti ìlànà	20, 21, 31, 22, 30, 23, 44, 45, 59, 25
8	Ẹ̀sìn Ísíláàmù àti ẹ̀kọ́ àti ọ̀nà àbáyọ àti ìwà àjẹbánu àti Nàìjíríà àti ọrọ́ ajé	23, 24, 39, 40, 42, 29, 31, 70, 81, 90
9	Ìmúpadàbọ̀sípò àti ìlànà àti ìdàgbàsókè ọrọ̀ ajé àti Nàìjíríà	14, 37, 29, 32, 44, 60, 66, 80, 81, 84
10	Ìsọtẹ́lẹ̀ àti petenti àti kásíọ̀mù àti omi ẹ̀rọ	14, 21, 37, 41, 33, 54, 56, 60, 79, 5
11	Àwọn ojúṣe àti àjọ tí ó n ṣe àmójútó àti Kọ́lẹ́ẹ̀jì àti ẹ̀kọ́ àti NCCE àti olùkọ́ àti Nàìjíríà	23, 34, 48, 57, 67, 71, 85, 3, 1, 2
12	Àṣà àwùjọ àti mọ̀ọ́kọ mọ̀ọ́kà àti ẹ̀kọ́ àti ààbò orílẹ̀ èdè àti ìṣàkóso àti ìmúpadàbọ̀sípò àti ìlànà àti Nàìjíríà	22, 23, 27, 41, 28, 30, 44, 63, 1, 4
13	Ààbò orílẹ̀ èdè àti ẹ̀tọ́ ọmọnìyàn àti ààbò àti àwùjọ àti ìmúpadàbọ̀sípò	20, 13, 23, 41, 22, 30, 44, 4, 45, 49
14	Pàtàkì àti eré ìdárayá àti “kíkópa” àti ìtàkúrọ̀sọ àti kíkọ́ni àti ẹ̀kọ́ àti ọgbọ́n àti “ẹ̀kọ́ nípa àwùjọ”	25, 23, 12, 47, 51, 65, 77, 79, 83, 86
15	Ìkójúòsùnwọ̀n àti “Ẹ̀kọ́ eré orí ìtàgé” àti ọ̀dọ́ àti orílẹ̀-èdè àti ìmúdúró	13, 26, 41, 42, 28, 48, 85,4, 1, 3
16	“Ẹ̀kọ́ nípa àwùjọ” àti ètò ẹ̀kọ́ àti ohun èlò àti ètò ọrọ̀ ajé àti ìmúpadàbọ̀sípò àti “ìdádúró ara ẹni”	41, 28, 31, 49, 58, 81, 85, 86, 3, 1
17	Ìbáṣepọ̀ àwọn ẹgbẹ́ àti àkójọpọ̀-àṣà àti àwùjọ àti Nàìjíríà	36, 23, 30, 46, 58, 62, 65, 84, 85, 87
18	Àwọn obìnrin àti eré ìdárayá àti ìyàtọ̀ takọtabo àti ìfojúàbùkù wò	15, 18, 27, 38, 12, 51, 77, 78, 79, 1
19	Ọ̀dọ́ àti ìrónilágbára àti ìlú Kano àti ìkọlura/èdè àìyedè àti orílẹ̀-èdè àti ìmúdúró àti kíkópa àti àtúnṣe	13, 21, 26, 37, 41, 52, 63, 65, 84, 1
20	Ìpèníjà àti òfin àti ìṣàtúnṣe àti “ètò ọrọ̀ ajé tó n rẹ̀yìn” àti orílẹ̀-èdè àti Nàìjíríà	20, 41, 43, 59, 64, 65, 67, 70, 84, 87
21	Máàpù ètò òṣèlú àti Nàìjéríà àti ọdún 1960 àti àtúnbí orílẹ̀-èdè	53, 21, 54, 62, 57, 59, 58, 67, 68, 64
22	Ìmọ̀ nípa agbègbè àti ẹ̀kọ́ àti “àwọn ohun àlùmọ́ọ́nì” àti “àwọn ohun àlùmọ́ọ́nì àti ìṣàkóso” àti àtúnbí orílẹ̀-èdè àti Nàìjíríà	72, 80, 81, 57, 56, 62, 58, 64, 59, 67
23	“Ẹ̀kọ́ nípa ìtàn” àti “àtúnbí orílẹ̀-èdè” àti àṣeyọrí	15, 19, 37, 11, 32, 64, 72, 78, 86, 87
24	Nàìjéríà àti “ìrírí nípa ìtàn” àti “àtúnbí orílẹ̀-èdè” àti ìjọra	14, 15, 23, 31, 53, 58, 64, 66, 67, 68
25	Káríayé àti ètò ọrọ̀ ajé tó n rẹ̀yìn àti ẹ̀kọ́ àti ọdún 1930 àti Nàìjíríà	23, 31, 53, 60, 65, 66, 80, 81, 7, 3
26	Ìhùwàsí àti “ìṣàtúnṣe ohun àkàkún-bàbàrà” àti “ọ̀nà àbáyọ” àti àtúnbí orílẹ̀-èdè àti ìdàgbàsókè	59, 61, 62, 64, 65, 67, 68, 70, 72, 90
27	Ẹ̀kọ́ nípa òṣèlù àti ààbò orílẹ̀-èdè àti Nàìjéríà àti àtúnbí	62, 59, 58, 23, 65, 64, 20, 74, 67, 57
28	Ọ̀dọ́ àti ìtanijí nípa òṣèlú àti kíkópa àti ìbámu àti ìmúdúró orílẹ̀-èdè	13, 26, 37, 30, 44, 49, 52, 62, 63, 4
29	“Ẹ̀kọ́ nípa àwùjọ” àti àwùjọ àti ojúṣe àti ẹ̀kọ́ àti ohun èlò àti “ètò ọrọ̀ ajé tó dára” àti àtúnbí orílẹ̀-èdè	57, 58, 61, 62, 64, 67, 70, 72, 74, 85
30	Òfin àti àtúnṣe àti “ìfẹṣẹ̀múlẹ̀ ètò ọrọ̀ ajé” àti àtúnbí orílẹ̀-èdè àti Nàìjéríà àti ẹ̀kọ́ nípa ìbáṣepọ̀ àwùjọ àti ìmọ̀ Ẹ̀kọ́	56, 57, 59, 64, 65, 67, 70, 72, 81, 84
31	Àìní ìdàgbàsókè àti “ọrọ̀ ajé tó n tòògbé” àti Nàìjéríà àti ìṣàtúnṣe àti ọmọ-ènìyàn àti àdámọ́ àti àlùmọ́ọ́nì	20, 43, 45, 57, 59, 64, 65, 70, 72, 29
32	Mímọ iye àti “ìgbé ayé ìdílé” àti ẹ̀kọ́ àti ofin àti ìṣàtúnṣe àti “ọrọ̀ ajé tí n tòògbé” àti àtúnbí orílẹ̀-èdè	22, 53, 57, 59, 64, 65, 67, 68, 70, 72
33	Ìṣòro àti “àṣà ìjínigbé-fiṣẹrú” àti Nàìjíríà àti àfàyọ/ipa àti “ẹ̀kọ́ nípa àwùjọ” àti ìmọ̀ ẹ̀kọ́	14, 25, 36, 42, 28, 60, 77, 81, 87, 7,
34	Ẹ̀sìn mùsùlùmí àti òfin àti ìṣàtúnṣe àti ètò “ọrọ̀ ajé tó dẹnu kọlẹ̀” àti àtúnbí orílẹ̀-èdè	53, 57, 59, 64, 65, 67, 70, 72, 81, 90
35	Ìṣòro àti titun àti ìdarí àti ìkọ́ni àti ìkẹ́kọ̀ọ́ àti “ẹ̀kọ́ ìmọ̀ ẹ̀sìn mùsùlùmí” àti ilé ẹ̀kọ́ gíga àti ẹ̀kọ́ àti orílẹ̀-èdè àti ìdàgbàsókè	13, 25, 26, 39, 40, 29, 34, 65, 72, 81
36	Ìjọ Ọlọ́run/sọ́ọ̀sì àti jíjẹ́ ọmọ orílẹ̀-èdè àti jíjẹ́ olórí àti ẹ̀kọ́ àti “àtúnbí orílẹ̀-èdè”	13, 57, 59, 61, 62, 64, 67, 68, 72, 74
37	Òbí àti ọmọ àti ipa àti ọ̀dọ́ àti eré ìdárayá àti kíkópa	26, 36, 43, 12, 51, 63, 69, 77, 84, 3
38	Ipa àti “àmúṣe àjẹmára” àti ìtọ́jú àti dídènà àti ìgbéwọ̀njù/ìwúwojù àti sísanrajù	15, 24, 27, 42, 33, 45, 53, 60, 69, 78
39	Àlàyé àti ìbánisọ̀rọ̀ àti ìmọ̀-ẹ̀rọ àti àmójútó àti ìṣàkóso àti eré ìdárayá àti ilé ẹ̀kọ́ gíga àti ìgbéklẹ̀	79, 77, 12, 80, 28, 37, 34, 81, 78, 44
40	Àgbáyé àti ọrọ̀ ajé àti àfojúsùn àti ọdún 2020 àti ẹ̀kọ́ nípa agbègbè àti yòòrò àti ẹ̀kọ́	80, 23, 27, 41, 42, 31, 34, 81, 1, 4
41	Ipa àti ẹ̀kọ́ ìmọ̀ ẹ̀sìn Ísíláàmù àti ètò ọrọ̀-ajé àti ìdàgbàsókè àti Nàìjíríà	23, 27, 40, 58, 60, 70, 72, 81, 90, 7
42	Ìṣòro àti àjọ àti ìṣàkóso àti ìtọ́ni àti èyí tó jẹ́ ti Nàìjíríà àti ilé-ẹ̀kọ́ girama àti ilé-ẹ̀kọ́ girama àti iṣẹ́	85, 82, 25, 36, 37, 12, 34, 86, 89, 1
43	Ọgbọ́n àti akitiyan àti àṣeyọrí àti kíkọ́ni àtî kíkọ́ ẹ̀kọ́ àti ìmọ̀ sáyẹ́nsì àti ilé ìwé girama	83, 86, 30, 84, 47, 57, 19, 81, 79, 34
44	Ìṣepàtàkì àti èdè abínibí àti Nàìjíríà àti àfojúsùn àti ètò ọrọ̀ ajé àti ìdàgbàsókè	64, 53, 70, 65, 81, 85, 60, 67, 68, 80
45	Ilé-ìwé girama àti ìṣàkóso àti òfin àti ìṣàtúnṣe àti ìrẹ̀wẹ̀sì ọrọ̀ ajé àti ọrọ̀-ajé àti àtúnbí àti Nàìjíríà	86, 80, 81, 4, 26, 34, 53, 27, 29, 84
46	Ìyọjú/Àṣẹ̀ṣẹ̀yọjú àti ipa àti UBE (Ẹ̀kọ́ alákọ̀ọ́bẹ̀rẹ̀ ẹsẹ̀kùkú) àti àwùjọ àti ìmúṣẹ àti “Ẹ̀kọ́ ìpìlẹ̀ káríayé” àti ètò ẹ̀kọ́ àti Nàìjíríà àti ọ̀rúndún kọkànlélógún	22, 87, 15, 53, 37, 57, 63, 65, 81, 4
47	Jíjẹ́ ìtẹ́wọ́gbà àti ìlànà ètò ìdìbò àti alágbèéró àti ìjọba àwa-ara-wa àti Nàìjíríà àti òfin àti àtúnṣe	70, 86, 71, 64, 37, 65, 27, 88, 43, 63
48	Ìtàn-ìgbésí-ayé àti ìlọ́wọ́sí àti “onímọ̀ nípa ẹ̀sìn Mùsùlùmí” àti ìmọ̀ àti “àwùjọ mùsùlùmí”	88, 70, 73, 27, 37, 43, 64, 65, 71,86
49	Ìṣàfihàn ìwúlò àti ìlànà àti Nàìjéríà àti ètò ìrìnnà àti ilé-iṣẹ́	66, 32, 58, 14, 30, 48, 59, 81, 84, 89
50	Ètò Ísíláàmù ọ̀nà àbáyọ àti “ọrọ̀ ajé tó dẹnukọlẹ̀” àti iṣẹ́	39, 29, 90, 89, 71, 72, 81, 89, 23, 40
51	Ìfọwọ́sowọ́pọ̀ àti ọ̀nà ìsúnmọ́ àti ìṣàkóso àti àwọn obìnrin àti ẹ̀kọ́ àti ìmúdúró àti orílẹ̀-èdè àti ìdàgbàsókè	1, 4, 22, 18, 27, 28, 59, 62, 65, 1, 70
52	Ìgbéléwọ̀n/àgbéyẹ̀wò àti apákan-àkókò àti “Ẹ̀kọ́ àgbà” àti ètò àti ẹ̀kọ́ nípa agbègbè	19, 56, 85, 13, 34, 47, 79, 80, 86, 3
53	Iṣẹ́ àgbẹ̀ àti èròjà àti ìmúdẹ̀rọ̀ àti ìdàgbàsókè àti ìmúdúró àti Nàìjéríà àti ọrọ̀ ajé	7, 3, 70, 4, 81, 60, 58, 32, 31, 27
54	“Ẹ̀kọ́ àgbà” àti iṣẹ́ àti ìmúdúró àti ètò ọrọ̀ ajé àti ìdàgbàsókè àti Nàìjéríà	4, 1, 13, 28, 54, 58, 60, 67, 70, 7
55	Àgbéyẹ̀wò àti ìtúpalẹ̀ àti Nàìjéríà àti “ìgbékẹ̀lé ètò ọrọ̀ ajé” àti àjọ àti “ìdàgbàsókè ajẹmọ́-ìtàn”	24, 39, 12, 33, 57, 69, 85, 86, 89, 1
56	Ipa àti Lárúbáwá àti Ẹ̀kọ́ àti orílẹ̀-èdè àti ìmúdúró àti Nàìjéríà àti sáájú òmìnira	25, 42, 54, 58, 63, 66, 68, 87, 1, 4
57	Àìsàn HIV àti Éèdì àti ìmọ̀ àti obìnrin àti akẹ́kọ̀ọ́ àti ìṣesí àti “ìbálòpọ̀”	76, 71, 69, 51, 47, 42, 40, 36, 27, 15
58	“Ìpèṣè omi” àti ìpèníjà àti orílẹ̀-èdè àti ìṣàtúnṣe àti ìlànà àti Nàìjéríà	41, 29, 32, 59, 65, 67, 74, 81, 84, 87
59	Oògùn àti ètò ẹ̀kọ́ àti ìdarí àti ìṣàkóso àti Nàìjéríà	11, 42, 28, 23, 22, 57, 62, 67, 79, 85
60	Ipa àti Kìrìsítẹ́nì àti ìwà àti ìfagbáramú àti obìnrin àti àwùjọ	27, 15, 43, 13, 36, 69, 40, 51, 1, 4
61	Àyẹ̀wò àti ìpèníjà àti ìṣàkóso àti ìpèníjà àti Nàìjéríà àti ọrọ̀ ajé	
62	“Ẹ̀kọ́ jíjẹ́ ọmọ orílẹ̀-èdè” àti ọ̀dọ́ àti ìrónilágbára àti orílẹ̀-èdè àti ìmúdúró	13, 4, 1, 74, 69, 68, 63, 52, 26, 22
63	Ìbáyému àti ẹ̀kọ́ àti ẹgbẹ̀rún àti ìmúdára àti ìdàgbàsókè àti àfojúsùn àti ìjẹmọ́-ẹ̀sìn mùsùlùmí àti kòríkúlóòmù àti ètò ẹ̀kọ́	18, 71, 29, 23, 26, 39, 40, 34, 49, 72
64	Àtìgbàdégbà àti ìgbéléwọ̀n àti ìdánwò àti àìṣedéédé àti Nàìjéríà àti ìjọba àpapọ̀ àti ilé ẹ̀kó gígs àti ètò ẹ̀kọ́	19, 85, 42, 32, 34, 56, 62,80, 81, 86
65	Ètò ààbò orílẹ̀-èdè àti ìṣàtúnṣe àti ìlànà àti àwọn àríyànjiyàn àti ìpèníjà àti Nàìjéríà	41, 20, 22, 23, 30, 44, 59, 65, 67, 70
66	“Ètò ààbò orílẹ̀-èdè” àti Nàìjéríà àti ìṣèjọba	28, 22, 42, 11, 23, 31, 54, 59, 65, 68
67	Amúnisìn àti ìjẹgàba àti iṣẹ́ àti àìsí-ìdàgbàsókè àti Nàìjéríà àti sáájú òmìnira	14, 23, 48, 53, 54, 57, 65, 58, 66, 68
68	Máàpù òpópónà' àti 'kíkọ nọ́mbà sára ilé' àti ohun èlò àti ààbò orílẹ̀ èdè àti ìṣàtúnṣe àti ìlú nlá àti àṣeyọrí àti Kano	21,, 24, 11, 30, 52, 67, 53, 88, 54,58
69	Ipa àti 'ẹ̀kọ́ nípa ẹ̀sìn' àti ààbò orílẹ̀-èdè àti ìṣàtúnṣe àti àlàáfíà àti Nàìjéríà	23, 30, 22, 44, 25, 59, 64, 69, 67, 70
70	Ìmọ̀ nípa agbègbè àti àlàyé àti ìlànà àti ohun èlò àti 'mímójútó ìwà ọ̀daràn' àti àṣeyọrí àti àlàyé nípa agbègbè àti Nàìjéríà	24, 15, 21, 12, 32, 34, 49, 58, 84, 1

*Igbo language*		
1	Ileba anya n'ọrụ asụsụ na Nzikọrịta ozi na-arụ n'igbanwe nakwa n'ikwado mmepe obodo	66, 20, 84, 13, 26, 4, 74, 22, 45, 37
2	Uru dị n'imezi Omenaala n'ebe ihe banyere njikwa na uto nke otu Ọgbakọ dị iche iche dị na Nigeria	46, 89, 23, 28, 85, 7, 87, 29, 41, 42
3	Ọrụ dịịrị ndị nkuzi ihe Ọmụmụ Sayensi n'ijikwa agụmakwụkwọ na Nigeria	83, 39, 28, 23, 25, 34, 80, 40, 81, 37
4	Ndị ndu na-ahazi egwuregwu nakwa Ọrụ pụrụ iche ha na-arụ n'ikwalite egwuregwu dị iche iche na Nigeria	47, 43, 69, 23, 1, 51, 42, 12, 30, 79
5	Ihe Ọmụmụ sayensi na ezigbo ọchịchị na Nigeria	28, 42, 65, 67, 22, 11, 23, 69, 27, 30
6	Agụmaakwụkwọ Islam dị ka ihe a ga-eji gwọta ihe nrụrụ aka na Nigeria	29, 81, 72, 90, 39, 67, 70, 23,40, 71
7	Agụmaakwụkwọ banyere mmekọrịta ọha na eze nakwa usoro mgbanwe nchekwa obodo	59, 67, 64, 41, 65, 22, 30, 23, 50, 20
8	Agụmaakwụkwọ Islam dị ka ihe a ga-eji gwọta ihe nrụrụ aka n'akụanụba Nigeria	21, 81, 90, 71, 72, 67, 70, 65, 23, 28
9	Usoro mgbanwe: Isi sekpụ nti n'ọrụ mmepe akụnaụba na Nigeria	33, 41, 5, 52, 37, 81, 89, 80, 49, 35
10	Atụmaatụ nke pasenti kalshium n'ụfọdụ mmiri mgbata	34, 71, 39, 25, 57, 2, 3, 83, 37, 81
11	Ọrụ dịịrị ndị otu komishonu ụlọakwụkwọ ọzụzụ ndi nkuzi (NCCE) n'inye ndi nkuzi ezigbo ọzụzụ na Nigeria	41, 23, 7, 85, 59, 62, 37, 22, 46, 31
12	Mmụta banyere Omenaala na mmekọrịta Ọhanaeze dị ka mmalite maka njikwa ọrụ nchekwa obodo nakwa atụmatụ mgbanwe obodo na Nigeria	45, 22, 23, 44, 20, 18, 4, 30, 21, 13
13	Nchekwa obodo, nchekwa ikike mmadụ nakwa mgbanwe nyere mmekọrịta mmadụ na ibe ya	47, 36, 30, 65, 86, 69, 26, 83, 67, 45
14	Uru dị n'egwuregwu, ịbụ onye ọzọ n'egwuregwu na usoro nkuzi arụmarụụka bara n'ọmụmụ banyere mmekọrịta mmadụ na ibe ya	26, 36, 30, 45, 47, 65, 67, 69, 71, 86
15	Ikike ndi ụlọ na-eme ihe nkiri na-enye ndi ntorobia maka nkwudosiike nke obodo	13, 22, 26, 28, 45, 63, 69, 85, 3, 4
16	Mmụta banyere mmekọrịta Ọhanaeze dị ka ngwa ọrụ maka mgbanwe akụnaụba na ndabere onwe	49, 64, 67, 68, 80, 81, 85, 1, 4, 7
17	Usoro mmekọrịta dị n'etiti ọha mmadụ di iche iche na omenaala dị iche iche dị ka na Nigeria	23, 63, 50, 46, 48, 27, 25, 58, 66, 42
18	Ụmụnwaanyị n'egwuregwu: Echiche mmadụ banyere okike n'oge gara age nakwa n'oge ugbu a	51, 83, 78, 26, 27, 77, 79, 12, 76, 37
19	Ikike ndi ntorobịa: Dị ka ihe a ga-eji kwụsị ndi ntorobịa itinye aka n'esemokwu dị n'obodo Kano maka nkwudosike nke Obodo	55, 42, 85, 52, 76, 26, 13, 63, 35, 69
20	Ihe imaaka nyere mgbanwe usoro iwu, Ịda mba n'akụnaụba nakwa ọchịchọ nke Nigeria maka mmugharị obodo	31, 67, 58, 30, 65, 4, 23, 53, 64, 87
21	Mgbanwe maapụ ndọrọndọrọ ọchịchị na Nigeria site n'afọ 1960 ruo oge ugbu a nakwa mmụgharị nke mba	54, 53, 64, 20, 67, 34, 58, 57, 68, 21,
22	Ihe ọmụmụ giografi na njikwa akarangwa dị iche iche sitere n'ihe okike maka mmughrị nke obodo Nigeria	28, 62, 44, 85, 60, 20, 31, 68, 41, 3
23	Ihe ọmụmụ histri dị ire maka mmugharị nke obodo	58, 59, 2, 25, 75, 12, 73, 14, 57, 62
24	Mkpa akụkọ ihe merela eme dị maka mmugharị ọzọ nke Nigeria	67, 66, 53, 58, 14, 59, 86, 45, 15, 25
25	Ndakpọ akụnaụba mba ụwa site n'afọ 1930 nakwa ihe nkuzi e nwetara na ya na Nigeria	60, 4, 81, 23, 65, 85, 3, 80, 14, 28
26	Ntụgharị echiche na uru e nwetara: Otu ụzọ dị oke mkpa a ga-eji mugharịa nakwa mepee obodo	30, 18, 37, 53, 36, 28, 23, 4, 47, 44
27	Itinyegharị uche n'agụmaakwụkwọ banyere ndọrọndọrọ ọchịchị na nchekwa obodo maka mmugharị nke Nigeria	62, 23, 44, 22, 30, 20, 4, 66, 13, 25
28	Ebe ndi ntorobia kwụ n'ịmata nakwa n'itinye aka na ndọrọndọrọ ọchịchị: Ihe ọ pụtara na nkwudosiike nke obodo	63, 13, 52, 26, 65, 3, 62, 80, 81, 37
29	Ihe ọmụmụ na-ahụ maka mmekọrịta mmadụ na ibe ya dị ka ngwa ọrụ maka ọrụ obodo, arụmọrụ akụnaụba nakwa mmụgharị nke mba	25, 49, 57, 58, 62, 64, 65, 71, 69, 67
30	Inweta mgbanwe n'usoro iwu maka nkwusi ike nke akụnaụba na mmugharị nke obodo Nigeria site n'iso ụzọ ihe ọmụmụ na-ahụ maka mmekọrịta mmadụ na ibe ya	44, 49, 62, 64, 65, 67, 68, 85, 86, 87
31	Enweghị ezi mmepe na ịda mba Akụnaụba na Nigeria: mgbanwe mmadụ nakwa akarangwa di iche iche sitere n'ihe okike na Nigeria	56, 60, 7, 4, 81, 68, 66, 53, 49, 29
32	Ọnụọgụgụ ndi mmadụ, nkuzi banyere obibi ndụ na ezinaụlọ, idozighari usoro iwu, Ndakpọ akụnaụba nakwa mmunwe Nigeria	68, 84, 64, 22, 25, 65, 27, 67, 70, 81
33	Ihe egwu di n'azụmahịa mgbere mmadụ na Nigeria:Uru ọ bara ịmụ ihe ọmụmụ na-ahụ maka mmekọrịta mmadụ na ibe ya	44, 42, 66, 72, 64, 49, 59, 60, 27, 23
34	Etu Islam siri hụta idozighari usoro iwu, Ndakpọ akụnaụba nakwa mmụgharị Nigeria	64, 68, 13, 27, 38, 53, 65, 70, 81, 85
35	Nsogbu dịịrị nkuzi na mmụta ihe ọmụmụ Islam n'ụlọakwụkwọ ọzụzụ ndi nkuzi Usoro ọhụrụ maka ọrụ mmepe obodo	71, 12, 13, 1, 68, 65, 62, 57, 54, 43
36	Ụka na ndi nwe obodo nakwa ihe ọmụmụ banyere ndi ndu maka mmụghari nke obodo	45, 43, 22, 20, 23, 25, 30, 60, 75, 87
37	Inye mkpebi nke ndi nne na nna dị ka o si metuta nwantakịrị n'oge ha di na ntorobịa nakwa n'ogo isonye n'egwuregwu	15, 24, 35, 42, 43, 33, 52, 55, 69, 78
38	Ọrụ dị na mmegharị ahụ dị ka o si metuta igbochi na ịgwọ ọrịa oke ibu	79, 77, 83, 28, 85, 78, 37, 34, 57, 12
39	Nkwukọrịta na nzikọrịta ozi n'usoro tekụnụzụ n'ijikwa na ịchịkwa egwuregwu n'ụlọakwụkwọ dị elu	21, 37, 11, 12, 28, 51, 66, 77, 79, 83
40	Ndakpọ akụnaụba mba ụwa na vishonu 2020 nakwa amụmamụ giografi	22, 23, 24, 31, 53, 56, 60, 67, 80, 81
41	Ọrụ dịịrị nkuzi Islam na mmepe akụnaụba na Nigeria	18, 23, 29, 39, 40, 7, 71, 72, 81, 90
42	Ihe mgbochi dị na nhazi na ịchịkwa ọrụ nduzi di iche iche dị n'ụlọakwụkwọ sekọndịrị na Nigeria	83, 25, 77, 85, 30, 35, 71, 28, 79, 34
43	Usoro dabere na mmegharị aka maka nkuzi na mmụta sayensi tọrọ atọ n'ụlọakwụkwọ sekọndịrị	18, 35, 30, 34, 47, 49, 57, 71, 83, 85
44	Uru asụsụ nne nwa bara n'ebe Ihe banyere ọchịchọ Nigeria n'ebe ọrụ mmepe akụnaụba dị	65, 53, 11, 70, 85, 22, 25, 59, 67, 81
45	Ileba anya na nhazi ụlọakwụkwọ sekọndịrị, idozighari usoro iwu, ndakpọ akụnaụba nakwa mmugharị Nigeria	13, 26, 27, 30, 34, 80, 2, 81, 86, 4
46	Ọrụ ndi pụtara ihe n'etiti oha na eze n'imejuputa ọnọdụ agụmaakwụkwọ (UBE) na Nigeria na 21 senchuri	26, 27, 34, 51, 59, 81, 85, 86, 4, 87
47	Ọchịchọ maka ezi usoro ntuli aka kwesịrị ntụkwasa obi banyere ndigide ọchịchị onye kwuo uche ya na Nigeria: Mkpa ọ dị idozigharị iwu	22, 53, 59, 62, 64, 63, 70, 65, 67, 87
48	Akụkọ gbasara ndụ na ihe nweta sitere n'aka ndi ọkammụta Islam n'ihe ọmụmụ metutara ọgụgụ isi nke ndi otu muslim	18, 38, 39, 71, 81, 40, 72, 88, 90, 70
49	Imepụta usoro ịkwụ ụgwọ nke gbadoro ụkwụ n'ọrụ n'ụlọ ọrụ ndi na-ahụ maka njem	21, 25,26, 48, 66, 70, 77, 81, 84, 89
50	Islam, dị ka usoro a ga-eji gwọọ ndakpọ akụnaụba nakwa ida ogbenye	14, 18, 22, 54, 23, 60, 4, 29, 70, 90
51	Ụzọ imekọ ihe ọnụ maka njikwa agụmaakwụkwọ ụmụ nwaanyị maka mmepe mba. na-adigide adigide	15, 27, 37, 56, 80, 3, 4, 87, 1, 81, 4
52	Itule profaịlụ nke mmemme agụmaakwụkwọ nke ọkara oge ndi okenye na giografi	19, 34, 44, 49, 55, 80, 56, 81, 2, 4
53	Agrikolcho: Dị ka isi ihe dị oke mkpa n'ikwado uto na nkwudosi ike nke akụnaụba Nigeria	37, 31, 32, 70, 64, 56, 3, 4, 7, 53
54	Agụmaakwụkwọ ndi okenye na ibelata ida ogbenye dị ka ụzọ a ga-eji nwee mmepe akụnaụba na-adigide	15, 18, 37, 28, 31, 55, 81, 90, 1, 4
55	Ime nyocha n' usoro akụnaụba Nigeria na nhazi mmepe akụkọ ihe megoro eme	14, 20, 45, 57, 58, 65, 66, 80, 81, 7
56	Inyocha ọrụ dị mkpa dị ka o si metuta agụmaakwụkwọ Arab maka nkwudosike nke mba tupu Nigeria e nwere onwe ha	13, 23, 36, 58, 63, 66, 64, 65, 71, 81
57	Ime ka a mata ọrịa HIV na AIDS nakwa agwa ụmụakwụkwọ ndi nwaanyị gbasara mmekọ nwoke na nwaanyị	18, 19, 27, 42, 47, 51, 62, 69, 1, 76
58	Nsogbu ịchekwa mmiri na atụmatụ mgbanwe mba n'ala Nigeria	41, 56, 59, 64, 70, 65, 67, 75, 81, 87
59	Mkpa ọ dị inwe mmụta banyere ajọ ọgwụ maọbụ ndị ndu na ezigbo ọchịchị na Nigeria	22, 23, 42, 26, 11, 28, 46, 54, 61, 62
60	Ọrụ dịịrị ụmụnwaanyị ndi ụka Kraịst n'ikwado ụkpụrụ omume n'ime obodo	15, 23, 27, 40, 43, 51, 62, 64, 74, 1
61	Inyocha ihe ima aka dị oke mkpa na-echere ezigbo ọchịchị na akụnaụba Nigeria aka mgba	22, 59, 23, 42, 28, 31, 11, 63, 65, 4
62	Ọgụgụ akwụkwọ nwa amaala nakwa ọrụ ndi ntorobịa maka nkwudosike nke mba	13, 26, 37, 48, 52, 63, 65, 68, 1, 4
63	Okwu ndi a kpụ n'ọnụ ugbu a na agụmaakwụkwọ: Ikwalite ebumnuche mmepe nke milenium site n'usoro ọmụmụ nke agụmaakwụkwọ Islam	18, 20, 35, 36, 40, 29, 49, 62, 71, 81
64	Ntule na-aga n'ihu nakwa ime mpụ n'ule n'ụlọ akwụkwọ kọleji ọzụzụ ndi nkuzi	19, 34, 47, 57, 71, 72, 79, 80, 81, 83
65	Nchekwa obodo na usoro mgbanwe na Nigeria: okwu a kpụ n'ọnụ na ihe na-echere ya aka mgba	20, 22, 23, 27, 41, 30, 44, 45, 59, 65
66	Ikwalite nchekwa obodo site n'ezi ọchịchị	13, 22, 23, 11, 28, 31, 44, 45, 59, 4
67	Oge ọchịchị ndi ọcha, ịda ogbenye nakwa enweghi mmepe na Nigeria tupu oge nnwere onwe	22, 28, 54, 58, 59, 66, 81, 87, 4, 7
68	Ikewa okporo ama dị iche iche na inye ọnụọgụgụ n'ụlọ dị ka ngwa ọrụ maka mgbanwe nchekwa obodo di ire n'ebe mepere emepe na Kano	21, 24, 41, 42, 54, 58, 67, 69, 88, 90
69	Ọrụ dịịrị ihe ọmụmụ banyere okpukperechi n'ịkwalite ihe ọmụmụ banyere udo maka nchekwa obodo na mgbanwe na Nigeria	20, 22, 23, 25, 26, 41, 30, 44, 45, 65
70	Usoro nzikọrịta ozi giografi (GIF) dị ka ngwa ọrụ maka njikwa mpu dị ire na Nigeria	21, 24, 11, 34, 56, 57, 79, 80, 81, 89

*Hausa language*		
1	Aunawa da matsayi da harshe da sadarwa da sauyi da	13, 26, 27, 37, 44, 58, 65, 84, 85, 4
2	Muhimmanci da gyare gyare and al'ada da shugabanci da ci gaban da ma'aikata da Nijeriya	23, 27, 37, 28, 46, 61, 62, 87, 1, 7
3	Matsayin da addinin musulunci da nazari da Malamai da tabbatar da nagarta da ilmi da Nijeriya	18, 23, 27, 38, 39, 40, 59, 71, 72, 81
4	Tsarin shugabanci da wasanni da jagoranci da rawa da takawa ci-gaba da Nijeriya	7, 79, 77, 51, 28, 12, 11, 42, 26, 13
5	Ilmin kimiyya da kyakkyawan shugabanci da Nijeriya	22, 23, 42, 11, 28, 31, 54, 59, 68, 83
6	Ilmin da addinin musulunci da mafita da cin-hanci da Nijeriya	23, 39, 40, 29, 49, 59, 70, 71, 72, 81
7	Nazarin walwala da ilmi da tsaron kasa da kawo sauyi da tsari	4, 67, 64, 59, 30, 27, 25, 22, 20, 13
8	Addinin musulunci da ilmi da mafita da cin-hanci da Nijeriya da tattalin arziki	81, 72, 71, 65, 59, 29, 28, 40, 39, 23
9	Kawo sauyi da tsari da ci-gaban tattalin arziki da Nijeriya	15, 19, 23, 35, 33, 52, 59, 89, 2, 3
10	Kididigar da kaso cikin dari da sinadarin kalsiyam da “ruwan famfo”	3, 2, 89, 59, 52, 33, 35, 23, 19, 15
11	Ayyukan da hukumar kula da kwalejoji da ilmi da NCCE da ilmin koyarwa da Nijeriya	23, 34, 48, 57, 67, 71, 81, 85, 2, 4
12	Al'adu-zamantakewa da ilmin da “tsaron kasa” da gudanarwa da kawo sauyi da tsari da Nijeriya	87, 85, 67, 65, 46, 44, 30, 25, 23, 22
13	“tsaron kasa” da “hakkin dan Adam” da kare da walawala da sauyi	20, 21, 22, 23, 25, 30, 27, 44, 54, 4
14	Muhimmancin da wasanni da “kwaikwayo” da muhawara da koyarwa da dabarun da ‘nazarin walwala”	35, 36, 30, 47, 49, 64, 65, 69, 74,83
15	Rawar da ilmin wasannin kwaikwayo da matasa da zanan lafiyar kasa	26, 13, 22, 37, 28, 52, 63, 78, 3, 4
16	Nazarin walwala da ilmin da hanya da tattalin arziki da sauyi “dogaro da kai”	3, 81, 80, 74, 67, 64, 49, 31, 30, 27
17	Dangantaka tsakanin kabilu da kasa mai dauke da kabilu da yawa da al'umma da Nijeriya	64, 59, 54, 53, 50, 49, 44, 22, 14, 13
18	Mata da wasanni da jinsi	1, 79, 78, 77, 51, 12, 40, 38, 27, 14
19	Matasa da tallafin sana'a da Kano da rikici da na kasa da kwanciyar hankali	26, 13, 23, 37, 28, 48, 52, 57, 63, 3
20	Matsaloli da tsarin mulki da gyara da “masassarar tattalin arziki” da na kasa da Nijeriya	28, 59, 64, 65, 67, 68, 80, 81, 87, 7
21	“taswirar siyasar” da Nijeriya da 1960 zuwa yau da “Nijeriya sabuwa”	87, 74, 69, 68, 66, 58, 57, 54, 53, 23
22	Nazarin bayaynin kasa da ilmin da “albarkatun kasa” da sarrafa da na kasa da “Nijeriya sabuwar”	11, 28, 34, 44, 56, 57, 67, 80, 85, 1
23	“ilmin tarihi” da Nijeriya sabuwa da	14, 20, 57, 58, 59, 60, 65, 66, 88, 7
24	Nijeriya da “faruwar abubuwa tarihi” da “Nijeriya sabuwa”	20, 23, 58, 65, 66, 68, 74, 69, 85, 7
25	Na duniya da “masassarar tattalin arziki” da kalubale da 1930 da Nijeriya	65, 59, 58, 53, 44, 28, 11, 41, 27, 23
26	Halayyar da “gangamin sauya halayya” da tafarkin “Nijeriya sabuwa da ci gaba	
27	“ilmin siyasa da “tsaron kasa” da Nijeriya sabuwa	
28	Matasa da “wayewar siyasa” da shiga a dama da sakamakon da “kwanciyar hankalin kasa”	51, 52, 63, 2, 3, 4, 13, 26, 28, 48
29	“nazarin walwala” da na dan kasa da hakkoki da ilmi da hanyar da “habbakar tattalin arziki” da Nijeriya sabuwa	23, 26, 44, 45, 49, 58, 63, 81, 84, 4
30	Tsarin mulki da gyara da “habakar tattalin arziki” da “Nijeriya sabuwa” Nijeriya da “nazarin walawala” da ilmi	22, 23, 42, 28, 59, 64, 65, 67, 84, 4
31	Rashin ci gaba da “masassarar tattalin arziki” da Nijeriya da sauyi da dan Adam da albarkatun da kasa	14, 31, 49, 56, 57, 58, 59, 66, 4, 7
32	Yawan al'umma da “tsarin iyali” da ilmi da tsarin mulki da gyara da “masassarar tattalin arziki” da Nijeriya sabuwa”	11, 23, 28, 59, 65, 67, 84, 68, 87, 4
33	Yawaitar da “safarar mutane zuwa ketare” da Nijeriya da kalubale da “nazarin walwala” da ilmi	26, 20, 23, 44, 28, 57, 63, 84, 65, 4
34	Musulunci da tsarin mulki da gyara da “masassarar tattalin arziki” da Nijeriya sabuwa	70, 68, 67, 65, 22, 23, 40, 41, 59, 38
35	Matsalolin da sabuwar hanyar da koyarwa da koyo da “ilmin addinin musulunci” da kwalejojin da ilmi da Nijeriya da ci gaba	23, 35, 34, 49, 57, 65, 67, 71, 83, 85
36	Choci da zama dan kasa da shugabanci da ilmi da “Nijeriya sabuwa”	74, 68, 57, 46, 28, 12, 42, 23, 13, 22
37	Iyaye da yara da tasiri da matasa da wasanni da shiga a dama	26, 36, 42, 12, 51, 52, 63, 77, 78, 86
38	Rawar da “aikin motsa jiki” da magance da karewa da nauyi da yawa kiba	21, 22, 26, 11, 45, 48, 51, 78, 87, 5
39	Hanyar sadarwa da ta zamani da gudanarwa da shugabanci da wasanni da manyan da makarantu	79, 77, 57, 66, 51, 28, 12 11, 21, 13
40	Na duniya tattalin arziki da “burin 2020” da nazarin bayanin kasa da “durkushewa” da ilmi	23, 37, 28, 31, 56, 57, 58, 64, 65, 84
41	Rawar da “koyar da ilmin addinin musulunci” da tattalin arziki da ci gaba da Nijeriya	14, 23, 37, 28, 59, 64, 65, 66, 3, 7
42	Matsaloli da gudanarwa da shugabacin da jagoranci da makarantun sakandire	25, 42, 12, 28, 34, 57, 77, 82, 85, 89
43	Dabarar koyarwa da ta shafi yi da kai da aiki da koyarwa da koyo da kimiyya da “makarantun sakandire	84, 83, 71, 57, 49, 30, 35, 26, 25
44	Muhimmancin da “harshen uwa” da Nijeriya da buri da tattalin arziki da ci gaban	37, 28, 31, 53, 57, 60, 64, 65, 84, 4
45	“makarantun sakandire” da shugabanci da tsarin mulki da gyaran da “masassarar tattalin arziki” da Nijeriya sabuwa da Nijeriya	82, 81, 68, 67, 65, 59, 31, 11, 42, 25
46	Sababbin da rawan da UBE da al'umma da aiwatarwa da “ilmi na bai daya” da ilmi da Nijeriya da “karni na 21”	24, 26, 27, 34, 49, 59, 80, 84, 86, 4
47	Na gaskiya da “tsarin zabe” da mai dorewa da Damokaradiyya da Nijeriya da tsarin mulki da gyara	15, 22, 42, 11, 28, 53, 59, 65, 67, 87
48	Tarihin rayuwa da gudunmawar da “malamin addinin musulunci” da ilmi da “al'ummar musulmi”	37, 57, 59, 65, 71, 73, 81, 86, 88, 1
49	Yiwuwar aiwatar da tsarin nijeriya da safuri da masana'antu	28, 32, 49, 58, 66, 77, 86, 87, 89, 3
50	Musulunci da hanyar warware da “masassarar tattalin arziki” da talauci	18, 23, 27, 38, 29, 70, 72, 81, 90, 4
51	Hadin guiwa da salon da gudanarwa da mata da ilmi da mai dorewa da na kasa da ci gaba	4, 1, 86, 85, 56, 28, 12, 40, 27, 15
52	Nazarin da na wucin gadi da “ilmin manya” da nazarin bayanin kasa	27, 40, 30, 34, 56, 80, 81, 87, 2, 4
53	Noma da sinadarin taimakawa da karuwa da daidaito da Nijeriya da tattalin arziki	20, 45, 57, 58, 60, 66, 68, 3, 4, 7
54	“ilmin manya” da talauci da mai dorewa da tattalin arziki da ci gaban Nijeriya	14, 27, 37, 28, 60, 81, 86, 87, 1, 4
55	Awo da nazari da Nijeriya da “tsarin tattalin arziki” da gudanarwa da”tarihin ginuwarasa”	24, 12, 28, 56, 57, 60, 63, 85, 89, 1
56	Rawar da takawa” da larabci da ilmi da kwanciyar hankalin kasa da Nijeriya kafin ‘yancin kai	
57	Cutar HIV da kanjamau da masaniya da mata da dalibai da halayya da “jima'i”	76, 85, 69, 55, 47, 12, 42, 40, 27, 15
58	“wadatuwar ruwan sha” da kalubale da na kasa da sauyi da buri da Nijeriya	20, 28, 30, 31, 58, 59, 61, 65, 70, 4
59	Miyagun kwayoyi da ilmintarwa da jagoranci da shugabanci da Nijeriya	22, 27, 42, 11, 28, 57, 59, 62, 65, 68
60	Rawar da kirasta da da'a da tabbatar da da mata da al'umma	1, 78, 69, 51, 43, 40, 38, 27, 20, 15
61	Auna da kalubale da shugabanci da Nijeriya da tattalin arziki	25, 27, 42, 11, 12, 28, 53, 57, 59, 7
62	“ilmin dan kasa nagari” da matasa da tallafawa da sana'a da kwanciyar hankalin kasa	37, 28, 13, 26, 34, 48, 52, 63, 65, 3
63	Sababbin batutuwa da ilmi da sabon karni da taimakawa da ci gaba da manufa da musulunci da manhaja da ilmi	18, 23, 24, 26, 40, 29, 47, 71, 84, 86
64	Gwaji da na yau da kullun da jarrabawa da magudin jarrabawa da Nijeriya da kwalejin da ilmi da tarayya	19, 23, 24, 34, 48, 58, 67, 71, 79, 82
65	“tsaron kasa” da sauyi buri da batutuwa da kalubale da Nijeriya	20, 22, 23, 41, 26, 44, 25, 87, 4, 27
66	“tsaron kasa” da Nijeriya da shugabanci	45, 30, 44, 13, 20, 12, 27, 25, 23, 22
67	Mulkin mallaka da talauci rashin ci gaba da Nijeriya da kafin “yancin kai”	14, 23, 32, 53, 57, 58, 66, 80, 81, 4
68	“Taswirar layuka” da “lambar gidaje” hanya ce da “tsaron kasa” da sauyi da birane da mai karfi da Kano	21, 30, 22, 44, 23, 20, 52, 25, 45, 26
69	Rawar da “ilmin addini” da “tsaron kasa” da sauyi da zaman lafiya da Nijeriya	4, 45, 44, 30, 27, 26, 25, 23, 22, 20
70	Tsarin bayanin taswirar kasa da hanya ce da “sarrafa laifuka´da GIS da Nijeriya”	21, 24, 26, 37, 34, 45, 53, 54, 56, 65

**Table 3:** LAIRS evaluation results.

S/N	Document retrieved (DR)	Relevant document (RD)	No. of RDR@10	No. of RD	Precision @10	Recall @10	F-measure @10
*English language*							
1	66, 44, 83, 23, 7, 20, 4, 26, 84, 37,	8, 46, 37, 41, 44, 20, 22	3	7	0.30	0.43	0.35
2	46, 56, 41, 7, 4, 28, 23, 62, 87, 79	85, 12, 46, 37, 39, 8	1	6	0.10	0.17	0.13
3	18, 30, 19, 47, 83, 81, 34, 40, 39, 71	81, 34, 72, 38, 40, 37	3	6	0.30	0.50	0.38
4	67, 11, 68, 1, 30, 28, 42, 79, 77, 12	51, 11, 12, 43	2	4	0.20	0.50	0.29
5	65, 72, 11, 22, 23, 31, 42, 28, 59, 4	83, 11, 28, 37	2	4	0.20	0.50	0.29
6	23, 27, 70, 72, 39, 67, 40, 71, 81, 29	8, 29, 72, 28, 39, 61, 40	4	7	0.40	0.57	0.47
7	65, 25, 67, 64, 4, 1, 31, 44, 20, 23, 30	74, 30, 65, 37, 40, 49, 25, 41, 27	3	9	0.30	0.33	0.32
8	28, 49, 23, 27, 70, 42, 71, 67, 81, 29	90, 8, 29, 61, 28, 39, 72, 40	1	8	0.10	0.13	0.11
9	64, 65, 44, 80, 26, 41, 30, 23, 20, 31	31, 37, 13, 64, 45	2	5	0.20	0.40	0.27
10	56, 19, 35, 89, 49, 37, 52, 41, 5, 33	33, 56	2	2	0.20	1.00	0.33
11	3, 83, 40, 19, 85, 81, 57, 2, 71, 34	34, 39	1	2	0.10	0.50	0.17
12	4, 27, 26, 25, 22, 31, 20, 30, 23, 44	74, 84, 27, 44, 46, 49, 61, 45, 50	2	9	0.20	0.22	0.21
13	31, 25, 41, 21, 23, 44, 22, 30, 45, 20	44, 45, 69, 27, 25, 26	3	6	0.30	0.50	0.38
14	77, 79, 36, 65, 35, 25, 30, 69, 71, 47	47, 83, 89	1	3	0.10	0.33	0.15
15	4, 70, 66, 21, 3, 63, 52, 13, 26, 48	48, 49, 52, 26, 42	3	5	0.30	0.60	0.40
16	30, 81, 36, 31, 25, 80, 65, 64, 67, 49	49, 65, 41, 67, 30, 45, 64, 36	6	8	0.60	0.75	0.67
17	55, 44, 58, 37, 22, 20, 49, 23, 26, 50	50, 61, 46, 44, 49, 43	3	6	0.30	0.50	0.38
18	68, 20, 40, 12, 38, 78, 1, 27, 15, 51	77, 51, 1, 15, 27, 12, 40	6	7	0.60	0.86	0.71
19	36, 27, 65, 3, 37, 48, 23, 63, 13, 52	48, 52, 63, 61, 26, 13	4	6	0.40	0.67	0.50
20	84, 59, 85, 81, 67, 70, 53, 65, 64, 68	85, 65, 41, 66	2	4	0.20	0.50	0.29
21	65, 62, 67, 59, 68, 64, 57, 53, 21, 54	54, 62	2	2	0.20	1.00	0.33
22	68, 65, 85, 74, 67, 62, 81, 57, 80, 56	80, 7, 2, 56, 41	2	5	0.20	0.40	0.27
23	53, 72, 56, 64, 65, 59, 58, 62, 67, 57	88, 87, 57, 59, 37, 65	3	6	0.30	0.50	0.38
24	80, 67, 62, 56, 25, 59, 53, 64, 57, 58	87, 54, 58, 37, 41, 65, 57	2	7	0.20	0.29	0.24
25	65, 80, 35, 81, 66, 70, 68, 53, 64, 60	80, 85, 53, 60, 66, 11, 90	4	7	0.40	0.57	0.47
26	54, 65, 30, 56, 15, 67, 28, 62, 64, 61	76, 7, 4, 61, 41, 42, 37	1	7	0.10	0.14	0.12
27	54, 44, 23, 58, 57, 74, 59, 67, 62, 64	54, 62, 65, 27	2	4	0.20	0.50	0.29
28	37, 3, 4, 15, 52, 48, 26, 13, 65, 63	48, 52, 62, 63, 37	4	5	0.40	0.80	0.53
29	72, 63, 49, 65, 86, 57, 90, 67, 62, 64	69, 48, 64, 65, 67, 85, 61	3	7	0.30	0.43	0.35
30	81, 85, 80, 68, 53, 59, 70, 67, 64, 65	69, 87, 1, 53, 65, 47, 64, 70, 85	5	9	0.50	0.56	0.53
31	55, 65, 68, 64, 60, 56, 23, 14, 31, 66	80, 90, 66, 60, 53, 14	3	6	0.30	0.50	0.38
32	81, 90, 67, 85, 53, 59, 70, 65, 64, 68	85, 76, 68, 70, 36, 80, 69, 65, 66	4	9	0.40	0.44	0.42
33	76, 80, 27, 65, 67, 22, 25, 30, 42, 69	69, 76, 1, 65, 45, 46, 61, 28	3	8	0.30	0.38	0.33
34	81, 85, 59, 67, 53, 68, 65, 64, 70, 90	68, 70, 40, 53, 72, 66, 73, 4, 41	3	9	0.30	0.33	0.32
35	86, 29, 23, 39, 90, 57, 35, 74, 81, 71	71, 39, 40	2	3	0.20	0.67	0.31
36	12, 59, 62, 64, 68, 72, 67, 13, 57, 74	74, 50, 1, 37, 13, 43, 12, 42	3	8	0.30	0.38	0.33
37	15, 43, 42, 69, 26, 12, 51, 63, 36, 77	76, 77, 61, 51, 79, 37	2	6	0.20	0.33	0.25
38	30, 56, 90, 36, 28, 89, 69, 77, 83, 78	83, 77, 78, 47	3	4	0.30	0.75	0.43
39	57, 37, 85, 30, 80, 77, 3, 28, 12, 79	79, 12	2	2	0.20	1.00	0.33
40	60, 4, 49, 56, 23, 70, 31, 53, 81, 80	80, 53, 60, 66	3	4	0.30	0.75	0.43
41	57, 65, 70, 29, 23, 72, 40, 81, 39, 71	90, 7, 81, 73, 38, 32, 39, 40, 37	3	9	0.30	0.33	0.32
42	80, 57, 19, 31, 37, 36, 70, 85, 89, 82	7, 82, 12	1	3	0.10	0.33	0.15
43	71, 57, 35, 84, 85, 25, 47, 49, 30, 83	83, 89, 47, 37, 85	3	5	0.30	0.60	0.40
44	57, 49, 58, 65, 7, 15, 26, 36, 37, 84	77, 84, 46, 44, 37	2	5	0.20	0.40	0.27
45	57, 56, 59, 67, 70, 68, 53, 65, 64, 85	53, 65, 66, 85 68, 25, 90	4	7	0.40	0.57	0.47
46	1, 34, 2, 81, 26, 85, 80, 4, 27, 86	86, 1, 43	2	3	0.20	0.67	0.31
47	68, 84, 64, 67, 15, 4, 59, 53, 87	82, 62, 87, 53, 70, 14	2	6	0.20	0.33	0.25
48	90, 29, 70, 39, 81, 72, 71, 40, 88, 18	88, 43, 39	2	3	0.20	0.67	0.31
49	7, 58, 14, 28, 26, 84, 32, 48, 66, 89	89, 83, 2, 47	1	4	0.10	0.25	0.14
50	66, 71, 68, 18, 64, 60, 81, 29, 70, 90	90, 29, 72, 4, 38, 66, 39	3	7	0.30	0.43	0.35
51	62, 86, 12, 15, 27, 56, 28, 4, 85, 1	74, 1, 50, 15, 37, 27, 40	3	7	0.30	0.43	0.35
52	34, 55, 86, 81, 44, 80, 27, 4, 56, 2	2, 4, 55, 56, 15	4	5	0.40	0.80	0.53
53	27, 53, 60, 65, 28, 32, 14, 13, 7, 3	7, 5, 3, 32, 37	3	5	0.30	0.60	0.40
54	86, 3, 37, 81, 14, 27, 60, 28, 1, 4	90, 2, 4, 1, 29, 84, 27, 18	3	8	0.30	0.38	0.33
55	37, 23, 27, 19, 85, 86, 84, 57, 66, 7	7, 88, 58, 59, 54, 57	2	6	0.20	0.33	0.25
56	34, 68, 28, 13, 65, 23, 71, 48, 66, 63	8, 39, 47, 46, 81	0	5	0.00	0.00	0.00
57	85, 18, 49, 15, 13, 71, 42, 69, 27, 76	76, 40, 61, 1	1	4	0.10	0.25	0.14
58	81, 22, 21, 26, 44, 23, 31, 30, 20, 41	56, 37, 41, 20, 40	2	5	0.20	0.40	0.27
59	62, 59, 65, 57, 27, 11, 68, 22, 28, 42	76, 11, 42, 61, 22	3	5	0.30	0.60	0.40
60	38, 69, 81, 1, 51, 74, 40, 27, 15, 43	76, 74, 43, 1, 90, 15, 27	5	7	0.50	0.71	0.59
61	53, 31, 74, 23, 11, 42, 28, 65, 14, 19	22, 42, 11	2	3	0.20	0.67	0.31
62	74, 4, 26, 44, 63, 23, 3, 48, 52, 13	74, 1, 50, 67, 13, 61, 52, 48, 63, 37	5	10	0.50	0.50	0.50
63	74, 4, 26, 44, 63, 23, 3, 48, 52, 13	18	0	1	0.00	0.00	0.00
64	76, 57, 29, 83, 27, 23, 81, 74, 34, 19	19	1	1	0.10	1.00	0.18
65	64, 21, 22, 44, 41, 26, 31, 30, 23, 20	45, 69, 62, 7, 65, 20, 48, 63	1	8	0.10	0.13	0.11
66	31, 4, 59, 45, 20, 44, 23, 28, 22, 54	11, 22, 52, 48, 63, 23	2	6	0.20	0.33	0.25
67	7, 4, 23, 87, 63, 58, 53, 54, 66, 14	80, 1, 14, 90, 60, 66	2	6	0.20	0.33	0.25
68	67, 41, 20, 15, 30, 23, 25, 24, 54, 21	21, 31, 76, 20	2	4	0.20	0.50	0.29
69	65, 26, 28, 20, 22, 25, 45, 44, 30, 23	72, 74, 73, 23, 21, 20, 26, 28	4	8	0.40	0.50	0.44
70	81, 89, 67, 42, 80, 79, 57, 21, 56, 24	24, 21, 2, 60	2	4	0.20	0.50	0.29

Average					0.26	0.49	0.34

*Yoruba language*							
1	37, 41, 12, 53, 54, 65, 67, 70, 81, 84,	8, 46, 37, 41, 44, 20, 22	2	7	0.20	0.29	0.24
2	22, 25, 42, 11, 28, 46, 62, 65, 87, 4	85, 12, 46, 37, 39, 8	1	6	0.10	0.17	0.13
3		81, 34, 72, 38, 40, 37	0	6	0.00	0.00	0.00
4	22, 42, 11, 12, 28, 51, 62, 63, 77, 79	51, 11, 12, 43	3	4	0.30	0.75	0.43
5	22, 23, 42, 72, 4, 59, 31, 28, 11, 65	83, 11, 28, 37	2	4	0.20	0.50	0.29
6	18, 23, 24, 39, 40, 29, 90, 72, 81, 70	8, 29, 72, 28, 39, 61, 40	4	7	0.40	0.57	0.47
7	20, 21, 31, 22, 30, 23, 44, 45, 59, 25	74, 30, 65, 37, 40, 49, 25, 41, 27	2	9	0.20	0.22	0.21
8	23, 24, 39, 40, 42, 29, 31, 70, 81, 90	90, 8, 29, 61, 28, 39, 72, 40	4	8	0.40	0.50	0.44
9	14, 37, 29, 32, 44, 60, 66, 80, 81, 84	31, 37, 13, 64, 45	1	5	0.10	0.20	0.13
10	14, 21, 37, 41, 33, 54, 56, 60, 79, 5	33, 56	2	2	0.20	1.00	0.33
11	23, 34, 48, 57, 67, 71, 85, 3, 1, 2	34, 39	1	2	0.10	0.50	0.17
12	22, 23, 27, 41, 28, 30, 44, 63, 1, 4	74, 84, 27, 44, 46, 49, 61, 45, 50	2	9	0.20	0.22	0.21
13	20, 13, 23, 41, 22, 30, 44, 4, 45, 49	44, 45, 69, 27, 25, 26	2	6	0.20	0.33	0.25
14	25, 23, 12, 47, 51, 65, 77, 79, 83, 86	47, 83, 89	2	3	0.20	0.67	0.31
15	13, 26, 41, 42, 28, 48, 85, 4, 1, 3	48, 49, 52, 26, 42	3	5	0.30	0.60	0.40
16	41, 28, 31, 49, 58, 81, 85, 86, 3, 1	49, 65, 41, 67, 30, 45, 64, 36	2	8	0.20	0.25	0.22
17	36, 23, 30, 46, 58, 62, 65, 84, 85, 87	50, 61, 46, 44, 49, 43	1	6	0.10	0.17	0.13
18	15, 18, 27, 38, 12, 51, 77, 78, 79, 1	77, 51, 1, 15, 27, 12, 40	6	7	0.60	0.86	0.71
19	13, 21, 26, 37, 41, 52, 63, 65, 84, 1	48, 52, 63, 61, 26, 13	4	6	0.40	0.67	0.50
20	20, 41, 43, 59, 64, 65, 67, 70, 84, 87	85, 65, 41, 66	2	4	0.20	0.50	0.29
21	53, 21, 54, 62, 57, 59, 58, 67, 68, 64	54, 62	2	2	0.20	1.00	0.33
22	72, 80, 81, 57, 56, 62, 58, 64, 59, 67	80, 7, 2, 56, 41	2	5	0.20	0.40	0.27
23	15, 19, 37, 11, 32, 64, 72, 78, 86, 87	88, 87, 57, 59, 37, 65	2	6	0.20	0.33	0.25
24	14, 15, 23, 31, 53, 58, 64, 66, 67, 68	87, 54, 58, 37, 41, 65, 57	1	7	0.10	0.14	0.12
25	23, 31, 53, 60, 65, 66, 80, 81, 7, 3	80, 85, 53, 60, 66, 11, 90	4	7	0.40	0.57	0.47
26	59, 61, 62, 64, 65, 67, 68, 70, 72, 90	76, 7, 4, 61, 41, 42, 37	1	7	0.10	0.14	0.12
27	62, 59, 58, 23, 65, 64, 20, 74, 67, 57	54, 62, 65, 27	2	4	0.20	0.50	0.29
28	13, 26, 37, 30, 44, 49, 52, 62, 63, 4	48, 52, 62, 63, 37	4	5	0.40	0.80	0.53
29	57, 58, 61, 62, 64, 67, 70, 72, 74, 85	69, 48, 64, 65, 67, 85, 61	4	7	0.40	0.57	0.47
30	56, 57, 59, 64, 65, 67, 70, 72, 81, 84	69, 87, 1, 53, 65, 47, 64, 70, 85	3	9	0.30	0.33	0.32
31	20, 43, 45, 57, 59, 64, 65, 70, 72, 29	80, 90, 66, 60, 53, 14	0	6	0.00	0.00	0.00
32	22, 53, 57, 59, 64, 65, 67, 68, 70, 72	85, 76, 68, 70, 36, 80, 69, 65, 66	3	9	0.30	0.33	0.32
33	14, 25, 36, 42, 28, 60, 77, 81, 87, 7,	69, 76, 1, 65, 45, 46, 61, 28	1	8	0.10	0.13	0.11
34	53, 57, 59, 64, 65, 67, 70, 72, 81, 90	68, 70, 40, 53, 72, 66, 73, 4, 41	3	9	0.30	0.33	0.32
35	13, 25, 26, 39, 40, 29, 34, 65, 72, 81	71, 39, 40	2	3	0.20	0.67	0.31
36	13, 57, 59, 61, 62, 64, 67, 68, 72, 74	74, 50, 1, 37, 13, 43, 12, 42	2	8	0.20	0.25	0.22
37	26, 36, 43, 12, 51, 63, 69, 77, 84, 3	76, 77, 61, 51, 79, 37	2	6	0.20	0.33	0.25
38	15, 24, 27, 42, 33, 45, 53, 60, 69, 78	83, 77, 78, 47	1	4	0.10	0.25	0.14
39	79, 77, 12, 80, 28, 37, 34, 81, 78, 44	79, 12	2	2	0.20	1.00	0.33
40	80, 23, 27, 41, 42, 31, 34, 81, 1, 4	80, 53, 60, 66	1	4	0.10	0.25	0.14
41	23, 27, 40, 58, 60, 70, 72, 81, 90, 7	90, 7, 81, 73, 38, 32, 39, 40, 37	4	9	0.40	0.44	0.42
42	85, 82, 25, 36, 37, 12, 34, 86, 89, 1	7, 82, 12	2	3	0.20	0.67	0.31
43	83, 86, 30, 84, 47, 57, 19, 81, 79, 34	83, 89, 47, 37, 85	2	5	0.20	0.40	0.27
44	64, 53, 70, 65, 81, 85, 60, 67, 68, 80	77, 84, 46, 44, 37	0	5	0.00	0.00	0.00
45	86, 80, 81, 4, 26, 34, 53, 27, 29, 84	53, 65, 66, 85 68, 25, 90	1	7	0.10	0.14	0.12
46	22, 87, 15, 53, 37, 57, 63, 65, 81, 4	86, 1, 43	0	3	0.00	0.00	0.00
47	70, 86, 71, 64, 37, 65, 27, 88, 43, 63	82, 62, 87, 53, 70, 14	1	6	0.10	0.17	0.13
48	88, 70, 73, 27, 37, 43, 64, 65, 71, 86	88, 43, 39	2	3	0.20	0.67	0.31
49	66, 32, 58, 14, 30, 48, 59, 81, 84, 89	89, 83, 2, 47	1	4	0.10	0.25	0.14
50	39, 29, 90, 89, 71, 72, 81, 89, 23, 40	90, 29, 72, 4, 38, 66, 39	4	7	0.40	0.57	0.47
51	1, 4, 22, 18, 27, 28, 59, 62, 65, 70	74, 1, 50, 15, 37, 27, 40	2	7	0.20	0.29	0.24
52	19, 56, 85, 13, 34, 47, 79, 80, 86, 3	2, 4, 55, 56, 15	1	5	0.10	0.20	0.13
53	7, 3, 70, 4, 81, 60, 58, 32, 31, 27	7, 5, 3, 32, 37	3	5	0.30	0.60	0.40
54	4, 1, 13, 28, 54, 58, 60, 67, 70, 7	90, 2, 4, 1, 29, 84, 27, 18	2	8	0.20	0.25	0.22
55	24, 39, 12, 33, 57, 69, 85, 86, 89, 1	7, 88, 58, 59, 54, 57	1	6	0.10	0.17	0.13
56	25, 42, 54, 58, 63, 66, 68, 87, 1, 4	8, 39, 47, 46, 81	0	5	0.00	0.00	0.00
57	76, 71, 69, 51, 47, 42, 40, 36, 27, 15	76, 40, 61, 1	2	4	0.20	0.50	0.29
58	41, 29, 32, 59, 65, 67, 74, 81, 84, 87	56, 37, 41, 20, 40	1	5	0.10	0.20	0.13
59	11, 42, 28, 23, 22, 57, 62, 67, 79, 85	76, 11, 42, 61, 22	3	5	0.30	0.60	0.40
60	27, 15, 43, 13, 36, 69, 40, 51, 1, 4	76, 74, 43, 1, 90, 15, 27	4	7	0.40	0.57	0.47
61		22, 42, 11	0	3	0.00	0.00	0.00
62	13, 4, 1, 74, 69, 68, 63, 52, 26, 22	74, 1, 50, 67, 13, 61, 52, 48, 63, 37	5	10	0.50	0.50	0.50
63	18, 71, 29, 23, 26, 39, 40, 34, 49, 72	18	1	1	0.10	1.00	0.18
64	19, 85, 42, 32, 34, 56, 62, 80, 81, 86	19	1	1	0.10	1.00	0.18
65	41, 20, 22, 23, 30, 44, 59, 65, 67, 70	45, 69, 62, 7, 65, 20, 48, 63	2	8	0.20	0.25	0.22
66	28, 22, 42, 11, 23, 31, 54, 59, 65, 68	11, 22, 52, 48, 63, 23	3	6	0.30	0.50	0.38
67	14, 23, 48, 53, 54, 57, 65, 58, 66, 68	80, 1, 14, 90, 60, 66	2	6	0.20	0.33	0.25
68	21, 24, 11, 30, 52, 67, 53, 88, 54, 58	21, 31, 76, 20	1	4	0.10	0.25	0.14
69	23, 30, 22, 44, 25, 59, 64, 69, 67, 70	72, 74, 73, 23, 21, 20, 26, 28	1	8	0.10	0.13	0.11
70	24, 15, 21, 12, 32, 34, 49, 58, 84, 1	24, 21, 2, 60	2	4	0.20	0.50	0.29

Average					0.20	0.41	0.27
Parity with English language					78.89%	83.13%	80.30%

*Igbo language*							
1	66, 20, 84, 13, 26, 4, 74, 22, 45, 37	8, 46, 37, 41, 44, 20, 22	3	7	0.30	0.43	0.35
2	46, 89, 23, 28, 85, 7, 87, 29, 41, 42	85, 12, 46, 37, 39, 8	2	6	0.20	0.33	0.25
3	83, 39, 28, 23, 25, 34, 80, 40, 81, 37	81, 34, 72, 38, 40, 37	4	6	0.40	0.67	0.50
4	47, 43, 69, 23, 1, 51, 42, 12, 30, 79	51, 11, 12, 43	3	4	0.30	0.75	0.43
5	28, 42, 65, 67, 22, 11, 23, 69, 27, 30	83, 11, 28, 37	2	4	0.20	0.50	0.29
6	29, 81, 72, 90, 39, 67, 70, 23, 40, 71	8, 29, 72, 28, 39, 61, 40	4	7	0.40	0.57	0.47
7	59, 67, 64, 41, 65, 22, 30, 23, 50, 20	74, 30, 65, 37, 40, 49, 25, 41, 27	3	9	0.30	0.33	0.32
8	21, 81, 90, 71, 72, 67, 70, 65, 23, 28	90, 8, 29, 61, 28, 39, 72, 40	3	8	0.30	0.38	0.33
9	33, 41, 5, 52, 37, 81, 89, 80, 49, 35	31, 37, 13, 64, 45	1	5	0.10	0.20	0.13
10	34, 71, 39, 25, 57, 2, 3, 83, 37, 81	33, 56	0	2	0.00	0.00	0.00
11	41, 23, 7, 85, 59, 62, 37, 22, 46, 31	34, 39	0	2	0.00	0.00	0.00
12	45, 22, 23, 44, 20, 18, 4, 30, 21, 13	74, 84, 27, 44, 46, 49, 61, 45, 50	2	9	0.20	0.22	0.21
13	47, 36, 30, 65, 86, 69, 26, 83, 67, 45	44, 45, 69, 27, 25, 26	3	6	0.30	0.50	0.38
14	26, 36, 30, 45, 47, 65, 67, 69, 71, 86	47, 83, 89	1	3	0.10	0.33	0.15
15	13, 22, 26, 28, 45, 63, 69, 85, 3, 4	48, 49, 52, 26, 42	1	5	0.10	0.20	0.13
16	49, 64, 67, 68, 80, 81, 85, 1, 4, 7	49, 65, 41, 67, 30, 45, 64, 36	3	8	0.30	0.38	0.33
17	23, 63, 50, 46, 48, 27, 25, 58, 66, 42	50, 61, 46, 44, 49, 43	2	6	0.20	0.33	0.25
18	51, 83, 78, 26, 27, 77, 79, 12, 76, 37	77, 51, 1, 15, 27, 12, 40	4	7	0.40	0.57	0.47
19	55, 42, 85, 52, 76, 26, 13, 63, 35, 69	48, 52, 63, 61, 26, 13	4	6	0.40	0.67	0.50
20	31, 67, 58, 30, 65, 4, 23, 53, 64, 87	85, 65, 41, 66	1	4	0.10	0.25	0.14
21	54, 53, 64, 20, 67, 34, 58, 57, 68, 21	54, 62	1	2	0.10	0.50	0.17
22	28, 62, 44, 85, 60, 20, 31, 68, 41, 3	80, 7, 2, 56, 41	0	5	0.00	0.00	0.00
23	58, 59, 2, 25, 75, 12, 73, 14, 57, 62	88, 87, 57, 59, 37, 65	2	6	0.20	0.33	0.25
24	67, 66, 53, 58, 14, 59, 86, 45, 15, 25	87, 54, 58, 37, 41, 65, 57	1	7	0.10	0.14	0.12
25	60, 4, 81, 23, 65, 85, 3, 80, 14, 28	80, 85, 53, 60, 66, 11, 90	3	7	0.30	0.43	0.35
26	30, 18, 37, 53, 36, 28, 23, 4, 47, 44	76, 7, 4, 61, 41, 42, 37	1	7	0.10	0.14	0.12
27	62, 23, 44, 22, 30, 20, 4, 66, 13, 25	54, 62, 65, 27	1	4	0.10	0.25	0.14
28	63, 13, 52, 26, 65, 3, 62, 80, 81, 37	48, 52, 62, 63, 37	4	5	0.40	0.80	0.53
29	25, 49, 57, 58, 62, 64, 65, 71, 69, 67	69, 48, 64, 65, 67, 85, 61	4	7	0.40	0.57	0.47
30	44, 49, 62, 64, 65, 67, 68, 85, 86, 87	69, 87, 1, 53, 65, 47, 64, 70, 85	4	9	0.40	0.44	0.42
31	56, 60, 7, 4, 81, 68, 66, 53, 49, 29	80, 90, 66, 60, 53, 14	3	6	0.30	0.50	0.38
32	68, 84, 64, 22, 25, 65, 27, 67, 70, 81	85, 76, 68, 70, 36, 80, 69, 65, 66	3	9	0.30	0.33	0.32
33	44, 42, 66, 72, 64, 49, 59, 60, 27, 23	69, 76, 1, 65, 45, 46, 61, 28	0	8	0.00	0.00	0.00
34	64, 68, 13, 27, 38, 53, 65, 70, 81, 85	68, 70, 40, 53, 72, 66, 73, 4, 41	3	9	0.30	0.33	0.32
35	71, 12, 13, 1, 68, 65, 62, 57, 54, 43	71, 39, 40	1	3	0.10	0.33	0.15
36	45, 43, 22, 20, 23, 25, 30, 60, 75, 87	74, 50, 1, 37, 13, 43, 12, 42	1	8	0.10	0.13	0.11
37	15, 24, 35, 42, 43, 33, 52, 55, 69, 78	76, 77, 61, 51, 79, 37	0	6	0.00	0.00	0.00
38	79, 77, 83, 28, 85, 78, 37, 34, 57, 12	83, 77, 78, 47	3	4	0.30	0.75	0.43
39	21, 37, 11, 12, 28, 51, 66, 77, 79, 83	79, 12	2	2	0.20	1.00	0.33
40	22, 23, 24, 31, 53, 56, 60, 67, 80, 81	80, 53, 60, 66	3	4	0.30	0.75	0.43
41	18, 23, 29, 39, 40, 7, 71, 72, 81, 90	90, 7, 81, 73, 38, 32, 39, 40, 37	5	9	0.50	0.56	0.53
42	83, 25, 77, 85, 30, 35, 71, 28, 79, 34	7, 82, 12	0	3	0.00	0.00	0.00
43	18, 35, 30, 34, 47, 49, 57, 71, 83, 85	83, 89, 47, 37, 85	3	5	0.30	0.60	0.40
44	65, 53, 11, 70, 85, 22, 25, 59, 67, 81	77, 84, 46, 44, 37	0	5	0.00	0.00	0.00
45	13, 26, 27, 30, 34, 80, 2, 81, 86, 4	53, 65, 66, 85 68, 25, 90	0	7	0.00	0.00	0.00
46	26, 27, 34, 51, 59, 81, 85, 86, 4, 87	86, 1, 43	1	3	0.10	0.33	0.15
47	22, 53, 59, 62, 64, 63, 70, 65, 67, 87	82, 62, 87, 53, 70, 14	4	6	0.40	0.67	0.50
48	18, 38, 39, 71, 81, 40, 72, 88, 90, 70	88, 43, 39	2	3	0.20	0.67	0.31
49	21, 25, 26, 48, 66, 70, 77, 81, 84, 89	89, 83, 2, 47	1	4	0.10	0.25	0.14
50	14, 18, 22, 54, 23, 60, 4, 29, 70, 90	90, 29, 72, 4, 38, 66, 39	3	7	0.30	0.43	0.35
51	15, 27, 37, 56, 80, 3, 4, 87, 1, 81, 4	74, 1, 50, 15, 37, 27, 40	4	7	0.40	0.57	0.47
52	19, 34, 44, 49, 55, 80, 56, 81, 2, 4	2, 4, 55, 56, 15	4	5	0.40	0.80	0.53
53	37, 31, 32, 70, 64, 56, 3, 4, 7, 53	7, 5, 3, 32, 37	4	5	0.40	0.80	0.53
54	15, 18, 37, 28, 31, 55, 81, 90, 1, 4	90, 2, 4, 1, 29, 84, 27, 18	4	8	0.40	0.50	0.44
55	14, 20, 45, 57, 58, 65, 66, 80, 81, 7	7, 88, 58, 59, 54, 57	3	6	0.30	0.50	0.38
56	13, 23, 36, 58, 63, 66, 64, 65, 71, 81	8, 39, 47, 46, 81	1	5	0.10	0.20	0.13
57	18, 19, 27, 42, 47, 51, 62, 69, 1, 76	76, 40, 61, 1	2	4	0.20	0.50	0.29
58	41, 56, 59, 64, 70, 65, 67, 75, 81, 87	56, 37, 41, 20, 40	2	5	0.20	0.40	0.27
59	22, 23, 42, 26, 11, 28, 46, 54, 61, 62	76, 11, 42, 61, 22	4	5	0.40	0.80	0.53
60	15, 23, 27, 40, 43, 51, 62, 64, 74, 1	76, 74, 43, 1, 90, 15, 27	5	7	0.50	0.71	0.59
61	22, 59, 23, 42, 28, 31, 11, 63, 65, 4	22, 42, 11	3	3	0.30	1.00	0.46
62	13, 26, 37, 48, 52, 63, 65, 68, 1, 4	74, 1, 50, 67, 13, 61, 52, 48, 63, 37	6	10	0.60	0.60	0.60
63	18, 20, 35, 36, 40, 29, 49, 62, 71, 81	18	1	1	0.10	1.00	0.18
64	19, 34, 47, 57, 71, 72, 79, 80, 81, 83	19	1	1	0.10	1.00	0.18
65	20, 22, 23, 27, 41, 30, 44, 45, 59, 65	45, 69, 62, 7, 65, 20, 48, 63	3	8	0.30	0.38	0.33
66	13, 22, 23, 11, 28, 31, 44, 45, 59, 4	11, 22, 52, 48, 63, 23	3	6	0.30	0.50	0.38
67	22, 28, 54, 58, 59, 66, 81, 87, 4, 7	80, 1, 14, 90, 60, 66	1	6	0.10	0.17	0.13
68	21, 24, 41, 42, 54, 58, 67, 69, 88, 90	21, 31, 76, 20	1	4	0.10	0.25	0.14
69	20, 22, 23, 25, 26, 41, 30, 44, 45, 65	72, 74, 73, 23, 21, 20, 26, 28	3	8	0.30	0.38	0.33
70	21, 24, 11, 34, 56, 57, 79, 80, 81, 89	24, 21, 2, 60	2	4	0.20	0.50	0.29

Average					0.23	0.43	0.30
Parity with English language					90.00%	88.87%	89.61%

*Hausa language*							
1	13, 26, 27, 37, 44, 58, 65, 84, 85, 4	8, 46, 37, 41, 44, 20, 22	2	7	0.20	0.29	0.24
2	23, 27, 37, 28, 46, 61, 62, 87, 1, 7	85, 12, 46, 37, 39, 8	2	6	0.20	0.33	0.25
3	18, 23, 27, 38, 39, 40, 59, 71, 72, 81	81, 34, 72, 38, 40, 37	4	6	0.40	0.67	0.50
4	7, 79, 77, 51, 28, 12, 11, 42, 26, 13	51, 11, 12, 43	2	4	0.20	0.50	0.29
5	22, 23, 42, 11, 28, 31, 54, 59, 68, 83	83, 11, 28, 37	3	4	0.30	0.75	0.43
6	23, 39, 40, 29, 49, 59, 70, 71, 72, 81	8, 29, 72, 28, 39, 61, 40	4	7	0.40	0.57	0.47
7	4, 67, 64, 59, 30, 27, 25, 22, 20, 13	74, 30, 65, 37, 40, 49, 25, 41, 27	3	9	0.30	0.33	0.32
8	81, 72, 71, 65, 59, 29, 28, 40, 39, 23	90, 8, 29, 61, 28, 39, 72, 40	4	8	0.40	0.50	0.44
9	15, 19, 23, 35, 33, 52, 59, 89, 2, 3	31, 37, 13, 64, 45	0	5	0.00	0.00	0.00
10	3, 2, 89, 59, 52, 33, 35, 23, 19, 15	33, 56	1	2	0.10	0.50	0.17
11	23, 34, 48, 57, 67, 71, 81, 85, 2, 4	34, 39	1	2	0.10	0.50	0.17
12	87, 85, 67, 65, 46, 44, 30, 25, 23, 22	74, 84, 27, 44, 46, 49, 61, 45, 50	2	9	0.20	0.22	0.21
13	20, 21, 22, 23, 25, 30, 27, 44, 54, 4	44, 45, 69, 27, 25, 26	3	6	0.30	0.50	0.38
14	35, 36, 30, 47, 49, 64, 65, 69, 74, 83	47, 83, 89	2	3	0.20	0.67	0.31
15	26, 13, 22, 37, 28, 52, 63, 78, 3, 4	48, 49, 52, 26, 42	2	5	0.20	0.40	0.27
16	3, 81, 80, 74, 67, 64, 49, 31, 30, 27	49, 65, 41, 67, 30, 45, 64, 36	4	8	0.40	0.50	0.44
17	64, 59, 54, 53, 50, 49, 44, 22, 14, 13	50, 61, 46, 44, 49, 43	3	6	0.30	0.50	0.38
18	1, 79, 78, 77, 51, 12, 40, 38, 27, 14	77, 51, 1, 15, 27, 12, 40	6	7	0.60	0.86	0.71
19	26, 13, 23, 37, 28, 48, 52, 57, 63, 3	48, 52, 63, 61, 26, 13	4	6	0.40	0.67	0.50
20	28, 59, 64, 65, 67, 68, 80, 81, 87, 7	85, 65, 41, 66	1	4	0.10	0.25	0.14
21	87, 74, 69, 68, 66, 58, 57, 54, 53, 23	54, 62	1	2	0.10	0.50	0.17
22	11, 28, 34, 44, 56, 57, 67, 80, 85, 1	80, 7, 2, 56, 41	2	5	0.20	0.40	0.27
23	14, 20, 57, 58, 59, 60, 65, 66, 88, 7	88, 87, 57, 59, 37, 65	4	6	0.40	0.67	0.50
24	20, 23, 58, 65, 66, 68, 74, 69, 85, 7	87, 54, 58, 37, 41, 65, 57	2	7	0.20	0.29	0.24
25	65, 59, 58, 53, 44, 28, 11, 41, 27, 23	80, 85, 53, 60, 66, 11, 90	2	7	0.20	0.29	0.24
26		76, 7, 4, 61, 41, 42, 37	0	7	0.00	0.00	0.00
27		54, 62, 65, 27	0	4	0.00	0.00	0.00
28	51, 52, 63, 2, 3, 4, 13, 26, 28, 48	48, 52, 62, 63, 37	2	5	0.20	0.40	0.27
29	23, 26, 44, 45, 49, 58, 63, 81, 84, 4	69, 48, 64, 65, 67, 85, 61	0	7	0.00	0.00	0.00
30	22, 23, 42, 28, 59, 64, 65, 67, 84, 4	69, 87, 1, 53, 65, 47, 64, 70, 85	2	9	0.20	0.22	0.21
31	14, 31, 49, 56, 57, 58, 59, 66, 4, 7	80, 90, 66, 60, 53, 14	2	6	0.20	0.33	0.25
32	11, 23, 28, 59, 65, 67, 84, 68, 87, 4	85, 76, 68, 70, 36, 80, 69, 65, 66	2	9	0.20	0.22	0.21
33	26, 20, 23, 44, 28, 57, 63, 84, 65, 4	69, 76, 1, 65, 45, 46, 61, 28	2	8	0.20	0.25	0.22
34	70, 68, 67, 65, 22, 23, 40, 41, 59, 38	68, 70, 40, 53, 72, 66, 73, 4, 41	4	9	0.40	0.44	0.42
35	23, 35, 34, 49, 57, 65, 67, 71, 83, 85	71, 39, 40	1	3	0.10	0.33	0.15
36	74, 68, 57, 46, 28, 12, 42, 23, 13, 22	74, 50, 1, 37, 13, 43, 12, 42	4	8	0.40	0.50	0.44
37	26, 36, 42, 12, 51, 52, 63, 77, 78, 86	76, 77, 61, 51, 79, 37	2	6	0.20	0.33	0.25
38	21, 22, 26, 11, 45, 48, 51, 78, 87, 5	83, 77, 78, 47	1	4	0.10	0.25	0.14
39	79, 77, 57, 66, 51, 28, 12 11, 21, 13	79, 12	2	2	0.20	1.00	0.33
40	23, 37, 28, 31, 56, 57, 58, 64, 65, 84	80, 53, 60, 66	0	4	0.00	0.00	0.00
41	14, 23, 37, 28, 59, 64, 65, 66, 3, 7	90, 7, 81, 73, 38, 32, 39, 40, 37	2	9	0.20	0.22	0.21
42	25, 42, 12, 28, 34, 57, 77, 82, 85, 89	7, 82, 12	2	3	0.20	0.67	0.31
43	84, 83, 71, 57, 49, 30, 35, 26, 25	83, 89, 47, 37, 85	1	5	0.10	0.20	0.13
44	37, 28, 31, 53, 57, 60, 64, 65, 84, 4	77, 84, 46, 44, 37	2	5	0.20	0.40	0.27
45	82, 81, 68, 67, 65, 59, 31, 11, 42, 25	53, 65, 66, 85 68, 25, 90	3	7	0.30	0.43	0.35
46	24, 26, 27, 34, 49, 59, 80, 84, 86, 4	86, 1, 43	1	3	0.10	0.33	0.15
47	15, 22, 42, 11, 28, 53, 59, 65, 67, 87	82, 62, 87, 53, 70, 14	2	6	0.20	0.33	0.25
48	37, 57, 59, 65, 71, 73, 81, 86, 88, 1	88, 43, 39	1	3	0.10	0.33	0.15
49	28, 32, 49, 58, 66, 77, 86, 87, 89, 3	89, 83, 2, 47	1	4	0.10	0.25	0.14
50	18, 23, 27, 38, 29, 70, 72, 81, 90, 4	90, 29, 72, 4, 38, 66, 39	5	7	0.50	0.71	0.59
51	4, 1, 86, 85, 56, 28, 12, 40, 27, 15	74, 1, 50, 15, 37, 27, 40	4	7	0.40	0.57	0.47
52	27, 40, 30, 34, 56, 80, 81, 87, 2, 4	2, 4, 55, 56, 15	3	5	0.30	0.60	0.40
53	20, 45, 57, 58, 60, 66, 68, 3, 4, 7	7, 5, 3, 32, 37	2	5	0.20	0.40	0.27
54	14, 27, 37, 28, 60, 81, 86, 87, 1, 4	90, 2, 4, 1, 29, 84, 27, 18	3	8	0.30	0.38	0.33
55	24, 12, 28, 56, 57, 60, 63, 85, 89, 1	7, 88, 58, 59, 54, 57	1	6	0.10	0.17	0.13
56		8, 39, 47, 46, 81	0	5	0.00	0.00	0.00
57	76, 85, 69, 55, 47, 12, 42, 40, 27, 15	76, 40, 61, 1	2	4	0.20	0.50	0.29
58	20, 28, 30, 31, 58, 59, 61, 65, 70, 4	56, 37, 41, 20, 40	1	5	0.10	0.20	0.13
59	22, 27, 42, 11, 28, 57, 59, 62, 65, 68	76, 11, 42, 61, 22	3	5	0.30	0.60	0.40
60	1, 78, 69, 51, 43, 40, 38, 27, 20, 15	76, 74, 43, 1, 90, 15, 27	4	7	0.40	0.57	0.47
61	25, 27, 42, 11, 12, 28, 53, 57, 59, 7	22, 42, 11	2	3	0.20	0.67	0.31
62	37, 28, 13, 26, 34, 48, 52, 63, 65, 3	74, 1, 50, 67, 13, 61, 52, 48, 63, 37	5	10	0.50	0.50	0.50
63	18, 23, 24, 26, 40, 29, 47, 71, 84, 86	18	1	1	0.10	1.00	0.18
64	19, 23, 24, 34, 48, 58, 67, 71, 79, 82	19	1	1	0.10	1.00	0.18
65	20, 22, 23, 41, 26, 44, 25, 87, 4, 27	45, 69, 62, 7, 65, 20, 48, 63	1	8	0.10	0.13	0.11
66	45, 30, 44, 13, 20, 12, 27, 25, 23, 22	11, 22, 52, 48, 63, 23	2	6	0.20	0.33	0.25
67	14, 23, 32, 53, 57, 58, 66, 80, 81, 4	80, 1, 14, 90, 60, 66	3	6	0.30	0.50	0.38
68	21, 30, 22, 44, 23, 20, 52, 25, 45, 26	21, 31, 76, 20	2	4	0.20	0.50	0.29
69	4, 45, 44, 30, 27, 26, 25, 23, 22, 20	72, 74, 73, 23, 21, 20, 26, 28	3	8	0.30	0.38	0.33
70	21, 24, 26, 37, 34, 45, 53, 54, 56, 65	24, 21, 2, 60	2	4	0.20	0.50	0.29

Average					0.22	0.42	0.29
Parity with English language					85.00%	85.64%	85.22%

*Arabic language*							
1	37, 26, 28, 58, 65, 66, 83, 84, 85, 4	8, 46, 37, 41, 44, 20, 22	1	7	0.10	0.14	0.12
2	15, 24, 28, 46, 56, 4, 62, 78, 79, 81	85, 12, 46, 37, 39, 8	1	6	0.10	0.17	0.13
3	18, 19, 39, 40, 30, 34, 71, 81, 83, 86	81, 34, 72, 38, 40, 37	3	6	0.30	0.50	0.38
4	79, 77, 72, 67, 51, 46, 28, 12, 42, 24	51, 11, 12, 43	2	4	0.20	0.50	0.29
5	13, 23, 37, 40, 28, 30, 60, 81, 83, 85	83, 11, 28, 37	3	4	0.30	0.75	0.43
6	23, 39, 40, 29, 65, 67, 71 72 81 86	8, 29, 72, 28, 39, 61, 40	4	7	0.40	0.57	0.47
7	30, 22, 25, 23, 34, 28, 31, 44, 86, 3	74, 30, 65, 37, 40, 49, 25, 41, 27	2	9	0.20	0.22	0.21
8	81, 23, 39, 40, 29, 58, 3, 65, 67, 72	90, 8, 29, 61, 28, 39, 72, 40	4	8	0.40	0.50	0.44
9	20, 23, 31, 44, 45, 60, 64, 81, 3, 65,	31, 37, 13, 64, 45	3	5	0.30	0.60	0.40
10	33, 15, 19, 37, 41, 52, 89, 5, 3, 2	33, 56	1	2	0.10	0.50	0.17
11	34, 19, 48, 57, 68, 71, 81, 83, 86, 2	34, 39	1	2	0.10	0.50	0.17
12	23, 85, 58, 45, 44, 31, 30, 28, 25, 20	74, 84, 27, 44, 46, 49, 61, 45, 50	2	9	0.20	0.22	0.21
13	47, 25, 35, 37, 28, 30, 56, 69, 79, 83	44, 45, 69, 27, 25, 26	2	6	0.20	0.33	0.25
14	3, 77, 67, 63, 52, 48, 28, 37, 26, 13	47, 83, 89	0	3	0.00	0.00	0.00
15	84, 81, 72, 67, 49, 45, 31, 30, 25, 20	48, 49, 52, 26, 42	1	5	0.10	0.20	0.13
16	50, 24, 27, 36, 39, 41, 53, 68, 70, 81	49, 65, 41, 67, 30, 45, 64, 36	2	8	0.20	0.25	0.22
17	4, 15, 27, 40, 43, 12, 51, 60, 78, 1,	50, 61, 46, 44, 49, 43	1	6	0.10	0.17	0.13
18	52, 3, 63, 48, 27, 44, 42, 37, 13, 23	77, 51, 1, 15, 27, 12, 40	1	7	0.10	0.14	0.12
19	13, 23, 27, 37, 42, 44, 48, 52, 63, 3	48, 52, 63, 61, 26, 13	4	6	0.40	0.67	0.50
20	20, 53, 60, 64, 65, 70, 66, 81, 68, 85	85, 65, 41, 66	3	4	0.30	0.75	0.43
21	21, 24, 53, 54, 57, 59, 64, 68, 87, 1	54, 62	1	2	0.10	0.50	0.17
22	4, 2, 87, 85, 81, 80, 56, 55, 53, 28	80, 7, 2, 56, 41	3	5	0.30	0.60	0.40
23	86, 81, 71, 64, 59, 57, 53, 37, 25, 23	88, 87, 57, 59, 37, 65	3	6	0.30	0.50	0.38
24	27, 45, 53, 57, 58, 63, 64, 66, 84, 87	87, 54, 58, 37, 41, 65, 57	3	7	0.30	0.43	0.35
25	53, 60, 64, 65, 90, 81, 80, 70, 68, 66	80, 85, 53, 60, 66, 11, 90	5	7	0.50	0.71	0.59
26	20, 37, 45, 53, 56, 61, 62, 64, 1, 4	76, 7, 4, 61, 41, 42, 37	3	7	0.30	0.43	0.35
27	4, 64, 44, 30, 27, 25, 23, 22, 20, 13	54, 62, 65, 27	1	4	0.10	0.25	0.14
28	26, 13, 37, 44, 52, 54, 62, 63, 65, 3	48, 52, 62, 63, 37	4	5	0.40	0.80	0.53
29	23, 37, 28, 46, 49, 57, 64, 66, 72, 81	69, 48, 64, 65, 67, 85, 61	1	7	0.10	0.14	0.12
30	85, 72, 70, 68, 65, 64, 63, 13, 53, 28	69, 87, 1, 53, 65, 47, 64, 70, 85	5	9	0.50	0.56	0.53
31	31, 53, 55, 56, 60, 61, 66, 70, 81, 4	80, 90, 66, 60, 53, 14	3	6	0.30	0.50	0.38
32	4, 70, 68, 65, 64, 36, 13, 22, 27, 53	85, 76, 68, 70, 36, 80, 69, 65, 66	4	9	0.40	0.44	0.42
33	27, 30, 34, 46, 54, 57, 62, 65, 72, 86	69, 76, 1, 65, 45, 46, 61, 28	2	8	0.20	0.25	0.22
34	53, 60, 64, 65, 66, 68, 70, 81, 85, 90	68, 70, 40, 53, 72, 66, 73, 4, 41	4	9	0.40	0.44	0.42
35	19, 26, 34, 47, 53, 60, 71, 86, 2, 4	71, 39, 40	1	3	0.10	0.33	0.15
36	12, 1, 4, 13, 53, 62, 63, 74, 64, 67	74, 50, 1, 37, 13, 43, 12, 42	4	8	0.40	0.50	0.44
37	26, 36, 37, 38, 43, 52, 63, 72, 77, 86	76, 77, 61, 51, 79, 37	2	6	0.20	0.33	0.25
38	20, 24, 43, 28, 56, 77, 78, 83, 4, 66	83, 77, 78, 47	3	4	0.30	0.75	0.43
39	4, 85, 83, 80, 79, 34, 28, 11, 24, 37	79, 12	1	2	0.10	0.50	0.17
40	81, 80, 67, 66, 54, 23, 53, 49, 31, 30	80, 53, 60, 66	3	4	0.30	0.75	0.43
41	4, 81, 72, 71, 70, 29, 40, 39, 23, 18	90, 7, 81, 73, 38, 32, 39, 40, 37	3	9	0.30	0.33	0.32
42	85, 82, 77, 58, 48, 34, 36, 25, 19, 18	7, 82, 12	1	3	0.10	0.33	0.15
43	19, 25, 30, 34, 47, 49, 71, 83, 85, 86	83, 89, 47, 37, 85	3	5	0.30	0.60	0.40
44	7, 3, 84, 60, 59, 58, 28, 37, 36, 27	77, 84, 46, 44, 37	2	5	0.20	0.40	0.27
45	90, 85, 70, 68, 65, 60, 64, 59, 53, 20	53, 65, 66, 85 68, 25, 90	5	7	0.50	0.71	0.59
46	4, 86, 59, 53, 45, 34, 29, 39, 27, 26	86, 1, 43	1	3	0.10	0.33	0.15
47	87, 65, 53, 64, 63, 59, 62, 22, 15, 13	82, 62, 87, 53, 70, 14	3	6	0.30	0.50	0.38
48	88, 81, 72, 71, 29, 70, 40, 18, 39, 38	88, 43, 39	2	3	0.20	0.67	0.31
49	7, 86, 84, 66, 58, 53, 48, 32, 26, 14	89, 83, 2, 47	0	4	0.00	0.00	0.00
50		90, 29, 72, 4, 38, 66, 39	0	7	0.00	0.00	0.00
51	15, 27, 40, 11, 62, 85, 63, 86, 87, 1	74, 1, 50, 15, 37, 27, 40	4	7	0.40	0.57	0.47
52	4, 2, 1, 86, 81, 56, 55, 49, 34, 19	2, 4, 55, 56, 15	4	5	0.40	0.80	0.53
53	7, 4, 3, 81, 66, 65, 60, 53, 32, 31	7, 5, 3, 32, 37	3	5	0.30	0.60	0.40
54	23, 27, 28, 44, 48, 49, 60, 1, 3, 4	90, 2, 4, 1, 29, 84, 27, 18	3	8	0.30	0.38	0.33
55	85, 7, 57, 84, 66, 65, 60, 53, 37, 23	7, 88, 58, 59, 54, 57	2	6	0.20	0.33	0.25
56	70, 65, 62, 52, 64, 48, 29, 28, 13, 23	8, 39, 47, 46, 81	0	5	0.00	0.00	0.00
57	76, 1, 71, 69, 62, 51, 43, 40, 27, 15	76, 40, 61, 1	3	4	0.30	0.75	0.43
58	3, 87, 31, 28, 41, 37, 36, 26, 25, 20	56, 37, 41, 20, 40	3	5	0.30	0.60	0.40
59	79, 70, 67, 62, 57, 28, 11, 42, 27, 13	76, 11, 42, 61, 22	2	5	0.20	0.40	0.27
60	15, 26, 27, 40, 43, 48, 51, 69, 74, 1	76, 74, 43, 1, 90, 15, 27	4	7	0.40	0.57	0.47
61	14, 26, 31, 60, 65, 66, 74, 81, 3, 7	22, 42, 11	0	3	0.00	0.00	0.00
62	3, 74, 65, 63, 52, 48, 30, 26, 23, 13	74, 1, 50, 67, 13, 61, 52, 48, 63, 37	5	10	0.50	0.50	0.50
63	85, 72, 71, 65, 34, 30, 40, 26, 23, 18	18	1	1	0.10	1.00	0.18
64	2, 86, 85, 74, 57, 34, 29, 27, 23, 19	19	1	1	0.10	1.00	0.18
65	45, 44, 31, 30, 28, 41, 26, 23, 22, 20	45, 69, 62, 7, 65, 20, 48, 63	2	8	0.20	0.25	0.22
66	4, 45, 44, 30, 41, 25, 23, 22, 21, 20	11, 22, 52, 48, 63, 23	2	6	0.20	0.33	0.25
67	14, 22, 23 28, 49, 54, 58, 61, 66, 4	80, 1, 14, 90 , 60, 66	2	6	0.20	0.33	0.25
68	69, 67, 57, 54, 45, 25, 24, 21, 20, 15	21, 31, 76, 20	2	4	0.20	0.50	0.29
69	4, 45, 44, 30, 27, 25, 23, 22, 20, 13	72, 74, 73, 23, 21, 20, 26, 28	2	8	0.20	0.25	0.22
70	87, 81, 80, 79, 64, 57, 56, 55, 42, 24	24, 21, 2, 60	1	4	0.10	0.25	0.14

Average					0.23	0.44	0.30
Parity with English language					90.56%	89.77%	90.28%

**Table 4:** LAIR retrieval performance with selected languages against the English language.

S/N	Language	Precision@10	Recall@10	F-measure@10
1	English language	0.26	0.49	0.34

2	Yoruba language	0.20	0.41	0.27
	Parity with English language	78.89%	83.13%	80.30%

3	Igbo language	0.23	0.43	0.30
	Parity with English language	90.00%	88.87%	89.61%

4	Hausa language	0.22	0.42	0.29
	Parity with English language	85.00%	85.64%	85.22%

5	Arabic language	0.23	0.44	0.30
	Parity with English language	90.56%	89.77%	90.28%

Average performance of LAIRS		0.23	0.44	0.30
Performance of non-default languages		0.22	0.43	0.29
Overall parity with English language		86.11%	86.85%	86.35%

## Data Availability

The data used to support the findings of this study are available from the corresponding author upon request.

## Supplementary Materials

Supplementary Materials Java source code equivalent of the lingual agnostic IRS (LAIRS) architecture.
