# Correction to “A Lingual Agnostic Information Retrieval System”

**DOI:** 10.1155/tswj/9846483

**Published:** 2025-11-26

U.B. Abdullahi, G.O. Ekuobase, “A Lingual Agnostic Information Retrieval System,” *The Scientific World Journal*, 2024, https://doi.org/10.1155/2024/6949281

In the article, there is an error in Section 2. Specifically,

“CLIRS, which allows Indian natives to search English document collections in Tamil native language, was developed.”

should read as follows:

“CLIRS, which allows Indian natives to search English document collections in Tamil native language, was developed in [3].”

Additionally, the section of Table 2 showing Arabic translated queries was mistakenly omitted during the production process. The correct Table 2 is shown below:

We apologize for these errors.

## Figures and Tables

**Table 1:** Retrieved document identifiers for curated topics (former Appendix B).

**English language**		
**S/n**	**Topics**	**Retrieved document identifier**

1.	Assess and role and language and communication and transform and sustain and national and development and Nigeria	66, 44, 83, 23, 7, 20, 4, 26, 84, 37
2.	Importance and maintenance and culture and management and growth and organization and Nigeria	46, 89, 41, 7, 4, 28, 23, 62, 87, 97
3.	Role and Islamic and studies and teacher and “quality control” and education and Nigeria	18, 30, 19, 47, 83, 81, 34, 40, 39, 71
4.	Leadership and sport and management and play and role and development and Nigeria	67, 11, 68, 1, 30, 28, 42, 79, 77, 12
5.	Science and education and good and governance and Nigeria	65, 72, 11, 22, 23,31, 42,28, 59, 4
6.	Islamic and education and panacea and corruption and Nigeria	23, 27, 70, 72, 39, 67, 40, 71,81, 29
7.	“Social studies” and education and “national security” and transformation and agenda	65, 25, 67, 64, 4, 1, 31, 44, 20, 23, 30
8.	Islamic and education and panacea and corruption and Nigeria and economy	28, 49, 23, 27, 70, 42, 71, 67, 81, 29
9.	Transformation and agenda and “economic development” and Nigeria	64, 65, 44, 80, 26, 41, 30, 23, 20, 31
10.	Estimation and percentage and calcium and “tap water”	56, 19, 35, 89, 49, 37, 52, 41, 5, 33
11.	Functions and “national commission” and college and education and NCCE and teacher and Nigeria	3, 83, 40, 19, 85, 81, 57, 2, 71, 34
12.	Sociocultural and literacy and education and “national security” and management and transformation and agenda and Nigeria	4, 27, 26, 25, 22, 31, 20, 30, 23, 44
13.	“National security” and “human right” and protection and social and transformation	31, 25, 41, 21, 23, 44, 22, 30, 45, 20
14.	Importance and games and “role play” and debate and teaching and technique and “social studies”	77, 79, 36, 65, 35, 25, 30, 69, 71, 47
15.	Potential and “theatre arts” and youth and national and stability	4, 70, 66, 21, 3, 63, 52, 13, 26, 48
16.	“Social studies” and education and tool and economy and transformation and “self-reliance”	30, 81, 36, 31, 25, 80, 65, 64, 67, 49
17.	Intergroup and multicultural and society and Nigeria	55, 44, 58, 37, 22, 20, 49, 23, 26, 50
18.	Women and sport and gender and stereotype	68, 20, 40, 12, 38, 78, 1, 27, 15, 51
19.	Youth and empowerment and Kano and conflict and national and stability and involvement and remedy	36, 27, 65, 3, 37, 48, 23, 63, 13, 52
20.	Challenge and constitution and reform and “economic depression” and national and Nigeria	84, 59, 85, 81, 67, 70, 53, 65, 64, 68
21.	“Political map” and Nigeria and 1960 and “national rebirth”	65, 62, 67, 59, 68, 64, 57, 53, 21, 54
22.	Geography and education and “natural resources” and management and “national rebirth” and Nigeria	68, 65, 85, 74, 67, 62, 81, 57, 80, 56
23.	“History education” and “national rebirth” and effective	53, 72, 56, 64, 65, 59, 58, 62, 67, 57
24.	Nigeria and “historical experience” and “national rebirth” and relevance	80, 67, 62, 56, 25, 59, 53, 64, 57, 58
25.	Global and “economic depression” and lesson and 1930 and Nigeria	65, 80, 35, 81, 66, 70, 68, 53, 64, 60
26.	Attitude and “value reorientation” and sine qua non and “national rebirth” and development	54, 65, 30, 56, 15, 67, 28, 62, 64, 61
27.	“Political education” and “national security” and Nigeria and rebirth	54, 44, 23, 58, 57, 74, 59, 67, 62, 64
28.	Youth and “political awareness” and participation and implication and “national stability”	37, 3, 4, 15, 52, 48, 26, 13, 65, 63
29.	“Social studies” and civic and responsibility and education and instrument and “economic efficiency” and “national rebirth”	72, 63, 49, 65, 86, 57, 90, 67, 62, 64
30.	Constitution and reform and “economic stability” and “national rebirth” and nigeria and “social studies” and education	81, 85, 80, 68, 53, 59, 70, 67, 64, 65
31	Underdevelopment and “economic depression” and nigeria and transformation and human and natural and resources	55, 65, 68, 64, 60, 56, 23, 14, 31, 66
32.	Population and “family life” and education and constitution and reform and “economic depression” and “national rebirth”	81, 90, 67, 85, 53, 59, 70, 65, 64, 68
33.	Menace and “human trafficking” and nigeria and implication and “social study” and education	76, 80, 27, 65, 67, 22, 25, 30, 42, 69
34.	Islamic and constitutional and reform and “economic depression” and “national rebirth”	81, 85, 59, 67, 53, 68, 65, 64, 70, 90
35.	Problem and new and direction and teaching and learning and “Islamic studies” and college and education and national and development	86, 29, 23, 39, 90, 57, 35, 74, 81, 71,
36.	Church and citizenship and leadership and education and “national rebirth”	12, 59, 62, 64, 68, 72, 67, 13, 57, 74
37.	Parent and child and influence and youth and sport and participation	15, 43, 42, 69, 26, 12, 51, 63, 36, 77
38.	Role and “physical activity” and treat and prevent and overweight and obesity	30, 56, 90, 36, 28, 89, 69, 77, 83, 78
39.	Information and communication and technology and management and administration and sport and tertiary and institution	57, 37, 85, 30, 80, 77, 3, 28, 12, 79
40.	Global and economy and “vision 2020” and geography and “melt down” and education	60, 4, 49, 56, 23, 70, 31, 53, 81, 80
41.	Role and “Islamic teaching” and economy and development and Nigeria	57, 65, 70, 29, 23, 72, 40, 81, 39, 71
42.	Hitch and organization and administration and guidance and Nigerian and “secondary school” and service	80, 57, 19, 31, 37, 36, 70, 85, 89, 82
43.	Method and activity and effective and teaching and learning and science and “secondary school”	71, 57, 35, 84, 85, 25, 47, 49, 30, 83
44.	Importance and “mother tongue” and Nigeria and aspiration and economy and development	57, 49, 58, 65, 7, 15, 26, 36, 37, 84
45.	“Secondary school” and management and constitution and reform and “economic depression” and “national and rebirth” and Nigeria	57, 56, 59, 67, 70, 68, 53, 65, 64, 85
46.	Emerging and role and UBE and society and implementation and “universal basic” and education and Nigeria and “21st century”	1, 34, 2, 81, 26, 85, 80, 4, 27, 86
47.	Credible and “electoral system” and sustainable and democracy and Nigeria and constitution and reform	68, 84, 64, 67, 15, 4, 59, 53, 87
48.	Biography and contribution and “Islamic scholar” and knowledge and “Muslim society”	90, 29, 70, 39, 81, 72, 71, 40, 88, 18
49.	Practicability and system and Nigeria and transport and industry	7, 58, 14, 28, 26, 84, 32, 48, 66, 89
50.	Islamic and solution and “economic depression” and poverty	66, 71, 68, 18, 64, 60, 81, 29, 70, 90
51.	Collaboration and approach and management and women and education and sustainable and national and development	62, 86, 12, 15, 27, 56, 28, 4, 85, 1
52.	Appraisal and part-time and “adult education” and programme and geography	34, 55, 86, 81, 44, 80, 27, 4, 56, 2
53.	Agriculture and element and facilitate and growth and stability and Nigeria and economy	27, 53, 60, 65, 28, 32, 14, 13, 7, 3
54.	“Adult education” and poverty and sustainable and economy and development and Nigeria	86, 3, 37, 81, 14, 27, 60, 28, 1, 4
55.	Examination and analysis and Nigeria and “economic structure” and organization and “historical development”	37, 23, 27, 19, 85, 86, 84, 57, 66, 7
56.	Role and Arabic and education and national and stability and Nigeria and preindependence	34, 68, 28, 13, 65, 23, 71, 48, 66, 63
57.	HIV and AIDS and awareness and female and student and attitude and “sexual behavior”	85, 18, 49, 15, 13, 71, 42, 69, 27, 76
58.	“Water security” and challenge and national and transformation and agenda and Nigeria	81, 22, 21, 26, 44, 23, 31, 30, 20, 41
59.	Drug and education and leadership and governance and Nigeria	62, 59, 65, 57, 27, 11, 68, 22, 28, 42
60.	Role and Christian and moral and enforce and women and society	38, 69, 81, 1, 51, 74, 40, 27, 15, 43
61.	Examination and challenge and governance and Nigeria and economy	53, 31, 74, 23, 11, 42, 28, 65, 14, 19
62.	“Citizenship education” and youth and empowerment and national and stability	74, 4, 26, 44, 63, 23, 3, 48, 52, 13
63.	Contemporary and education and millennium and enhance and development and goal and Islamic and curriculum and education	74, 4, 26, 44, 63, 23, 3, 48, 52, 13
64.	Continuous and assessment and examination and malpractice and Nigeria and federal and college and education	76, 57, 29, 83, 27, 23, 81, 74, 34, 19
65.	“National security” and transformation and agenda and issues and challenge and Nigeria	64, 21, 22, 44, 41, 26, 31, 30, 23, 20
66.	“National security” and Nigeria and governance	31, 4, 59, 45, 20, 44, 23, 28, 22, 54
67.	Colonial and imperialism and poverty and underdevelopment and Nigeria and preindependence	7, 4, 23, 87, 63, 58, 53, 54, 66, 14
68.	“Street map” and “house number” and tool and “national security” and transformation and urban and effective and Kano	67, 41, 20, 15, 30, 23, 25, 24, 54, 21
69.	Role and “religious education” and “national security” and transformation and peace and Nigeria	65, 26, 28, 20, 22, 25, 45, 44, 30, 23
70.	Geographic and information and system and tool and “crime management” and effective and GIS and Nigeria	81, 89, 67, 42, 80, 79, 57, 21, 56, 24

**Hausa language**		
**S/n**	**Topics**	**Retrieved document identifiers**

1.	Aunawa da matsayi da harshe da sadarwa da sauyi da	13, 26, 27, 37, 44, 58, 65, 84, 85, 4
2.	Muhimmanci da gyare gyare and al'ada da shugabanci da ci gaban da ma'aikata da Nijeriya	23, 27, 37, 28, 46, 61, 62, 87, 1, 7
3.	Matsayin da addinin musulunci da nazari da Malamai da tabbatar da nagarta da ilmi da Nijeriya	18, 23, 27, 38, 39, 40, 59, 71, 72, 81
4.	Tsarin shugabanci da wasanni da jagoranci da rawa da takawa ci-gaba da Nijeriya	7, 79, 77, 51, 28, 12, 11, 42, 26, 13
5.	Ilmin kimiyya da kyakkyawan shugabanci da Nijeriya	22, 23, 42, 11, 28, 31, 54, 59, 68, 83
6.	Ilmin da addinin musulunci da mafita da cin-hanci da Nijeriya	23, 39, 40, 29, 49, 59, 70, 71, 72, 81
7.	Nazarin walwala da ilmi da tsaron kasa da kawo sauyi da tsari	4, 67, 64, 59, 30, 27, 25, 22, 20, 13
8.	Addinin musulunci da ilmi da mafita da cin-hanci da Nijeriya da tattalin arziki	81, 72, 71, 65, 59, 29, 28, 40, 39, 23
9.	Kawo sauyi da tsari da ci-gaban tattalin arziki da Nijeriya	15, 19, 23, 35, 33, 52, 59, 89, 2, 3
10.	Kididigar da kaso cikin dari da sinadarin kalsiyam da “ruwan famfo”	3, 2, 89, 59, 52, 33, 35, 23, 19, 15
11.	Ayyukan da hukumar kula da kwalejoji da ilmi da NCCE da ilmin koyarwa da Nijeriya	23, 34, 48, 57, 67, 71, 81, 85, 2, 4
12.	Al'adu-zamantakewa da ilmin da “tsaron kasa” da gudanarwa da kawo sauyi da tsari da Nijeriya	87, 85, 67, 65, 46, 44, 30, 25, 23, 22
13.	“tsaron kasa” da “hakkin dan Adam” da kare da walawala da sauyi	20, 21, 22, 23, 25, 30, 27, 44, 54, 4
14.	Muhimmancin da wasanni da “kwaikwayo” da muhawara da koyarwa da dabarun da ‘nazarin walwala”	35, 36, 30, 47, 49, 64, 65, 69, 74,83
15.	Rawar da ilmin wasannin kwaikwayo da matasa da zanan lafiyar kasa	26, 13, 22, 37, 28, 52, 63, 78, 3, 4
16.	Nazarin walwala da ilmin da hanya da tattalin arziki da sauyi “dogaro da kai”	3, 81, 80, 74, 67, 64, 49, 31, 30, 27
17.	Dangantaka tsakanin kabilu da kasa mai dauke da kabilu da yawa da al'umma da Nijeriya	64, 59, 54, 53, 50, 49, 44, 22, 14, 13
18.	Mata da wasanni da jinsi	1, 79, 78, 77, 51, 12, 40, 38, 27, 14
19.	Matasa da tallafin sana'a da Kano da rikici da na kasa da kwanciyar hankali	26, 13, 23, 37, 28, 48, 52, 57, 63, 3
20.	Matsaloli da tsarin mulki da gyara da “masassarar tattalin arziki” da na kasa da Nijeriya	28, 59, 64, 65, 67, 68, 80, 81, 87, 7
21.	“taswirar siyasar” da Nijeriya da 1960 zuwa yau da “Nijeriya sabuwa”	87, 74, 69, 68, 66, 58, 57, 54, 53, 23
22.	Nazarin bayaynin kasa da ilmin da “albarkatun kasa” da sarrafa da na kasa da “Nijeriya sabuwar”	11, 28, 34, 44, 56, 57, 67, 80, 85, 1
23.	“ilmin tarihi” da Nijeriya sabuwa da	14, 20, 57, 58, 59, 60, 65, 66, 88, 7
24.	Nijeriya da “faruwar abubuwa tarihi” da “Nijeriya sabuwa”	20, 23, 58, 65, 66, 68, 74, 69, 85, 7
25.	Na duniya da “masassarar tattalin arziki” da kalubale da 1930 da Nijeriya	65, 59, 58, 53, 44, 28, 11, 41, 27, 23
26.	Halayyar da “gangamin sauya halayya” da tafarkin “Nijeriya sabuwa da ci gaba	####
27.	“ilmin siyasa da “tsaron kasa” da Nijeriya sabuwa	###
28.	Matasa da “wayewar siyasa” da shiga a dama da sakamakon da “kwanciyar hankalin kasa”	51, 52, 63, 2, 3, 4, 13, 26, 28, 48
29.	“nazarin walwala” da na dan kasa da hakkoki da ilmi da hanyar da “habbakar tattalin arziki” da Nijeriya sabuwa	23, 26, 44, 45, 49, 58, 63, 81, 84, 4
30.	Tsarin mulki da gyara da “habakar tattalin arziki” da “Nijeriya sabuwa” Nijeriya da “nazarin walawala” da ilmi	22, 23, 42, 28, 59, 64, 65, 67, 84, 4
31	Rashin ci gaba da “masassarar tattalin arziki” da Nijeriya da sauyi da dan Adam da albarkatun da kasa	14, 31, 49, 56, 57, 58, 59, 66, 4, 7
32.	Yawan al'umma da “tsarin iyali” da ilmi da tsarin mulki da gyara da “masassarar tattalin arziki” da Nijeriya sabuwa”	11, 23, 28, 59, 65, 67, 84, 68, 87, 4
33.	Yawaitar da “safarar mutane zuwa ketare” da Nijeriya da kalubale da “nazarin walwala” da ilmi	26, 20, 23, 44, 28, 57, 63, 84, 65, 4
34.	Musulunci da tsarin mulki da gyara da “masassarar tattalin arziki” da Nijeriya sabuwa	70, 68, 67, 65, 22, 23, 40, 41, 59, 38
35.	Matsalolin da sabuwar hanyar da koyarwa da koyo da “ilmin addinin musulunci” da kwalejojin da ilmi da Nijeriya da ci gaba	23, 35, 34, 49, 57, 65, 67, 71, 83, 85
36.	Choci da zama dan kasa da shugabanci da ilmi da “Nijeriya sabuwa”	74, 68, 57, 46, 28, 12, 42, 23, 13, 22
37.	Iyaye da yara da tasiri da matasa da wasanni da shiga a dama	26, 36, 42, 12, 51, 52, 63, 77, 78, 86
38.	Rawar da “aikin motsa jiki” da magance da karewa da nauyi da yawa kiba	21, 22, 26, 11, 45, 48, 51, 78, 87, 5
39.	Hanyar sadarwa da ta zamani da gudanarwa da shugabanci da wasanni da manyan da makarantu	79, 77, 57, 66, 51, 28, 12 11, 21, 13
40.	Na duniya tattalin arziki da “burin 2020” da nazarin bayanin kasa da “durkushewa” da ilmi	23, 37, 28, 31, 56, 57, 58, 64, 65, 84
41.	Rawar da “koyar da ilmin addinin musulunci” da tattalin arziki da ci gaba da Nijeriya	14, 23, 37, 28, 59, 64, 65, 66, 3, 7
42.	Matsaloli da gudanarwa da shugabacin da jagoranci da makarantun sakandire	25, 42, 12, 28, 34, 57, 77, 82, 85, 89
43.	Dabarar koyarwa da ta shafi yi da kai da aiki da koyarwa da koyo da kimiyya da “makarantun sakandire	84, 83, 71, 57, 49, 30, 35, 26, 25
44.	Muhimmancin da “harshen uwa” da Nijeriya da buri da tattalin arziki da ci gaban	37, 28, 31, 53, 57, 60, 64, 65, 84, 4
45.	“makarantun sakandire” da shugabanci da tsarin mulki da gyaran da “masassarar tattalin arziki” da Nijeriya sabuwa da Nijeriya	82, 81, 68, 67, 65, 59, 31, 11, 42, 25
46.	Sababbin da rawan da UBE da al'umma da aiwatarwa da “ilmi na bai daya” da ilmi da Nijeriya da “karni na 21”	24, 26, 27, 34, 49, 59, 80, 84, 86, 4
47.	Na gaskiya da “tsarin zabe” da mai dorewa da Damokaradiyya da Nijeriya da tsarin mulki da gyara	15, 22, 42, 11, 28, 53, 59, 65, 67, 87
48.	Tarihin rayuwa da gudunmawar da “malamin addinin musulunci” da ilmi da “al'ummar musulmi”	37, 57, 59, 65, 71, 73, 81, 86, 88, 1
49.	Yiwuwar aiwatar da tsarin nijeriya da safuri da masana'antu	28, 32, 49, 58, 66, 77, 86, 87, 89, 3
50.	Musulunci da hanyar warware da “masassarar tattalin arziki” da talauci	18, 23, 27, 38, 29, 70, 72, 81, 90, 4
51.	Hadin guiwa da salon da gudanarwa da mata da ilmi da mai dorewa da na kasa da ci gaba	4, 1, 86, 85, 56, 28, 12, 40, 27, 15
52.	Nazarin da na wucin gadi da “ilmin manya” da nazarin bayanin kasa	27, 40, 30, 34, 56, 80, 81, 87, 2, 4
53.	Noma da sinadarin taimakawa da karuwa da daidaito da Nijeriya da tattalin arziki	20, 45, 57, 58, 60, 66, 68, 3, 4, 7
54.	“ilmin manya” da talauci da mai dorewa da tattalin arziki da ci gaban Nijeriya	14, 27, 37, 28, 60, 81, 86, 87, 1, 4
55.	Awo da nazari da Nijeriya da “tsarin tattalin arziki” da gudanarwa da”tarihin ginuwarasa”	24, 12, 28, 56, 57, 60, 63, 85, 89, 1
56.	Rawar da takawa” da larabci da ilmi da kwanciyar hankalin kasa da Nijeriya kafin ‘yancin kai	####
57.	Cutar HIV da kanjamau da masaniya da mata da dalibai da halayya da “jima'i”	76, 85, 69, 55, 47, 12, 42, 40, 27, 15
58.	“wadatuwar ruwan sha” da kalubale da na kasa da sauyi da buri da Nijeriya	20, 28, 30, 31, 58, 59, 61, 65, 70, 4
59.	Miyagun kwayoyi da ilmintarwa da jagoranci da shugabanci da Nijeriya	22, 27, 42, 11, 28, 57, 59, 62, 65, 68
60.	Rawar da kirasta da da'a da tabbatar da da mata da al'umma	1, 78, 69, 51, 43, 40, 38, 27, 20, 15
61.	Auna da kalubale da shugabanci da Nijeriya da tattalin arziki	25, 27, 42, 11, 12, 28, 53, 57, 59, 7
62.	“ilmin dan kasa nagari” da matasa da tallafawa da sana'a da kwanciyar hankalin kasa	37, 28, 13, 26, 34, 48, 52, 63, 65, 3
63.	Sababbin batutuwa da ilmi da sabon karni da taimakawa da ci gaba da manufa da musulunci da manhaja da ilmi	18, 23, 24, 26, 40, 29, 47, 71, 84, 86
64.	Gwaji da na yau da kullun da jarrabawa da magudin jarrabawa da Nijeriya da kwalejin da ilmi da tarayya.	19, 23, 24, 34, 48, 58, 67, 71, 79, 82
65.	“tsaron kasa” da sauyi buri da batutuwa da kalubale da Nijeriya	20, 22, 23, 41, 26, 44, 25, 87, 4, 27
66.	“tsaron kasa” da Nijeriya da shugabanci	45, 30, 44, 13, 20, 12, 27, 25, 23, 22
67.	Mulkin mallaka da talauci rashin ci gaba da Nijeriya da kafin ‘yancin kai	14, 23, 32, 53, 57, 58, 66, 80, 81, 4
68.	“Taswirar layuka” da “lambar gidaje” hanya ce da “tsaron kasa” da sauyi da birane da mai karfi da Kano	21, 30, 22, 44, 23, 20, 52, 25, 45, 26
69.	Rawar da “ilmin addini” da “tsaron kasa” da sauyi da zaman lafiya da Nijeriya	4, 45, 44, 30, 27, 26, 25, 23, 22, 20
70.	Tsarin bayanin taswirar kasa da hanya ce da “sarrafa laifuka'da GIS da Nijeriya	21, 24, 26, 37, 34, 45, 53, 54, 56, 65

**Arabic language**		
**S/N**	**Topics**	**Retrieved document identifiers**

1.	تقويم ودور ولغة واتصال وتحويل ويؤدي ووطني وتطوير ونيجريا	37, 26, 28, 58, 65, 66, 83, 84, 85, 4
2.	أهمية وصيانة وعادة وإدارة ونموّ ونيجيريا	15, 24, 28, 46, 56, 4, 62, 78, 79, 81
3.	دور وإسلام ودراسات والمدرس وجودة مقياس وتعليم ونيجيريا	18, 19, 39, 40, 30, 34, 71, 81, 83, 86
4.	قيادة ورياضة وإدارة ولعب ودور ونموّ ونيجيريا	79, 77, 72, 67, 51, 46, 28, 12, 42, 24
5.	علم وتربية وجيّد وحكومة ونيجيريا	13, 23, 37, 40, 28, 30, 60, 81, 83, 85
6.	الإسلام والتربية ودراهم ورشوة ونيجيريا	23, 39, 40, 29, 65, 67, 71 72 81 86
7.	علم الاجتماع وتربية وأمن الوطني وتحويل وبرنامج	30, 22, 25, 23, 34, 28, 31, 44, 86, 3
8.	الإسلام وتربية ودراهم ورشوة ونيجيريا واقتصاد	81, 23, 39, 40, 29, 58, 3, 65, 67, 72
9.	تحويل وبرنامج ونموّالاقتصادي ونيجيريا	20, 23, 31, 44, 45, 60, 64, 81, 3, 65,
10.	تقدير ونسبة مئوية والكسيوم وسدادة المياء	33, 15, 19, 37, 41, 52, 89, 5, 3, 2
11.	وظائف ولجنة الوطني وكلية وتربية وNCCE ومدرس ونيجيريا	34, 19, 48, 57, 68, 71, 81, 83, 86, 2
12.	تقاليد الاجتماعي وثقافة وتربية والأمن الوطني وإدارة وتحويل وبرنامج ونيجيريا	23, 85, 58, 45, 44, 31, 30, 28, 25, 20
13.	الأمن الوطني وحقوق الإنساني ودفاع وإجتماعي وتحويل	47, 25, 35, 37, 28, 30, 56, 69, 79, 83
14.	الأهمية من مباريات ودور لعب ومناقشة وتدريس والتقنية وعلم الاجتماعي	3, 77, 67, 63, 52, 48, 28, 37, 26, 13
15.	إمكاني ومسرح وشباب وقومي وثبات	84, 81, 72, 67, 49, 45, 31, 30, 25, 20
16.	علم الاجتماع وتربية وأداة واقتصاد وتحويل ونفس اعتمادي	50, 24, 27, 36, 39, 41, 53, 68, 70, 81
17.	عقد الجماعة وعادات كثيرة ومجتمع ونيجيريا	4, 15, 27, 40, 43, 12, 51, 60, 78, 1,
18.	المرأة ولعب ويولّد وشائعة	52, 3, 63, 48, 27, 44, 42, 37, 13, 23
19.	شباب ويمكّن في كانو ونزاع ووطني وثبات وتورط وعلاج	13, 23, 27, 37, 42, 44, 48, 52, 63, 3
20.	تحدية ودستور وتشكيل وكآبة الاقتصادي ووطني ونيجيريا	20, 53, 60, 64, 65, 70, 66, 81, 68, 85
21.	خريطة الساسي ونيجيريا و1960 ونهضة الوطني	21, 24, 53, 54, 57, 59, 64, 68, 87, 1
22.	جغرافيا وتربية ومحابد الثروات وإدارة ونهضة الوطني ونيجيريا	4, 2, 87, 85, 81, 80, 56, 55, 53, 28
23.	تاريخ التربية ونهضة الوطني وفعالية	86, 81, 71, 64, 59, 57, 53, 37, 25, 23
24.	نيجيريا وخبرة التاريخي ونهضة الوطني وثقافة وثاقة الصلة	27, 45, 53, 57, 58, 63, 64, 66, 84, 87
25.	عالمي وكآبة الاقتصادي والدر و1930 ونيجيريا	53, 60, 64, 65, 90, 81, 80, 70, 68, 66
26.	الطبيعة وقيمة التكييف ونهضة الوطني وتطوير	20, 37, 45, 53, 56, 61, 62, 64, 1, 4
27.	تربية السياسي وأمن الوطني ونيجيريا ونهضة	4, 64, 44, 30, 27, 25, 23, 22, 20, 13
28.	شباب ووعي السياسي واشتراك وتضمين وثبات الوطن	26, 13, 37, 44, 52, 54, 62, 63, 65, 3
29.	علم الاجتماع ومدني ومسئولية وتربية ووسيلة وفعالية الاقتصاد والنهضة الوطني	23, 37, 28, 46, 49, 57, 64, 66, 72, 81
30.	دستور والتشكيل وثبات الاقتصادي ونهضة الوطني ونيجيريا وعلم الاجتماع وتربية	85, 72, 70, 68, 65, 64, 63, 13, 53, 28
31	عدم النموّ وكآبة الاقتصاد ونيجيريا وتحويل وإنسان وطبعي الثروات	31, 53, 55, 56, 60, 61, 66, 70, 81, 4
32.	سكان وحياة الأسرة وتربية ودستور والتشكيل وثبات الاقتصاد ونهضة الوطني	4, 70, 68, 65, 64, 36, 13, 22, 27, 53
33.	تهديد وبيعة اللإنساني ونيجيريا وتضمين وعلم الاجتماع وتربية	27, 30, 34, 46, 54, 57, 62, 65, 72, 86
34.	إسلام ودستور وإعادة التشكيل وكآبة الاقتصاد ونهضة الوطني	53, 60, 64, 65, 66, 68, 70, 81, 85, 90
35.	مشكلة وجديد واتجاه وتعليم وتعلّم ودراسة اللإسلامية والكلية وتربية ووطن وتطوير	19, 26, 34, 47, 53, 60, 71, 86, 2, 4
36.	الكنيسة والشعبي والقيادة والتربية ونهضة الوطني	12, 1, 4, 13, 53, 62, 63, 74, 64, 67
37.	أبوين وابن وتأثير وشباب ولعب واشتراك	26, 36, 37, 38, 43, 52, 63, 72, 77, 86
38.	دور ونشاط المادي وفوض ومنع ووزن زائد وبدانة	20, 24, 43, 28, 56, 77, 78, 83, 4, 66
39.	معلومات واتصال وتكنولوجي وإدارة ولعب وعليا ومعاهد	4, 85, 83, 80, 79, 34, 28, 11, 24, 37
40.	عالمي واقتصاد ورؤيا 2020 وجغرافيا وإذبة التحتي	81, 80, 67, 66, 54, 23, 53, 49, 31, 30
41.	دور وتعليم إسلامي واقتصادي ومنوّ ونيجيريا	4, 81, 72, 71, 70, 29, 40, 39, 23, 18
42.	فترة ومؤسسة وإدارة وإرشاد ونيجيريا ومدارس الثانوية وأداء	85, 82, 77, 58, 48, 34, 36, 25, 19, 18
43.	طريقة ونشاط وفعالية وتعليم وتعلّم وعلوم ومدرس الثانوية	19, 25, 30, 34, 47, 49, 71, 83, 85, 86
44.	أهمية لهجة الأم ونيجيريا وطموح واقتصاد ونموّ	7, 3, 84, 60, 59, 58, 28, 37, 36, 27
45.	مدرسة الثانوية وإدارة ودستور وتشكيم وكآبة الاقتصد ووطن ونهضة الوطن ونيجيريا	90, 85, 70, 68, 65, 60, 64, 59, 53, 20
46.	الظن ودور و EBU ومجتمع وتنفيذ وتعليم الأساسي وتربية وقرن 21	4, 86, 59, 53, 45, 34, 29, 39, 27, 26
47.	موثوق ونظام الانتخابي وقابل للتقوية وديموقراطية ونيجيريا ودستور وتشكيل	87, 65, 53, 64, 63, 59, 62, 22, 15, 13
48.	سيرة وإسهام وعالم الإسلامي ومعرفة ومجتمع إسلامي	88, 81, 72, 71, 29, 70, 40, 18, 39, 38
49.	إجاء ونظام ونيجيريا ووسائل النقل وصناعة	7, 86, 84, 66, 58, 53, 48, 32, 26, 14
50.	إسلام وحلول وكآبة الاقتصاد وفقر	∗∗∗
51.	اشتراك واقتراي وإدارة ومرأة وتربية وقابل للتقوية ووطن وتنمية	15, 27, 40, 11, 62, 85, 63, 86, 87, 1
52.	تقييم وجزء وقت وتعليم الكبار وبرنامج وجغرافيا	4, 2, 1, 86, 81, 56, 55, 49, 34, 19
53.	الزراعة وعنصر ويسرّونموّ وثبات ونيجيريا ةاقتصاد	7, 4, 3, 81, 66, 65, 60, 53, 32, 31
54.	تعليم الكبار وفقر وقابل للتقوية واقتصاد وتنمية ونيجيريا	23, 27, 28, 44, 48, 49, 60, 1, 3, 4
55.	الامتحان وتحليل ونيجيريا وبناء اقتصادي ومنظمة ونموّ تاريخ	85, 7, 57, 84, 66, 65, 60, 53, 37, 23
56.	دور وعربية وتربية ووطن وثبات ونيجيريا وقبيل الحرية	70, 65, 62, 52, 64, 48, 29, 28, 13, 23
57.	و ووعي ونساء وطالب وموقف وطبيعة تناسليSDIAHIV	76, 1, 71, 69, 62, 51, 43, 40, 27, 15
58.	كفالة الماء وتحدي ووطني وتحويل وبرنامج ونيجيريا	3, 87, 31, 28, 41, 37, 36, 26, 25, 20
59.	دواء وتربية وقيادة وحكومة ونيجيريا	79, 70, 67, 62, 57, 28, 11, 42, 27, 13
60.	دور ومسيحي وأخلاقي ويقوّي ونساء ومجتمع	15, 26, 27, 40, 43, 48, 51, 69, 74, 1
61.	الامتحان وتحدية وحكومة ونيجيريا واقتصاد	14, 26, 31, 60, 65, 66, 74, 81, 3, 7
62.	تربية المواطنة وشباب وتفوض ووطني وثبات	3, 74, 65, 63, 52, 48, 30, 26, 23, 13
63.	معاصر وتربية وعولمة ويعزز وتنمية وهدف وإسلام ومنهج وتربية	85, 72, 71, 65, 34, 30, 40, 26, 23, 18
64.	استمرار وتقييم وامتحان وسوء التصرف ونيجيريا وفدرالية وكلية وتربية	2, 86, 85, 74, 57, 34, 29, 27, 23, 19
65.	أمن الوطني وتحويل وبرنامج وصدر وتحدية ونيجيريا	45, 44, 31, 30, 28, 41, 26, 23, 22, 20
66.	أمن الوطني ونيجيريا وحكومة	4, 45, 44, 30, 41, 25, 23, 22, 21, 20
67.	مستعمري والامبريالية وفقر وعدم النموّ ونيجيريا وعدم الحرية	14, 22, 23 28, 49, 54, 58, 61, 66, 4
68.	خريطة الشارع وترقيم المنزل وأداة وأمن الوطني وتحويل ومدني وفعّال وكانو	69, 67, 57, 54, 45, 25, 24, 21, 20, 15
69.	دور وتربية ديني وأمن الوطني وتحويل وأمن ونيجيريا	4, 45, 44, 30, 27, 25, 23, 22, 20, 13
70.	جغرافيا وإعلام ونظام وأداة وإدارة الجريمة وفعّال و SIG ونيجيريا	87, 81, 80, 79, 64, 57, 56, 55, 42, 24

