# Supplementary material for: A Lingual Agnostic Information Retrieval System
Source: ScientificWorldJournal. 2024 Jul 23;2024:6949281. doi: 10.1155/2024/6949281 (PMC11390192; doi:10.1155/2024/6949281)
Supplement: Supplementary Materials — Java source code equivalent of the lingual agnostic IRS (LAIRS) architecture. [file 6949281.f1.doc]

Lingual Agnostic Information Retrieval System (LAIRS) Java Implementation Source Code

package semantic_search;

// the code below imports the classes

import com.detectlanguage.DetectLanguage;

import com.detectlanguage.Result;

import java.io.BufferedReader;

import java.io.InputStreamReader;

import java.net.HttpURLConnection;

import java.net.URL;

import java.net.URLEncoder;

import java.io.IOException;

import java.nio.file.Path;

import java.nio.file.Paths;

import java.io.FileWriter;

import com.google.common.io.Files;

import java.io.BufferedWriter;

import org.apache.lucene.document.Document;

import org.apache.lucene.queryParser.ParseException;

import org.apache.lucene.search.ScoreDoc;

import org.apache.lucene.search.TopDocs;

import com.detectlanguage.errors.APIError;

import com.google.cloud.translate.Translate;

import com.google.cloud.translate.TranslateOptions;

import com.google.cloud.translate.Translation;

import java.io.ByteArrayInputStream;

import java.io.FileInputStream;

import java.io.InputStream;

import java.util.List;

import java.util.Scanner;

import java.util.logging.Level;

import java.util.logging.Logger;

import javax.swing.JOptionPane;

import org.json.JSONObject;

import org.json.JSONException;

// This class is used to test the indexing and search capability of lucene library.

public class Semantic_Search {

// the variables belows contains path to the index,corpus and cache folder

String indexDir = "C:\\Users\\Dell PC\\Desktop\\Lucene\\Index";

String cacheDir = "C:\\Users\\Dell PC\\Desktop\\Lucene\\Cache";

String yorubaDir = "C:\\Users\\Dell PC\\Desktop\\Lucene\\yoruba";

String hausaDir = "C:\\Users\\Dell PC\\Desktop\\Lucene\\Hausa";

String igboDir = "C:\\Users\\Dell PC\\Desktop\\Lucene\\Igbo";

String dataDir = "C:\\Users\\Dell PC\\Desktop\\Lucene\\Data";

Doc_Indexer indexer,indexer1;

Lucene_Doc_Searcher searcher,searcher1;

String z,y = "";

static String language;

public static void main(String[] args) throws APIError {

/*step one: Lanaguage Detection.

the code below uses detectlang API for language detection

*/

// the code below contains the detect language API key

DetectLanguage.apiKey = "56c86fe0513ed6d7193c492a3c23aedd";

// the code below contains the input text

String convert;

String text = JOptionPane.showInputDialog("Enter Search Text");

//String text = "معلومة";

// code below carries out language detection

language = DetectLanguage.simpleDetect(text);

System.out.println("Language Detected :"+language);

//detect language code shows the confidence, reliability

List<Result> results = DetectLanguage.detect(text);

Result result = results.get(0);

System.out.println("Language: " + result.language);

System.out.println("Is reliable: " + result.isReliable);

System.out.println("Confidence: " + result.confidence);

if(null != language) // this codes checks if the detectec language is English

switch (language) {

case "en":

Semantic_Search tester;

try {

// if the language detected is english then it indexes the corpus and hits documented with confidence scores

tester = new Semantic_Search();

convert = text;

tester.createIndex();

tester.search(convert);

} catch (IOException | ParseException e) {

} break;

//codes are missing from here.

default:

Semantic_Search tester1;

try {

tester1 = new Semantic_Search();

convert = text;

System.out.print(convert);

tester1.createIndex1();

tester1.search1(convert);

} catch (IOException | ParseException e) {

}

break;

//System.out.println("you can only search in English and Arabic, but we detected "+language+" Language in your query");

}

}

private static String translate(String langFrom, String langTo, String text) throws IOException {

// INSERT YOU URL HERE

String urlStr = "https://script.google.com/macros/s/AKfycbwrFrmUkFeZrnxPdNefU4a0v1yxCbnTXp_mQSvLayzSEMs6ci2n7ketTjZHTkU7DKiPsw/exec" +

"?q=" + URLEncoder.encode(text, "UTF-8") +

"&target=" + langTo +

"&source=" + langFrom;

URL url = new URL(urlStr);

StringBuilder response = new StringBuilder();

HttpURLConnection con = (HttpURLConnection) url.openConnection();

con.setRequestProperty("User-Agent", "Mozilla/5.0");

BufferedReader in = new BufferedReader(new InputStreamReader(con.getInputStream()));

String inputLine;

while ((inputLine = in.readLine()) != null) {

response.append(inputLine);

}

in.close();

String jsonString = response.toString();

String key = "translatedText";

String value = null;

try {

JSONObject jsonObject = new JSONObject(jsonString);

if (jsonObject.has(key)) {

value = jsonObject.getString(key);

System.out.println(value);

} else {

System.out.println("Key not found");

}

} catch (JSONException e) {

System.out.println("Invalid JSON input");

}

return value;

}

private void createIndex() throws IOException {

indexer = new Doc_Indexer(indexDir);

int numIndexed;

long startTime = System.currentTimeMillis();

numIndexed = indexer.createIndex(dataDir, new File_Filter());

long endTime = System.currentTimeMillis();

indexer.close();

System.out.println(numIndexed+" File indexed, time taken: "

+(endTime-startTime)+" ms");

}

private void createIndex1() throws IOException {

indexer1 = new Doc_Indexer(indexDir);

int numIndexed = 0;

long startTime = System.currentTimeMillis();

if(null != language)

switch (language) {

case "yo":

numIndexed = indexer1.createIndex(yorubaDir, new File_Filter());

break;

case "ig":

numIndexed = indexer1.createIndex(igboDir, new File_Filter());

break;

case "ha":

numIndexed = indexer1.createIndex(hausaDir, new File_Filter());

break;

case "ar":

numIndexed = indexer1.createIndex(cacheDir, new File_Filter());

break;

default:

System.out.println("*******");

break;

}

long endTime = System.currentTimeMillis();

indexer1.close();

System.out.println(numIndexed+" File indexed, time taken: "

+(endTime-startTime)+" ms");

}

public static void copyFile(String from, String to) throws IOException{

Path src = Paths.get(from);

Path dest = Paths.get(to);

Files.copy(src.toFile(), dest.toFile());

}

@SuppressWarnings("CallToPrintStackTrace")

private void search(String searchQuery) throws IOException, ParseException {

searcher = new Lucene_Doc_Searcher(indexDir);

long startTime = System.currentTimeMillis();

TopDocs hits = searcher.search(searchQuery);

long endTime = System.currentTimeMillis();

System.out.println(hits.totalHits +

" documents found(English search results). Time :" + (endTime - startTime));

if (hits.totalHits > 0 ) {

for(ScoreDoc scoreDoc : hits.scoreDocs) {

Document doc = searcher.getDocument(scoreDoc);

System.out.println("File: "

+ doc.get(LuceneConstants.FILE_PATH));

String filePath = doc.get(LuceneConstants.FILE_PATH);

try

{

//the file to be opened for reading

FileInputStream fis=new FileInputStream(filePath);

//returns true if there is another line to read

try (Scanner sc = new Scanner(fis) //file to be scanned

) {

//returns true if there is another line to read

while(sc.hasNextLine())

{

z = sc.nextLine();

System.out.println(z);

y = y + "\n" + z;

//returns the line that was skipped

}}

}

catch(IOException e)

{

e.printStackTrace();

}

// try (BufferedWriter writer = new BufferedWriter(new FileWriter("C:\\Users\\user\\Desktop\\Lucene\\cache\\"+ doc.get(LuceneConstants.FILE_NAME)))) {

// writer.write(y);

// writer.close();

//copyFile(doc.get(LuceneConstants.FILE_PATH),"C:\\Users\\user\\Desktop\\Lucene\\cache\\"+ doc.get(LuceneConstants.FILE_NAME));

//}

}

}

Else {

System.out.println("No result found");

}

searcher.close();

}

//this is the second search

private void searchx(String searchQuery) throws IOException, ParseException {

searcher = new Lucene_Doc_Searcher(indexDir);

long startTime = System.currentTimeMillis();

TopDocs hits = searcher.search(searchQuery);

long endTime = System.currentTimeMillis();

System.out.println(hits.totalHits +

" documents found. Time :" + (endTime - startTime));

for(ScoreDoc scoreDoc : hits.scoreDocs) {

Document doc = searcher.getDocument(scoreDoc);

System.out.println("File: "

+ doc.get(LuceneConstants.FILE_PATH));

String filePath = doc.get(LuceneConstants.FILE_PATH);

try

{

//the file to be opened for reading

FileInputStream fis=new FileInputStream(filePath);

//returns true if there is another line to read

try (Scanner sc = new Scanner(fis) //file to be scanned

) {

//returns true if there is another line to read

while(sc.hasNextLine())

{

z = translate("en", language,sc.nextLine().toLowerCase());

System.out.println("Translated text: " + z);

y = y + "\n" + z;

//returns the line that was skipped

}}

}

catch(IOException e)

{

e.printStackTrace();

}

if(null != language) // this codes checks if the detectec language is English

switch (language) {

case "yo":

try (BufferedWriter writer = new BufferedWriter(new FileWriter("C:\\Users\\Dell PC\\Desktop\\Lucene\\yoruba\\"+ doc.get(LuceneConstants.FILE_NAME)))) {

writer.write(y);

writer.close();

//copyFile(doc.get(LuceneConstants.FILE_PATH),"C:\\Users\\user\\Desktop\\Lucene\\cache\\"+ doc.get(LuceneConstants.FILE_NAME));

}

break;

case "ig":

try (BufferedWriter writer = new BufferedWriter(new FileWriter("C:\\Users\\Dell PC\\Desktop\\Lucene\\Igbo\\"+ doc.get(LuceneConstants.FILE_NAME)))) {

writer.write(y);

writer.close();

//copyFile(doc.get(LuceneConstants.FILE_PATH),"C:\\Users\\user\\Desktop\\Lucene\\cache\\"+ doc.get(LuceneConstants.FILE_NAME));

}

break;

case "ha":

try (BufferedWriter writer = new BufferedWriter(new FileWriter("C:\\Users\\Dell PC\\Desktop\\Lucene\\Hausa\\"+ doc.get(LuceneConstants.FILE_NAME)))) {

writer.write(y);

writer.close();

//copyFile(doc.get(LuceneConstants.FILE_PATH),"C:\\Users\\user\\Desktop\\Lucene\\cache\\"+ doc.get(LuceneConstants.FILE_NAME));

}

break;

case "ar":

try (BufferedWriter writer = new BufferedWriter(new FileWriter("C:\\Users\\Dell PC\\Desktop\\Lucene\\cache\\"+ doc.get(LuceneConstants.FILE_NAME)))) {

writer.write(y);

writer.close();

//copyFile(doc.get(LuceneConstants.FILE_PATH),"C:\\Users\\user\\Desktop\\Lucene\\cache\\"+ doc.get(LuceneConstants.FILE_NAME));

}

break;

default:

System.out.println("*******");

break;

}

//copyFile(doc.get(LuceneConstants.FILE_PATH),"C:\\Users\\user\\Desktop\\Lucene\\cache\\"+ doc.get(LuceneConstants.FILE_NAME));

}

private void search1(String searchQuery) throws IOException, ParseException {

searcher = new Lucene_Doc_Searcher(indexDir);

long startTime = System.currentTimeMillis();

TopDocs hits = searcher.search(searchQuery);

long endTime = System.currentTimeMillis();

System.out.println(hits.totalHits + " documents found. Time :" + (endTime - startTime));

if (hits.totalHits < 1) {

System.out.println("No Document related found in the cache that matches the search");

Semantic_Search tester1;

try {

tester1= new Semantic_Search();

String convert = translate(language,"en",searchQuery);

System.out.print("stage conversion"+convert);

tester1.createIndex();

tester1.searchx(convert);

} catch (IOException | ParseException e) {

}

}

else{

for(ScoreDoc scoreDoc : hits.scoreDocs) {

Document doc = searcher.getDocument(scoreDoc);

String filepath = doc.get(LuceneConstants.FILE_PATH);

System.out.println(" Search Results : ");

System.out.println("Document found at : " + doc.get(LuceneConstants.FILE_PATH) + "can be seen below");

try

{

//the file to be opened for reading

FileInputStream fis=new FileInputStream(filepath);

//returns true if there is another line to read

try (Scanner sc = new Scanner(fis) //file to be scanned

) {

//returns true if there is another line to read

while(sc.hasNextLine())

{

z = sc.nextLine();

System.out.println(z);

y = y + "\n" + z;

//returns the line that was skipped

}}

}

catch(IOException e)

{

e.printStackTrace();

}

//copyFile(doc.get(LuceneConstants.FILE_PATH),"C:\\Users\\user\\Desktop\\Lucene\\cache\\"+ doc.get(LuceneConstants.FILE_NAME));

}

}

searcher.close();

}

}
